# A high-quality pseudo-phased genome for *Melaleuca quinquenervia* shows allelic diversity of NLR-type resistance genes

**DOI:** 10.1093/gigascience/giad102

**Published:** 2023-12-14

**Authors:** Stephanie H Chen, Alyssa M Martino, Zhenyan Luo, Benjamin Schwessinger, Ashley Jones, Tamene Tolessa, Jason G Bragg, Peri A Tobias, Richard J Edwards

**Affiliations:** School of Biotechnology and Biomolecular Sciences, UNSW Sydney, Kensington NSW 2052, Australia; Research Centre for Ecosystem Resilience, Botanic Gardens of Sydney, Sydney NSW 2000, Australia; School of Life and Environmental Sciences, The University of Sydney, Camperdown NSW 2006, Australia; Research School of Biology, The Australian National University, Canberra ACT 2601, Australia; Research School of Biology, The Australian National University, Canberra ACT 2601, Australia; Research School of Biology, The Australian National University, Canberra ACT 2601, Australia; Research School of Biology, The Australian National University, Canberra ACT 2601, Australia; School of Environment and Rural Science, University of New England, Armidale NSW 2351, Australia; Research Centre for Ecosystem Resilience, Botanic Gardens of Sydney, Sydney NSW 2000, Australia; School of Biological, Earth and Environmental Sciences, UNSW Sydney, Kensington NSW 2052, Australia; School of Life and Environmental Sciences, The University of Sydney, Camperdown NSW 2006, Australia; School of Biotechnology and Biomolecular Sciences, UNSW Sydney, Kensington NSW 2052, Australia; Minderoo OceanOmics Centre at UWA, UWA Oceans Institute, University of Western Australia, Crawley WA 6009, Australia

**Keywords:** NLR, resistance genes, *Melaleuca quinquenervia* genome, FindPlantNLRs, broad-leaved paperbark

## Abstract

**Background:**

*Melaleuca quinquenervia* (broad-leaved paperbark) is a coastal wetland tree species that serves as a foundation species in eastern Australia, Indonesia, Papua New Guinea, and New Caledonia. While extensively cultivated for its ornamental value, it has also become invasive in regions like Florida, USA. Long-lived trees face diverse pest and pathogen pressures, and plant stress responses rely on immune receptors encoded by the nucleotide-binding leucine-rich repeat (NLR) gene family. However, the comprehensive annotation of NLR encoding genes has been challenging due to their clustering arrangement on chromosomes and highly repetitive domain structure; expansion of the NLR gene family is driven largely by tandem duplication. Additionally, the allelic diversity of the NLR gene family remains largely unexplored in outcrossing tree species, as many genomes are presented in their haploid, collapsed state.

**Results:**

We assembled a chromosome-level pseudo-phased genome for *M. quinquenervia* and described the allelic diversity of plant NLRs using the novel FindPlantNLRs pipeline. Analysis reveals variation in the number of NLR genes on each haplotype, distinct clustering patterns, and differences in the types and numbers of novel integrated domains.

**Conclusions:**

The high-quality *M. quinquenervia* genome assembly establishes a new framework for functional and evolutionary studies of this significant tree species. Our findings suggest that maintaining allelic diversity within the NLR gene family is crucial for enabling responses to environmental stress, particularly in long-lived plants.

## Background

**Figure 1:**
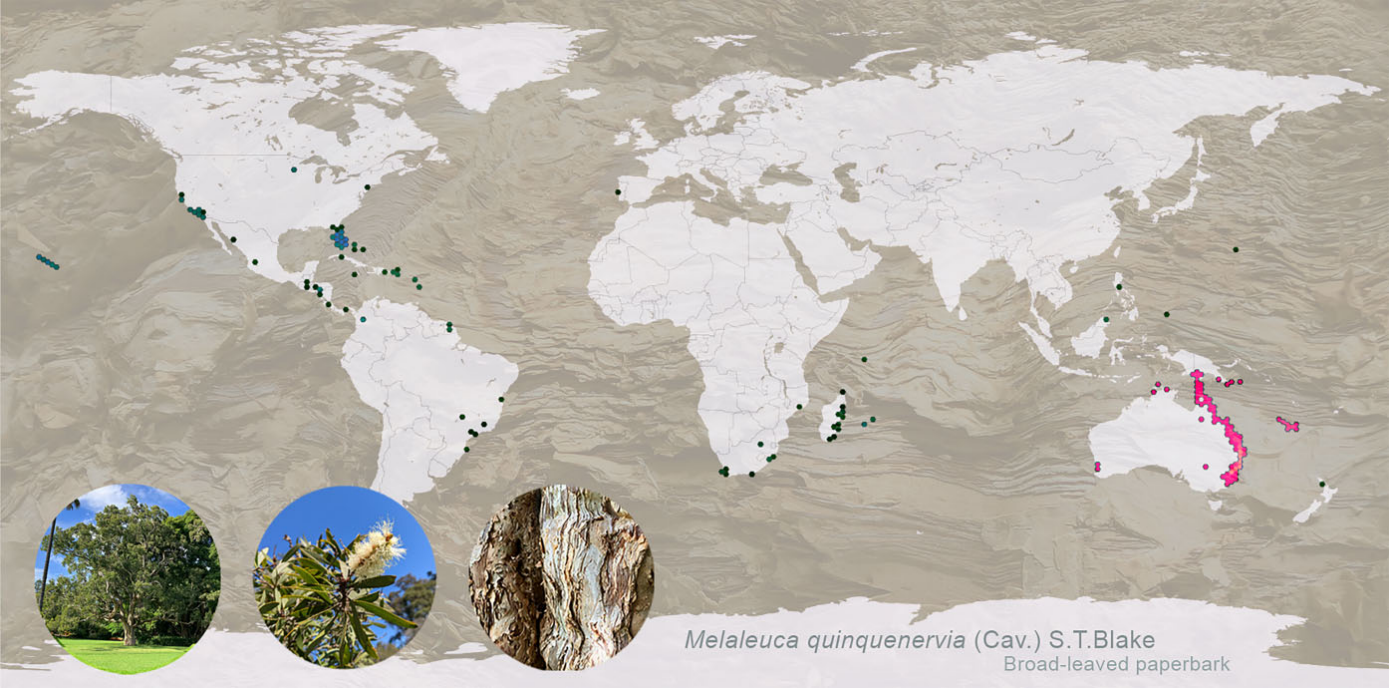
Global distribution of *Melaleuca quinquenervia* in its native range (Australia, Papua New Guinea, New Caledonia, and Indonesia; pink dots) and introduced range (blue dots). Data sourced from GBIF with darker shades indicative of higher record densities. Map generated using OpenStreetMap, licensed under the Open Data Commons Open Database License. Photos of the genome tree and detail of bark used in map background taken in the Royal Botanic Garden Sydney by S.H. Chen and P.A. Tobias.

*Melaleuca quinquenervia* (Cav.) S.T. Blake [1] is a broad-leaved paperbark tree endemic to the wetlands of eastern Australia, Papua New Guinea, New Caledonia, and Indonesia (Fig. 1) [2]. *Melaleuca quinquenervia* belongs to the family Myrtaceae, a large family of woody flowering plants consisting of over 144 genera and 5,500 species [3] with the genus *Melaleuca* comprising almost 300 species [2]. While *M. quinquenervia* is a keystone species in its native range, it is planted extensively as an ornamental and is commercially important as a source of essential oils and nectar for honey [2]. The species has become highly invasive in the wetlands of Florida in the United States following its introduction as an ornamental in the early 1900s [4] and has increased fire risk and caused the significant loss of native vegetation and associated biodiversity in wetland areas [5]. The management of *M. quinquenervia* outside its native range has a serious economic impact due to labor-intensive management practices, including site monitoring, the physical removal of trees, and herbicide application [4]. High-accuracy reference genomes are important for molecular and evolutionary studies, as well as providing a tool for strategic management of native and invasive species. With no current genome resource for *M. quinquenervia*, molecular research has been limited to homology-based studies using plants within the Myrtaceae family, including the closely related species *Melaleuca alternifolia* [6–8].

Long-living tree species, such as *M. quinquenervia*, are exposed to extensive biotic stresses over their lifetime [9], including a wide range of pests and pathogens. Plants employ various strategies to combat pests and pathogens. These include preformed physical barriers, such as leaf cuticles [10, 11] and changes in leaf anatomy [12], and chemical barriers such as secondary metabolites [13, 14]. At a molecular level, plants rely on an innate immune system to recognize and respond to pathogens [15]. The plant immune system can be considered as 2 distinctly activated but interplaying pathways involving crosstalk between pathogen and host [16]. Research has therefore focussed on understanding the molecular basis of host tree responses to inform management, with a key emphasis on recognition and response to invasion patterns [17].

There has been substantial research focused on understanding the rapid, cascading response leading to programmed cell death, initiated by resistance receptors of the nucleotide-binding leucine-rich repeat (NLR) domain type [18]. The genes encoding NLRs are a large group of plant resistance genes and are modular in their structure, generally containing 3 main domains: a nucleotide binding (NB) domain, an N-terminal domain, and a C-terminal domain. The NB site, or NBARC (Apaf-1, R-protein, and CED-4), is highly conserved in plants, having an important role in activation of the hypersensitive response (HR), which blocks disease progression by stimulating programmed cell death within and around the infected region [19]. Of the 8 motifs constituting the NBARC, the P-loop motif is the most highly conserved, being essential for ATP hydrolysis and NLR function [20]. The NLR N-terminal domain is commonly a Toll/interleukin 1 receptor/resistance protein (TIR) domain, a coiled–coil (CC) domain, or a RESISTANCE TO POWDERY MILDEW 8–like coiled–coil (RPW8/CC-R) domain [21]. Studies have demonstrated an important role for this domain for pathogen recognition and signaling [22, 23]. Plant NLRs also contain leucine-rich repeats (LRRs), which are subject to strong diversifying selection and show high sequence diversity even within closely related genes [24]. Studies suggest the high diversity of this region is the result of coevolution between host and pathogen, with several studies showing specific pathogen ligand interaction at this site.

While NLRs share common domains, they are highly diverse, even within the well-studied model species *Arabidopsis thaliana* [25]. Adding to this diversity is the addition of novel integrated domains (IDs), which can be numerous within a NLR protein and are located at various locations within the modular structure of these proteins [26]. Mimicking host proteins, evidence suggests that these domains function as decoy targets for pathogen secreted molecules, known as effectors, allowing for host recognition and triggering immune signaling [27]. A well-documented example is the RRS1 NLR in *A. thaliana*, which carries a WRKY domain [28]. It interacts with RPS4 to recognize effectors from a range of pathogens, with the pair forming a complex that is activated upon targeting/modification of the WRKY domain [28]. Without this recognition, pathogen effectors were found to inhibit host WRKY DNA binding that plays a role in defense signaling, indicating a role for the ID as a decoy [28]. Other notable examples include RGA5 and Pik-1 in rice, which both contain a heavy metal–associated domain that recognizes effectors from the rice blast pathogen *Magnaporthe oryzae* [29, 30].

NLR genes are also known to be numerous in many plant genomes [31], representing over 2% of all genes in apple (*Malus domestica*) [32]. While initial studies computationally identified 149 putative NLR-type genes in the genome of *A. thaliana* [33], more recently, a core set of 106 NLR orthogroups (6,080 genes) has been established across 52 plant accessions largely found in Europe [25] showing the incredible diversity of these genes within a single species. Despite the importance of this gene family in determining plant disease resistance, only 481 genes from 31 species have been fully or partially functionally characterized [34].

Overcoming the challenges associated with assembling these highly polymorphic and repetitive genes has been aided by sequencing technologies such as Oxford Nanopore Technologies (ONT) and PacBio HiFi [35, 36]. By facilitating the generation of more contiguous genome assemblies, these technologies allow for greater characterization and evolutionary analysis of NLR genes. This was highlighted in a recent analysis of an updated reference genome of barley [37], which revealed over double the number of NLR genes compared to previous assemblies generated with short reads [38, 39]. It has also aided in the generation of a near-complete NLRome in *A. thaliana*, allowing for the mapping of NLR genes that were previously uncharacterized [25].

The genomes of many diploid organisms are represented as collapsed consensus sequences from homologous chromosomes [40]. Owing to the highly repetitive nature of plant NLRs, detailed genome-wide analysis of NLR allelic variation has yet to be carried out. Studies have revealed extensive allelic variation in *NLR* genes such as 8 brown planthopper resistance genes in *Oryza sativa* [40]. These results indicate the importance of detailed analysis of both chromosome sets to more accurately characterize NLRs, with the outcomes having implications for plant/pathogen coevolution and informing downstream molecular analyses. Recent developments in sequencing and scaffolding methods [41] provide the opportunity to generate phased genomes of highly heterozygous organisms such as *M. quinquenervia* [6, 42].

Here we present a chromosome-level and pseudo-phased diploid genome assembly for *M. quinquenervia*. We make available FindPlantNLRs [43], a novel pipeline to fully annotate putative NLR genes, taking a genome file as the starting point . We compare NLR allelic variance within the phased, chromosome-level genome assembly of *M. quinquenervia* to provide the first example, to our knowledge, of NLR diversity in a diploid tree genome. Our data indicate that copy number, presence/absence, and integrated domains are highly variable between haplotypes. These findings reveal the high level of diversity that exists for NLRs within a single plant genome. With much of this lost in a collapsed form, we demonstrate the importance of our approach to assist research into plant responses to environmental challenges.

## Analyses

### A high quality pseudo-phased genome assembly for *Melaleuca quinquenervia*

**Figure 2:**
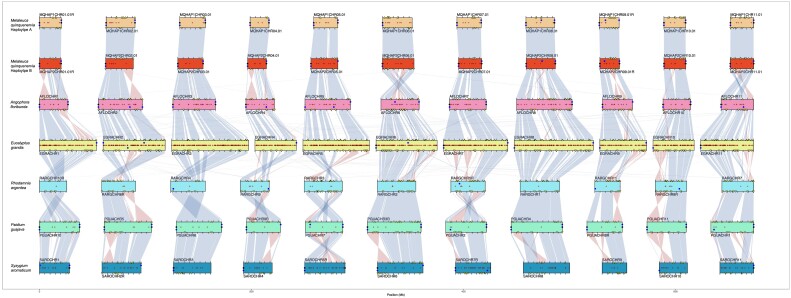
Synteny between *Melaleuca quinquenervia* phased genome and selected chromosome-level Myrtaceae genomes (*Angophora floribunda, Eucalyptus grandis, Rhodamnia argentea, Psidium guajava*, and *Syzygium aromaticum*). Synteny blocks of collinear “Complete” BUSCO genes link scaffolds from adjacent assemblies: blue, same strand; red, inverse strand. Yellow triangles mark “duplicated” BUSCOs. Filled circles mark telomere predictions from Diploidocus (black) and tidk (blue). Assembly gaps are marked as dark red + signs.

We sourced leaf material from a mature *M. quinquenervia* tree growing at the Royal Botanic Garden (RBG) in Sydney, New South Wales, for use as the reference genome. The tree was planted in 1880, is 140 years old and of unknown provenance, and is a vouchered specimen of the RBG living collections. High molecular weight DNA was extracted for PacBio HiFi and ONT sequencing. Fresh leaf samples were sent for Hi-C library preparation and sequencing. We assembled the *M. quinquenervia* genome with HiFiasm [44] using HiFi sequencing data and integrating Hi-C data, with a total yield of 19.46 Gb and 116.4 Gb reads, respectively (Table 1). We independently scaffolded the resulting pseudo-phased outputs using the Aidan Lab pipelines [45–46] and determined each haplotype comprising 11 chromosomes with 94% of sequences assigned to chromosomes for both haplotypes (Supplementary Fig. S1A, B). To independently verify the HiFi assemblies, we assembled and scaffolded the ONT data (Supplementary Fig. S1C, D), which showed a high degree of synteny to the HiFi assemblies (Supplementary Fig. S2A, B). Our final assembly genomes were 269,244,392 bp and 271,680,404 bp for haplotypes A and B, respectively (Table 2). We used Chromsyn [47] to investigate synteny of *M. quinquenervia* to 5 chromosome-level Myrtaceae genomes, all with 2*n* = 22 chromosomes (Fig. 2). The scaffolding of haplotype A is supported by the scaffolding of haplotype B for *M. quinquenervia*, despite the processes being run independently. We determined some inversions against the other Myrtaceae genome chromosomes that likely represent misassemblies in the less contiguous assemblies (Fig. 2).

**Table 1:** Genomic sequence reads for the *Melaleuca quinquenervia* genome

Sequencing platform	Library	Median insert size (bp)	Mean read length (bp)	No. of reads	Sequence bases (Gb)
PacBio Sequel II	HiFi SMRTbell	16,506	17,058	1,140,849	19.46
Illumina NextSeq 500*	Phase Genomics Proximo Hi-C (Plant)	—	2 × 151	770,901,164	116.4
Oxford Nanopore Technologies	Ligation (SQK-LSK110)	—	26,803	2,400,431	64.68
**Total gDNA**	**—**	**—**	**—**	**774,442,444**	**200.5**

* Includes a pilot iSeq run used to quality-control the library.

**Table 2:** Genome statistics for the *Melaleuca quinquenervia* phased reference genome

Statistic	Haplotype A	Haplotype B
**Total length (bp)**	269,244,392	271,680,404
**No. of scaffolds**	196	183
N50 (bp)*	22,766,892	22,112,861
L50^^†^^	6	6
**No. of contigs**	251	241
N50 (bp)*	7,525,323	5,650,000
L50^^†^^	14	16
No. of gaps	55	58
GC (%)	40.38	40.51
**BUSCO complete (genome; *n* = 1,614)**	**99.1% (1,599)**	**98.8% (1,595)**
Single copy (genome)	98.0% (1,581)	97.7% (1,577)
Duplicated (genome)	1.1% (18)	1.1% (18)
BUSCO fragmented (genome)	0.6% (9)	0.7% (12)
BUSCO missing (genome)	0.3% (6)	0.5% (7)
**Protein-coding genes (GeMoMa)**	28,744	28,517
mRNAs	43,219	42,866
rRNAs	574	1,928
tRNAs	433	422
**NBARCs (FindPlantNLRs annotation)**	**762**	**733**
**NLRs**	**676**	**652**
**BUSCO complete (proteome; *n* = 1,614)**	**99.7% (1,610)**	**99.7% (1,610)**
Single copy (proteome)	84.9% (1,371)	85.0% (1,372)
Duplicated (proteome)	14.8% (239)	14.7% (238)
BUSCO fragmented (proteome)	0.1% (2)	0.1% (2)
BUSCO missing (proteome)	0.2% (2)	0.2% (2)
**Merqury QV**	**62.3**	**62.3**
**Repeats**	**33.1%**	**33.9%**

* At least half of the bases occur in a contig/scaffold of N50 bp or greater.

† L50 is the number of contigs/scaffolds of length N50 bp or greater.

We checked the genome outputs using Depthsizer [48] using HiFi and ONT reads to show a genome size of approximately 274 Mb and 272 Mb for haplotypes A and B, respectively, with the ONT assembly giving similar figures (Supplementary Table S1). We further validated the genome size using GenomeScope [49], which predicted a haploid genome size of 262 Mb (Supplementary Fig. S3A). We confirmed the diploid state of the genome using SmudgePlot [50] (Supplementary Fig. S3B).

To improve the overall quality of the *M. quinquenervia* genomes, we carried out several rounds of scaffolding, polishing, and gap filling, with telomeres predicted by both Diploidocus [48] and tidk [51] at the end of chromosome scaffolds in most instances (Supplementary Fig. S2A, B). There are only a small number of gaps (fewer than 60) (Supplementary Fig. S2A, B).

**Figure 3:**
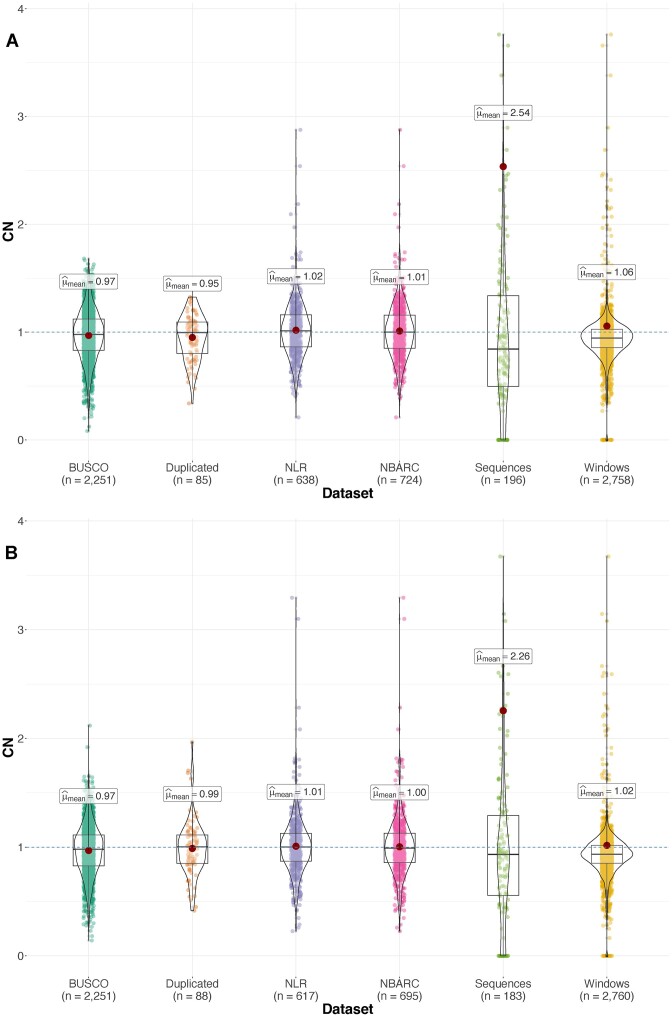
Genome-wide regional copy number analysis for *Melaleuca quinquenervia* (A) haplotype A and (B) haplotype B using HiFi read data. Copy number (CN) is relative to a single diploid (2*n*) copy in the genome. Violin plots and means generated with ggstatsplot. Each data point represents a different genomic region: BUSCO, BUSCO v5 (MetaEuk) single-copy “Complete” genes; Duplicated, BUSCO v5 “duplicated” genes; NLR, resistance gene annotations; NBARC, NBARC domains; Sequences, assembly scaffolds; and Windows, 100-kb nonoverlapping windows across the genome. Plot truncated at CN = 4.

Base pair–level accuracy was tested against Merqury [52], with both haplotypes showing very high-quality and accuracy scores. Additionally, we determined very high genome completeness of both haplotypes using BUSCO [53] (Table 2, Fig. 3A, B, Supplementary Fig. S4A–F). We ran GeMoMa [54] annotation on the 2 haplotypes, and both proteomes were 99.7% complete according to BUSCO. We assessed the repetitive as well as transfer RNA (tRNA) and ribosomal RNA (rRNA) elements using RepeatModeler [55] (Table 2).

### A novel pipeline to identify and classify NLRs

**Figure 4:**
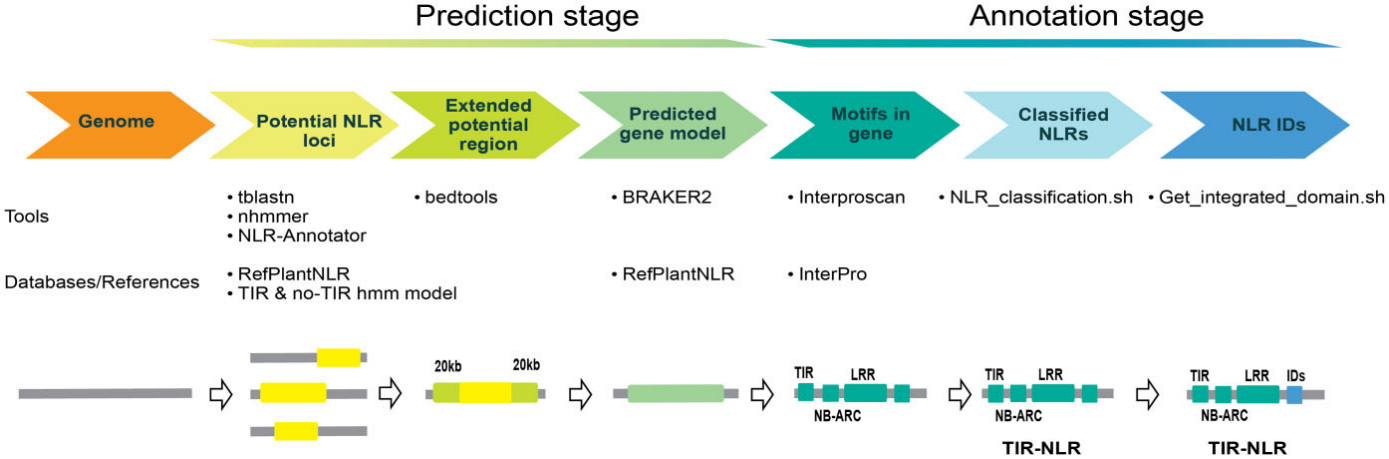
Workflow of the FindPlantNLRs pipeline: a tool for annotating nucleotide binding and leucine-rich repeat (NLR) genes. The pipeline annotates predicted NLR genes from an unmasked genome fasta file input. We combine loci identified using NLR annotator software with a basic local alignment search tool (tblastn) using recently compiled and functionally validated NLR amino acid sequences and a nucleotide iterative hidden Markov model (HMM) to locate NBARC domains in genomes. The loci identified (including 20-kb flanking regions) are then annotated with Braker2 software using protein hints from experimentally validated resistance genes. Annotated amino acid fasta files are screened for domains using Interproscan, and the predicted coding and amino acid sequences containing both NB-ARC and LRR domains are located back to scaffolds and extracted in gff3 format.

We developed a comprehensive pipeline to annotate predicted NLR genes from an unmasked genome fasta file input (Fig. 4). The rationale for an unmasked sequence is that with the repetitive nature of the NLRs, regions may be missed with standard annotations [56]. Our pipeline, named FindPlantNLRs [43], uses 3 key approaches. We combined loci identified using (i) NLR annotator software [57] with (ii) a basic local alignment search tool (tblastn) [58] using recently compiled and functionally validated NLR amino acid sequences and (iii) a nucleotide iterative hidden Markov model (HMM) [59] to locate NBARC domains in genomes [60, 61]. While the pipeline was developed to seek NLR genes within Myrtaceae genomes, the supplied NBARC HMMs are suitable for any plant genome search due to the iterative step that builds a unique species-specific HMM combined with the use of 2 other steps that incorporate broader models. The loci identified through these methods, and including 20-kb flanking regions, are then annotated with Braker2 software [62] using protein hints from experimentally validated resistance genes [34]. Annotated amino acid fasta files are screened for domains using Interproscan [63] and the predicted coding and amino acid sequences containing both NBARC and LRR domains are located back to scaffolds and extracted using additional scripts available on GitHub. To identify all classes of annotated NLRs, we developed a script that sorted and classified the “gene” types. We ran the file outputs from FindPlantNLRs with the NLR classification script [43]. To further identify novel predicted integrated domains in the annotated NLRs, we developed a script to search the data based on Pfam domain identities not classically associated with NLRs [43]. While our analyses have focused on full-length NLRs, output from the pipeline also includes truncated NB-containing genes. These files have been made available on GigaDB for future analyses.

### NLR number is variable across chromosomes and haplotypes

Using the FindPlantNLRs pipeline, we identified 762 putative NBARC-containing genes in haplotype A and 733 in haplotype B based on the presence of the NBARC domain (Supplementary Table S2). As canonical NLRs require both NBARC and LRR regions to be functional, for downstream analyses, we were interested in isolating full gene models (genes containing both domains). Termed NLRs from hereon, we have divided these into genes containing a TIR domain (TNL), a CC or Rx domain (CNL), and those lacking TIR or CC domains (NL). Of the 762 NBARC-containing genes in haplotype A, we predicted 676 NLRs, of which 67 lacked an N-terminal CC or TIR domain (Supplementary Table S3). We excluded 86 predicted genes as they did not fit the definition of full gene models, with 68 lacking a C-terminal LRR domain and 18 lacking both N- and C-terminal domains (Supplementary Table S2). Of the 733 NBARC-containing genes in haplotype B, we predicted 652 full gene models, of which 71 lacked an N-terminal CC or TIR domain (Supplementary Table S3). We excluded 81 predicted genes as they did not fit the definition of full gene models, with 61 lacking a C-terminal LRR domain and 20 lacking both N- and C-terminal domains (Supplementary Table S2).

**Figure 5:**
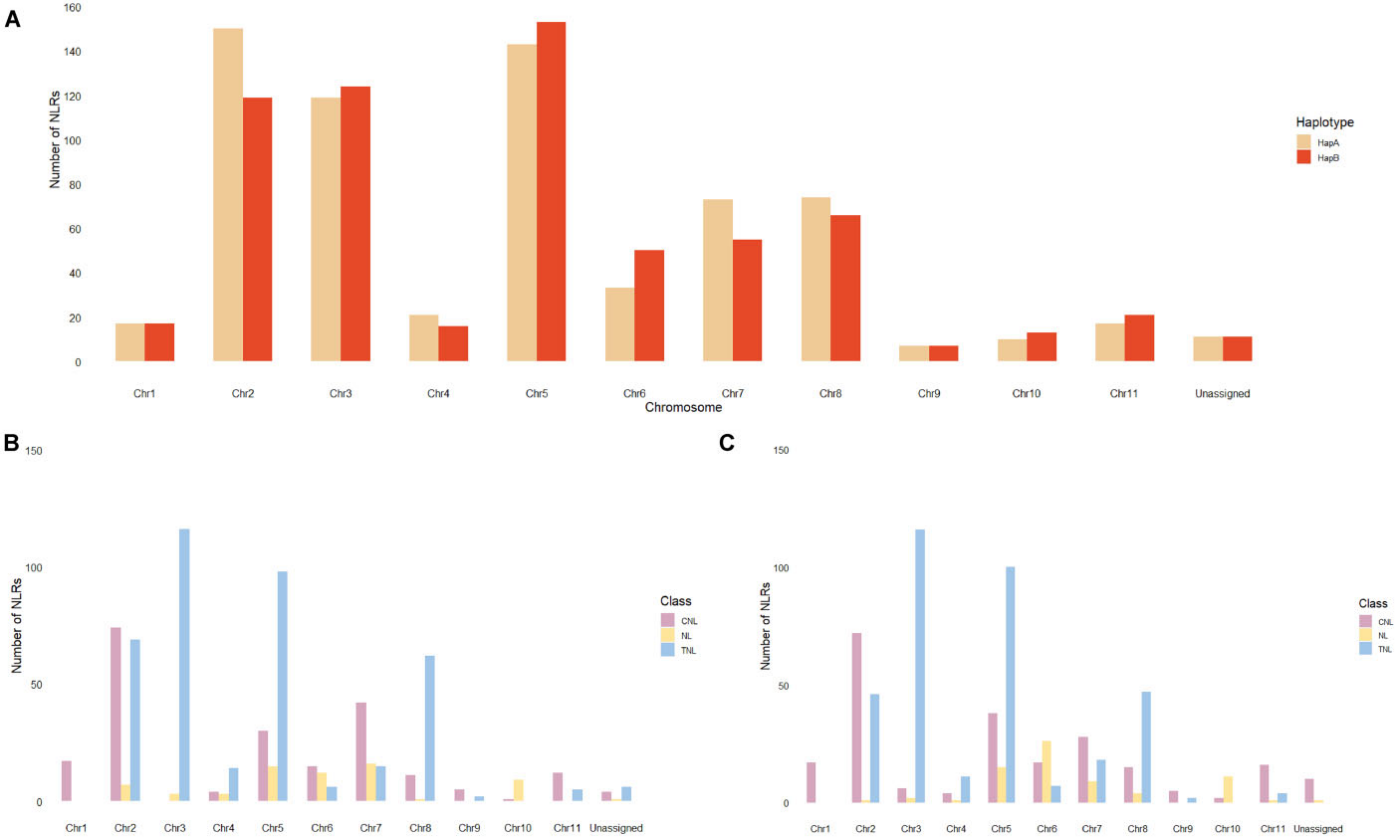
Summary of the number of predicted NLR genes per chromosome in the phased *Melaleuca quinquenervia* genome. (A) Comparison of the number of putative NLR genes on each chromosome in haplotypes A and B. Putative NLRs were classified into TIR-NLR (TNL), CC-NLR and Rx-NLR (CNL), and NL classes on individual chromosomes in (B) haplotype A and (C) haplotype B.

As NLR numbers differed between haplotypes, we sought to further investigate this difference at the chromosome level. The number of NLR genes per chromosome varied by up to 31 genes between haplotypes, with only chromosomes 1 and 9 containing the same number of genes across haplotypes (Fig. 5A). In haplotype A, chromosome 2 contained the highest number of NLR genes followed by chromosomes 5 and 3, while chromosome 5 contained the highest number of genes followed by chromosomes 3 and 2 in haplotype B (Fig. 5A). Upon further investigation, we determined the classes of NLRs is also consistent across chromosomes 1 and 9, while on all other chromosomes, the number of NLRs in each class is variable (Fig. 5B, C). Chromosome 1 was also the only chromosome to contain NLRs of 1 class (CNL) (Fig. 5B, C).

### NLR genes are arranged in clusters with hotspots on chromosomes

**Figure 6:**
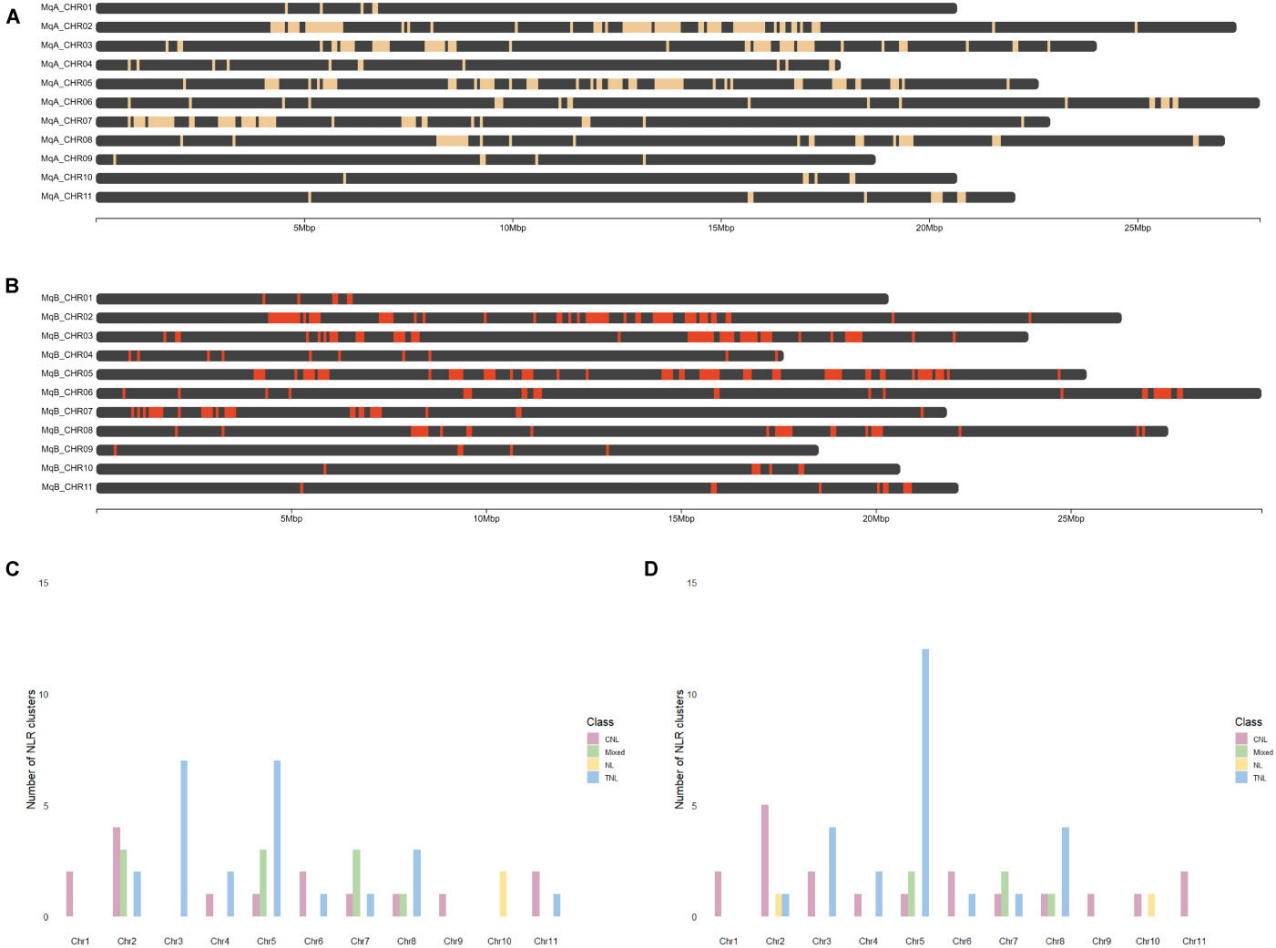
Physical clustering of predicted NLR genes in the phased *Melaleuca quinquenervia* genome. Physical locations of predicted NLR genes on the chromosomes of *Melaleuca quinquenervia* (A) haplotype A and (B) haplotype B generated using ChromoMap in RStudio. The number of clusters per chromosomes in (C) haplotype A and (D) haplotype B was analyzed and categorized based on the classes of all NLR genes.

To visualize the physical clustering of NLRs on chromosomes, we mapped gene locations to chromosomal locations in both haplotypes (Fig. 6A, B). Employing the definition of a cluster as being a genomic region with 3 NLRs less than 250 kb apart with fewer than 8 other genes between each NLR, we determined variation in the number of genes clustering per haplotype and clusters per chromosome within and between haplotypes. At a gene level, we determined 89.8% of genes in haplotype A and 90.5% of genes in haplotype B occur in clusters. A total of 51 clusters were identified in haplotype A with an average of 4.6 clusters per chromosome and an average of 11.7 genes per cluster. A total of 50 clusters were identified in haplotype B, averaging 5 clusters per chromosome and an average of 11.4 genes per cluster. Of the genes, 5.1% were determined to occur as singles in haplotype A and 5.1% as pairs, and 6.1% of genes in haplotype B were determined to occur as singles and 3.4% as pairs. In both haplotypes, the most clusters were on chromosome 5 (11 and 15 on haplotypes A and B, respectively) and the least (1 cluster) on chromosome 9 in both haplotypes (Fig. 6A, B). The independently assembled and annotated assemblies based on ONT data verified the location of the majority of NLRs (Supplementary Fig. S5).

To investigate the role of assembly quality and completeness on NLR identification and clustering, we identified the closest ortholog in the other haplotype for each NLR gene and plotted these relationships along with the positions of assembly gaps (Supplementary Figs. S6 and S7). While a few NLR clusters had assembly gaps in one or other haplotype, there were no obvious cases where a haplotype-specific expansion could be explained by a gap corresponding to the homologous region (Supplementary Figs. S7 and S8). We then determined if these clusters comprised genes of the same class. We defined classes of clusters by clusters containing only genes of 1 class along with *NL*-type genes; otherwise, they were considered mixed. TNL-type clusters were the most abundant clusters in both haplotypes and most abundant on chromosomes 3 and 5 in haplotype A and chromosome 5 in haplotype B (Fig. 6C, D). CNL-type clusters were more evenly distributed across chromosomes in both haplotypes, with chromosome 2 containing the most clusters (4 in haplotype A and 5 in haplotype B) (Fig. 6C, D).

### Integrated domains are unique between haplotypes

**Figure 7:**
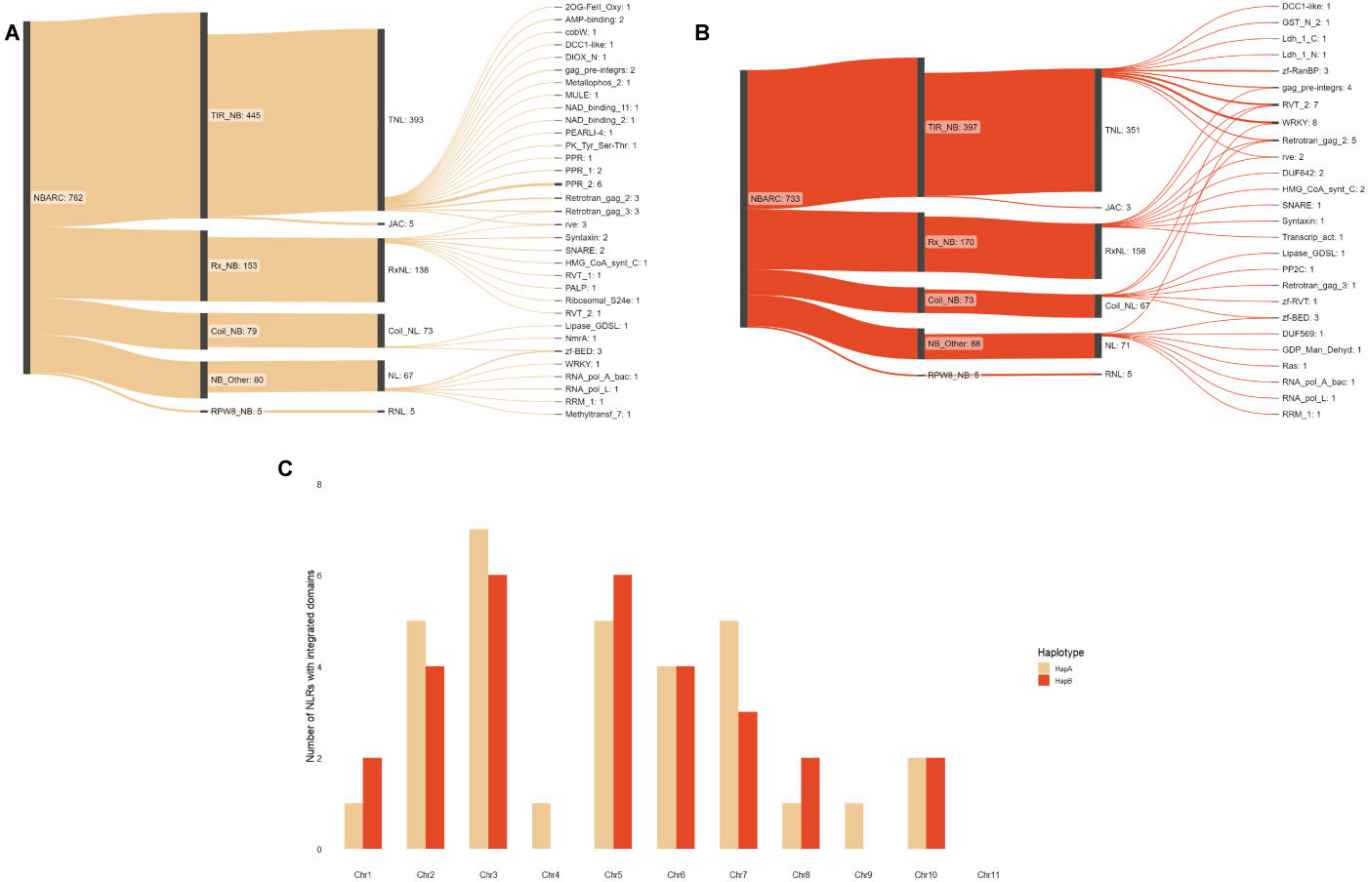
The NLR gene complement in the phased *Melaleuca quinquenervia* genome. The 2 sets of chromosomes corresponding to (A) haplotype A and (B) haplotype B were independently classified and visualized to present the domain classes using Sankeymatic [64], including the types and counts of IDs with abbreviations derived from the Pfam database [43]. NB, nucleotide binding domain; TIR, Toll/interleukin 1 receptor; JAC, jacalin domain; Rx, potato CC-NB-LRR protein Rx; Coil, coil–coil domain; RPW8, RESISTANCE TO POWDERY MILDEW 8–like coiled–coil. (C) The number of ID-containing NLRs per haplotype and chromosome in both haplotypes.

Based on Pfam domain identities of the predicted NLR genes, we discovered 4.8% of NLRs in haplotype A contained IDs (Fig.   7A), of which 44% contained more than 1 unique domain. Similarly, we observed a comparable percentage of 4.5% in haplotype B (Fig. 7B), with 37% of the predicted genes containing multiple unique domains. We also examined the number of ID-containing NLRs per chromosome and noted that in haplotype A, chromosome 3 had the highest count with 7 while chromosome 11 had none. In haplotype B, chromosome 3 had 6 ID-containing NLRs, and 11 also had none (Fig. 7C). During our investigation, we identified 48 unique IDs across both haplotypes. Interestingly, we found 23 IDs were exclusive to haplotype A, but only 8 were exclusive to haplotype B (Supplementary Table S4). The remaining IDs were identified in both haplotypes (Supplementary Table S4).

### NLRs cluster into 2 distinct clades

**Figure 8:**
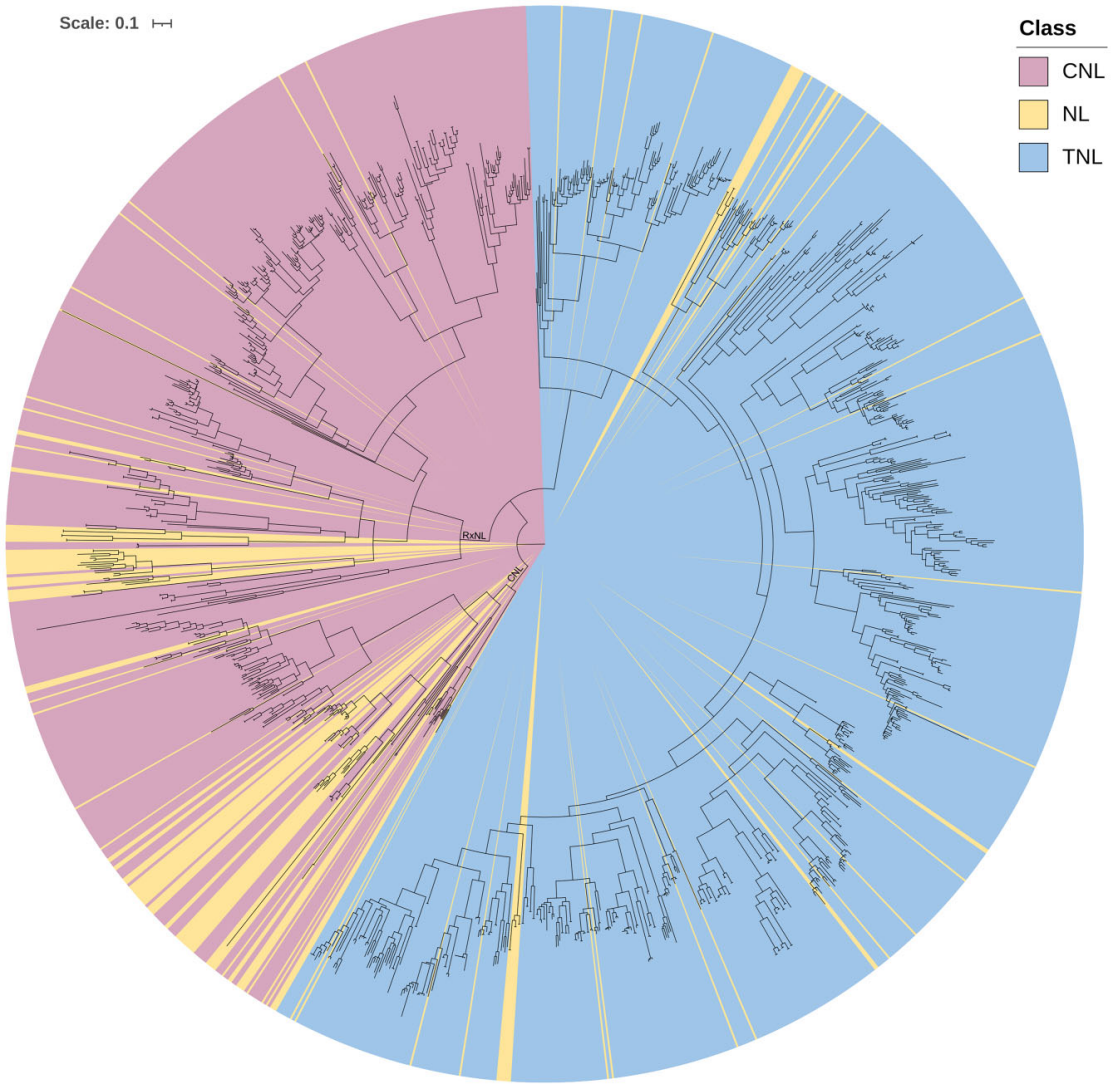
Evolutionary relationship of NBARC domains from predicted NLR genes within the phased *Melaleuca quinquenervia* genome. The NBARC domain fasta file and additional NBARC sequences, as out-groups, from functionally validated plant NLRs [34], were aligned with Clustal Omega (v1.2.4) [120]. The phylogenetic tree was inferred with the alignment file using IQ-TREE (v1.6.7) [121] and visualized in iTOL (v5) [122]. Each tip represents one putative *NLR* gene with branch lengths signifying rates of amino acid substitutions. Colors indicate the CNL (including RxNLs) (pink), TNL (blue), and NL (yellow) clades. Scale = 0.1 amino acid substitutions per site. The interactive tree can be viewed at https://itol.embl.de/shared/alyssamartino.

The evolutionary relatedness of the 1,328 NBARC domains (462 CNL, 726 TNL, and 140 NL) from complete NLR gene models separated into 2 major clades: CNL (CNL, RxNL, and RNL genes combined) and TNL genes (Fig. 8). Fifty-nine percent of all sequences aligned with the TNL (784) clade and 41 percent of total sequences aligned with the *CNL* clade (544), with 98 of the 140 NL sequences aligned with CNL and 42 aligned with TNL clades (Fig. 8). Fifteen *CNL* NBARC sequences clustered within the TNL clade, but no TNLs clustered within the CNL clade. On closer inspection of these 15 NBARC amino acid sequences, we determined that the integrity of the tree is correct due to the lack of the “W” (tryptophan) at the “LDD*W” kinase 2 subdomain (Supplementary Fig. S9). This is canonical for CNL clade NBARC domains but not present in the TNL clade [61]. We inspected the annotation and classification from FindPlantNLRs and found coiled–coil and Rx domains at the amino-terminus on these 15 gene models, hence the classification. It should be noted that all other NLR analyses in our study are based on the full annotated gene classification.

### Transcript evidence found for predicted NLRs

To confirm that *in silico* NLR predictions were actively expressed, we downloaded RNA sequencing (RNA-seq) data from a previous *M. quinquenervia* study that investigated responses to the plant pathogen causing myrtle rust [65]. We mapped all the available RNA-seq data to the NLR coding sequencing for each haploid genome independently using Hisat2 [66]. Taking the transcripts per million (TPM) cutoff of 50, we determined expression for 617 and 596 NLR coding sequences from haplotypes A and B, respectively. The most abundantly expressed predicted NLR gene is an *RPW8* (PF05659) *NLR* homologue, TPM 50,744 and 47,856 for haplotypes A and B, respectively. This gene is predicted on chromosome 6, NLR gene identifications, g7145.t1 and g1651.t1, respectively (Supplementary Table S3).

## Discussion

### A high-quality diploid genome for the keystone wetland species, *Melaleuca quinquenervia*

To promote scientific investigation, we have assembled a telomere-to-telomere diploid genome for a keystone wetland species, the broadleaved paperbark tree, *M. quinquenervia*. Using ∼70× HiFi coverage (35× per haplotype), combined with ∼380× Illumina Hi-C coverage, our assembly scaffolded into the expected 11 Myrtaceae chromosomes (2*n* = 22) and has a very high level of BUSCO completeness (Table 2). With careful curation to remove scaffolding errors and misassemblies, followed by polishing, we numbered 2 sets of parental chromosomes in accordance with the Myrtaceae reference genome, an inbred clone of *Eucalyptus grandis* [7]. We were able to show synteny between the *M. quinquenervia* chromosomes with 5 other publicly available chromosome-level Myrtaceae genomes (Fig. 2). Additionally, the genome and subsequent analyses were independently validated with scaffolded assemblies using ∼234× ONT data. Based on homology with 3 publicly available Myrtaceae proteomes and with *A. thaliana*, we predicted 28,744 and 28,517 protein-coding genes within the 2 chromosome sets. These numbers are slightly less per haplotype but comparable to the predicted 36,779 for the haploid genome of *E. grandis*. This is likely to be due to the earlier generation sequencing technology, assembly software, and the result of collapsed assemblies for highly heterozygous plants. We annotated repetitive genomic regions at ∼33% in both haplotypes, compared to 41% and 44% in *E. grandis* [7] and *E. pauciflora* [67], respectively, likely related to the smaller genome size for *M. quinquenervia*. There was a marked difference in rRNA content between the 2 haplotypes, and these differences are being driven by rRNA on unanchored contigs. Our curated assembly meets the high standards and metrics of the vertebrate genome project objectives [68], providing an exceptional resource for functional molecular and evolutionary studies.

### A smaller than predicted genome for *Melaleuca quinquenervia*

A 2C-value of 1.94 was previously reported in the literature using flow cytometry on samples from a tree in a university garden [68]. We therefore expected the genome size for each haploid assembly to be 949 Mb and planned our sequencing experiments accordingly. The *M. quinquenervia* genomes we assembled are much smaller, at ∼270 Mb, and polyploidy has not been reported in this species. The authors on the flow cytometry study reported problems processing their Myrtaceae samples, perhaps explaining the large size discrepancy in these results. To test that our results were accurate, we checked the ploidy and ran *k*-mer and read depth–based analyses, as described in the Methods. Results indicated the genome was 270 to 280 Mb, less than half the size of the *E. grandis* genome at 640 Mb [7]. While the genome size was surprising, we were able to use the high sequence coverage to ensure a highly accurate diploid genome.

### The annotated NLR complement for both *Melaleuca quinquenervia* chromosome sets

With the high quality of our genome, we were able to comprehensively annotate the NLR-type resistance genes in both inherited chromosome sets, using our novel FindPlantNLRs pipeline. Of the 1,495 annotated NBARC-containing genes identified in the *M. quinquenervia* genome (Fig. 5), we determined that 1,328 were complete NLRs while a further 167 contained the NBARC domain but lacked either, or both, the C- or N-terminal domains. The number of NBARC-containing genes in the genome is consistent with analysis of *E. grandis*, which was determined at 1,487 NBARC-containing genes [61] despite a much larger genome size. Although genome size is not directly correlated with NLR content [69], the presentation of the *E. grandis* genome in its collapsed form may result in underrepresentation of the NLRs as allelic variants. We estimated 125 genes in haplotype A had no ortholog in the alternate haplotype, while 107 from haplotype B had no ortholog in the alternate haplotype (Supplementary Figs. S6 and S7). To our knowledge, this is the first published research that has presented the allelic NLR complement in a phased, chromosome-level genome. As such, analysis of orthologs between haplotypes is limited to currently available software that is designed to compare species. The software limitation may therefore lead to some discrepancies in ortholog numbers within our analyses (Supplementary Figs. S6 and S7). Nonetheless, our detailed analysis highlights unique allelic variation that will assist research into the reported different phenotypic responses to pest- and pathogen-challenged species with the family Myrtaceae [65]. Our data might also be useful for understanding the strong evolutionary selection pressures on these plant immune receptors that has resulted in the allelic variation we present for *M. quinquenervia*. Analysis of gene families such as NLRs may also assist in understanding how invasive species manage to escape native-range microbes, as is the case for *M. quinquenervia* in Florida, where it is exposed to a new suite of microbes [70].

### *Melaleuca quinquenervia* NLRs are dominated by TNL-type resistance genes

Consistent with the *E. grandis* NLR annotation is the higher proportion of TNL- to CNL-type genes supporting an expansion of the TNL clade within the Myrtaceae [61]. This is further validated by recent phylogenetic analyses using transcripts from *M. quinquenervia* and *M. alternifolia* that revealed approximately two-thirds of NLR transcripts clustering with TNLs from *E. grandis* [71]. We found TNL to CNL ratios of ∼3:1 in haplotype A and ∼3:2 in haplotype B of *M. quinquenervia*. The ID-containing NLRs had a greater proportion of TNLs than CNLs with IDs (∼2:1 and 3:1 in haplotypes A and B, respectively). The TIR domain has been demonstrated to play a key role in the self-association of the NLR proteins to form higher-order resistosomes that are necessary for immune signaling [72]. Of particular interest of the TNL-type genes annotated are those containing a C-terminal jacalin domain and no LRR domain (Fig. 7). NLRs containing an alternative C-terminal domain have been identified in a range of agriculturally important plant species such as wheat, rice, sorghum, and barley, as well as tree species such as *E. grandis, Syzygium luehmannii*, and *M. quinquenervia* [61, 71, 73, 74]. Unlike conventional NLRs, which contain a C-terminal LRR domain, the LRR is replaced by a jacalin domain (PF01419), a mannose-binding lectin. Although previously thought of as a decoy domain for pathogen effectors, the replacement of the LRR domain by a jacalin domain suggests that this domain may play a role in NLR function. The expansion of the TIR class combined with fused IDs within TNLs, discussed later, may provide novel defense capacity against pests and pathogens. Chromosomal locations for all truncated NLRs are available in GigaDB [75].

### Phylogenetic evolutionary analysis supports the NLR classification results

By combining all the NBARC amino acid domains from both haplotypes, we visualized the evolutionary relatedness of NLRs. While the phylogenetic tree was based on alignment of NBARC domains and not full annotated genes, it demonstrated the clear divergence into CNL and TNL clades (Fig. 8) as observed in other plant species [33, 61]. Of the NLRs lacking CC or TIR domains (NLs), 42 are clustered in the TNL clade and the remaining 96 into the CNL clade. Of interest, the expansion of the TNL clade, also observed in *E. grandis* [61] with 53% TNL to 47% CNL, was comparable in *M. quinquenervia* with 59% TNL to 41% CNL (Fig. 8). There were 15 predicted CNLs that clustered within the TNL clade. On inspection of these amino acid sequences, we found that they had coiled–coil or Rx-type domains fused to classic TNL-type NBARC domains. Two of these NLRs have homologues in the alternative haplotype lacking an N-terminal domain, and one is homologous to a TNL gene. A further 5 have no homologous partner in the alternative haplotype, with the remaining 7 homologous to the NLRs with swapped domains. These results suggest amino terminal domain swapping as a possible evolutionary mechanism, but further functional and molecular validation is required.

### NLR physical clusters on chromosomes in *Melaleuca quinquenervia*

Analysis of the putative TNLs, CNLs, and NLs within the phased genome of *M. quinquenervia* revealed the majority of NLRs located within clusters, with 86% clustering in haplotype A and 88% in haplotype B. Only 14% and 12% from haplotypes A and B, respectively, did not fall into clusters, compared to approximately a quarter of NLRs in *E. grandis* [61], cultivated rice (*O. sativa*) [76], and *A. thaliana* [33], employing the same method for determining clusters. For *M. quinquenervia*, there were approximately 5 NLR genes for every Mb of the total genome size, while in *A. thaliana, E. grandis*, and *O. sativa*, the number of NLRs per Mb ranged from 1.2 to 2.3 [25, 61, 77]. The higher density of NLRs in the *M. quinquenervia* genome may explain the higher proportion of NLRs appearing in clusters. Closer inspection of NLR clusters revealed that some of the larger clusters overlapped with genome assembly gaps (Supplementary Figs. S6 and S7). As NLRs are highly repetitive, this may be the result of challenges associated with assembling highly repetitive genomic regions. This has been observed for other multicopy repetitive gene families such as the major histocompatibility complex family [78]. Nevertheless, the majority of NLRs are present at a read depth consistent with correct copy numbers (Fig. 3, Supplementary Figs. S4 and S5), indicating that assembly difficulties in NLR repeats has not substantially affected results.

Most clusters were homogeneous, containing NLRs of the same class, with only 4 heterogeneous clusters in haplotype A and 2 in haplotype B (Fig. 6C, D). The high proportion of homogeneous clusters suggests the expansion of these genes into clusters is driven by tandem duplication [79], as a mechanism for maintaining NLR diversity [80]. Clustering may also play an important role in pathogen resistance. NLR pairs such as *RGA4* and *RGA5* [81] and *Pik-1* and *Pik-2* in cultivated rice [82] are oriented in a head-to-head manner and function cooperatively in pathogen recognition and response, with one acting as a sensor of the pathogen and the other as an executor of immune signaling. This was also observed for the NLR pair *RPS4* and *RRS1* in *A. thaliana*, suggesting a shared promoter for the coregulation of the 2 genes [83, 84]. Interestingly, for each of these pairs, 1 partner from each contained an ID. On chromosome 3 of haplotype B of *M. quinquenervia*, 1 pair of NLRs was identified in this head-to-head manner, with 1 partner containing 1 RVT2 and 1 gag_pre-integrs ID. The identification of genes in the head-to-head manner in *M. quinquenervia* may indicate a functional role for these genes in disease resistance, with further studies needed to elucidate a potential function.

### The NLR repertoire is unique between haplotypes

Overall, the patterns of individual NLR numbers, classes, clusters, and cluster types across chromosomes appear consistent between the 2 haplotypes of *M. quinquenervia* (Figs. 5 and 6). However, analysis at the individual chromosome and gene level revealed diversity in the number and classes of genes between haplotypes for all except chromosomes 1 and 9 (Fig. 5). While consistent in gene number and gene number per class, analysis of the IDs across chromosome 1 revealed 1 gene on haplotype B to contain 2 DUF642 domains that were not present on the corresponding gene in haplotype A. Similarly, 1 gene in haplotype A of chromosome 9 contained 1 NAD_binding_11 and 1 NAD_binding_2 domains that were not present in the corresponding gene on haplotype B (Supplementary Table S3). The presence/absence of NLR polymorphisms between the haplotypes of *M. quinquenervia* are likely explained by the outcrossing nature of the species. High levels of genetic diversity maintained in long-lived, outcrossing woody species [85], combined with exposure to a range of pests and pathogens over their lifetime, may lead to changes in NLRs’ arrangement over subsequent generations. Presence/absence polymorphisms of NLRs have been observed in several plant species such as between inbred accessions of *O. sativa* and *A. thaliana* [86, 87]. This may be explained by the fitness cost associated with the maintenance of these genes [88], leading to loss of corresponding genes in the absence of the pathogen.

We identified a total of 53 unique IDs across both haplotypes, accounting for 4.4% of NLR genes in haplotype A and 6.8% in haplotype B. These figures are consistent with a recent review of published NLR-ID analyses that revealed 3.5% to 14% of NLRs contained IDs [27]. These fused integrated domains appear to mimic host proteins that are targets for pathogen effectors, leading to the triggering of defense response [26]. Some of the most commonly occurring integrated domains belong to families of proteins with critical roles in plant defense [26, 89] such as WRKY transcription factors and BED zinc fingers (BEAF and DREF from *Drosophila melanogaster* peptide; zf-BED). In the genome of *M. quinquenervia*, one of the most commonly occurring IDs was the WRKY domain, which was identified in 5 genes across the 2 haplotypes. A notable example of the role of an integrated WRKY domain present in an NLR is the *Arabidopsis Ralstonia solanacearum gene 1* (*RSS1-R*) [28, 90]. Bacterial effectors were found to bind to the WRKY domain of the NLR protein and other WRKY-containing proteins [90], suggesting a role for this domain as a decoy. Another common domain was the zf-BED domain, which was identified in 7 genes across the 2 haplotypes. While the function of the ID has yet to be elucidated, zf-BED domains have been observed in NLR genes conferring resistance to rust pathogens in barley, wheat, and rice [91–95]. The identification of these fused domains suggests a role for these genes in pathogen recognition.

### Potential implications

Long-lived tree species must respond to a wide range of biotic stresses. Our results provide insight into the diversity of the NLR gene family within a single-host tree species, indicating a potential mechanism for responses to invasive pathogens over a life span. We provide a framework for studying highly repetitive resistance genes by generating a high-quality pseudo-phased reference genome. With advances in sequencing and software, we are beginning to investigate the full repertoire of all genes, including NLRs, here starting with a representative Myrtaceae tree, *M. quinquenervia*. Given the diversity of NLRs from just 2 haplotypes, our results indicate that association studies of outcrossing species will need to model presence/absence of NLRs, in addition to segregating sequence variants. Future studies may expand to comparing population-level diversity of NLRs and the diversity of NLRomes across woody plants.

## Methods

### DNA extraction and sequencing

#### Sampling and DNA extraction

We obtained young fresh leaves (approximately 30 g) from a mature *M. quinquenervia* (Cav.) S.T. Blake tree growing at the RBG in Sydney, New South Wales (BioSample accession SAMN20854364), for use as the reference genome individual. We chose this specimen for the ease of ongoing access to leaf, cuttings, and seed material. The tree was planted in 1880 by HRH Prince George of Wales, later King George V. The tree is now 140 years old, of unknown provenance, and is showing signs of senescence.

For PacBio HiFi sequencing, we extracted high molecular weight (HMW) genomic DNA (gDNA) using 2 sorbitol washes [96] followed by a CTAB/NaCl/Proteinase K protocol [97]. We purified gDNA with 2 rounds of bead clean-up (AMPure Beads) and assessed resulting gDNA quality using Nanodrop2000 and Qubit 2.0 Fluorometer (dsDNA HS assay) to obtain a minimum ratio of 0.6.

For ONT Nanopore sequencing, we extracted HMW gDNA using a magnetic bead-based protocol described in [95]. We subsequently size selected the gDNA for fragments ≥40 kb using a PippinHT (Sage Science).

#### PacBio HiFi sequencing

We sent the final HMW gDNA sample of ∼100 μL, 451.7 ng/μL in 10 mM TrisHCl (∼45 μg HMW) to the Australian Genome Research Facility Ltd, St Lucia, Queensland, for HiFi 10- to 15-kb fragment gDNA Pippin Prep size selection, library preparation, and PacBio Sequel II sequencing (SMRT Cell 8 M).

#### Hi-C proximity-ligation sequencing

Hi-C library preparation and sequencing was conducted at the Ramaciotti Centre for Genomics using the Phase Genomics Plant kit v3.0. A pilot run on an Illumina iSeq 100 with 2 × 150–bp paired-end sequencing run was performed for quality control using hic_qc v1.0 (Phase Genomics, 2019) with i1 300 cycle chemistry. This was followed by sequencing on the Illumina NextSeq 500 with 2 × 150–bp paired-end high output run and NextSeq High Output 300 cycle kit v2.5 chemistry.

#### ONT Sequencing

We prepared a long-read native DNA sequencing library according to ONT protocol Genomic DNA by Ligation (SQK-LSK110). We performed sequencing on an ONT PromethION using a FLO-PRO002 R9.4.1 flow cell, with 3 wash treatments and reloads to maximize output, according to the manufacturer’s Flow Cell Wash Kit (EXP-WSH004). We basecalled the fast5 reads to fastq with Guppy basecaller (RRID:SCR_023196) v6.1.2 (model_version_id=2021–05-05_dna_r9.4.1_promethion_768_922a514b), inspecting the output and quality with NanoPlot [98].

#### Genome size prediction

We computed HiFi CCS read *k*-mer frequencies using Jellyfish v2.2.10 [99] and KMC v3.1.1 [100], with *k* = 19 and a maximum *k*-mer frequency of 10,000 (-k19 -ci1 -cs10000). We used the GenomeScope v2.0 webserver [49] to predict genome sizes.

We carried out additional genome size prediction using single-copy read depth analysis by DepthSizer v1.4.0 [48]. We mapped HiFi CCS and ONT reads to each genome assembly analyzed using minimap2 v2.22 [101] and calculated BAM depth and coverage statistics with SAMTOOLS (RRID:SCR_002105) v1.13 [102]. We used single-copy genes identified as “Complete” by BUSCO for each assembly. We generated genome size plots with the ggstatsplot package [103] in R v4.1.0.

#### Genome assembly and Hi-C scaffolding

We assembled the genome with the Hifiasm (RRID:SCR_021069) v0.15.5 [44] package using PacBio HiFi reads and integrating Hi-C reads. We independently scaffolded genome outputs using the Aiden Lab pipelines [45, 45] (assembly v0.1; Supplementary Fig. S3A, B). The assignment of scaffolds to either haplotype A or B was determined by hifiasm arbitrarily as the parent trees were not available to be sequenced. The ONT data were assembled with Flye (v2.9) [104], polished with Hypo (v1.0.3) [105], and scaffolded with Hi-C data (Supplementary Fig. S1C, D). To scaffold the genomes, we ran the Juicer pipeline (v1.6) [106] with default parameters. To ensure that all duplicate mapped reads were removed, we renamed the merged_sort.txt output from Juicer and reformatted and renamed the merged_nodups.txt to replicate the format of the original merged_sort.txt with the script “cat merged_nodups.txt |sort –parallel=16 -k2,2d -k6,6d > merged_sort.txt.” We reran Juicer using the newly created merged_sort.txt with additional parameter “-S dedup” and used the final output with the 3D-DNA pipeline (v180922) [46] with the following parameters: “-m haploid –build-gapped-map –sort-output.” After we manually curated the assemblies locally within the Juicebox visualization software (v1.11.08 for Windows) [45], we resubmitted the revised assembly file to the 3D-DNA postreview pipeline with the parameters “–build-gapped-map –sort-output” for final assembly and fasta files.

#### Assembly curation, filtering, and polishing

We tidied Hi-C scaffolds with Diploidocus (RRID:SCR_021231) (v0.18.0) [48] in dipcycle mode, using the HiFi reads for both long reads and high-accuracy (*k*-mer) reads (assembly v0.2) with each haplotype filtered independently. We assigned chromosomes with PAFScaff (v0.4.1) [107], mapping on to the *E. grandis* (GCF_000612305.1) chromosomes (assembly v0.3), and visually compared the 2 haplotypes, using SynBad (v0.8.4) [108] and DepthKopy (v1.1.0) [48] as guides. We identified some scaffolding errors, which we manually corrected (assembly v0.4) before a second round of Diploidocus tidy on each haplotype (assembly v0.5). We used DepthCharge (v0.2.0) [109] to assess for misassemblies, with none identified, but we failed to close any assembly gaps using LR Gapcloser (RRID:SCR_016194) (v20180904).

Next, we mapped the HiFi reads onto the diploid assembly with Minimap2 (RRID:SCR_018550) (v2.22) [101] and partitioned by haplotype. We separated nonchromosome scaffolds into contigs and ran a third round of Diploidocus tidy on each haplotype using the appropriate subset of haplotype-mapped HiFi reads (assembly v0.6).

We then polished the tidied diploid genome with HyPo (v1.0.3) [105] using the HiFi reads mapped with Minimap2 (v2.22) [101] for both the long-read and high-accuracy data (assembly v0.7). Finally, we renamed the chromosomes according to synteny with the *E. grandis* genome [7] to produce v1.0 of the *M. quinquenervia* genome.

#### Genome completeness, validation, and annotation

To determine genome completeness, we used BUSCO (v5.3.1) [53] using the lineage dataset embryophyta_odb10. Additionally, we estimated genome assembly quality (QV) using *k-*mer analysis of HiFi read data by Merqury (RRID:SCR_022964) v1.0 with *k* = 21 [52].

We used the homology-based gene prediction program GeMoMa (v1.7.1) [54] to annotate the genome, using 4 reference genomes downloaded from NCBI: *A. thaliana* (TAIR10.1, GCA_000001735.2), *E. grandis* [7] (GCF_000612305.1), *S. oleosum* (GCF_900635055.1), and *Rhodamnia argentea* (GCF_020921035.1). We predicted rRNA genes with Barrnap (v0.9) [110] and tRNAs with tRNAscan-SE (v2.05) [111], implementing Infernal (v1.1.2) [112] filtering for eukaryotes using the recommended protocol to form the high-confidence set. To generate a custom repeat library, we used RepeatModeler (v2.0.1) [55] following genome masking using RepeatMasker (RRID:SCR_012954) (v4.1.0) [113], both with default parameters. We generated the annotation table using the buildSummary.pl RepeatMasker script.

#### Synteny to other Myrtaceae

We used Chromsyn [47] to investigate synteny of *M. quinquenervia* to 5 chromosome-level Myrtaceae genomes available on NCBI: *Angophora floribunda* (GCA_014182895.1) [114], *E. grandis* [7] (GCF_016545825.1), *R. argentea* (GCF_020921035.1), *Psidium guajava* (GCA_016432845.1) [115], and *Syzygium aromaticum* (GCA_024500025.1) [116]. We ordered the species according to phylogenetic relationships [117].

### NLR analysis

#### NLR annotation with FindPlantNLRs

We developed a comprehensive pipeline to annotate predicted NLR genes from an unmasked genome fasta file input, named FindPlantNLRs [43]. The complete described protocol, including software version, dependencies, HMMs, and additional scripts, is available on GitHub [43].

#### Classification of annotated NLRs and identification of integrated domains

To identify all classes of annotated NLRs, we developed a script that sorted and classified the “gene” types. We ran the file outputs from FindPlantNLRs with the NLR classification script [43]. To further identify novel predicted integrated domains in the annotated NLRs, we developed a script to search the data based on Pfam domain identities not classically associated with NLRs [43]. Resulting files were then sorted to identify the predicted NLR genes by classification and integrated domains per phased genome. The formatted lists were then input to the web-based site sankeymatic.com/build/to create flow diagrams [64]. For all analyses downstream of the FindPlantNLRs pipeline, we included only full NLR gene models, which were defined as those genes containing both an NBARC domain and an LRR domain.

#### NLR cluster, duplicated gene, and ortholog analysis

Clustering analysis was based on previous analyses in *E. grandis* and *A. thaliana* genomes [61, 118]. We defined a cluster as a genomic region containing 3 or more predicted *NLR* genes, each of which had less than 250 kb from a *NLR* gene and with less than 8 non-*NLR* genes between each *NLR*.

We followed the *E. grandis* definition of class classification of *NLR* [61]. *CNL*-type clusters were defined by those containing at least 1 gene with a *CNL* domain and no *TNL*-type domains. *TNL*-type clusters were defined as those containing at least 1 gene with a *TNL* domain and no *CNL* domains. *NL* clusters were defined by those containing only genes with no N-terminal domains. Mixed-type clusters were defined as those containing at least 2 genes with differing N-terminal domains or lack of an N-terminal domain. We visualized the positions of individual *NLR*s and *NLR* clusters on *M. quinquenervia* chromosomes with ChromoMap [119] using base pair start and end positions.

We investigated genome-wide copy numbers using DepthKopy (v1.1.0) [48] for the HiFi and ONT assemblies, with analysis of the HiFi and ONT read data, examining the BUSCO genes, *NLR* annotations, *NBARC* regions, scaffolds, and 100-kb windows across the genome.

To identify orthologs, we aligned sister chromosomes of *M. quinquenervia* with minimap2 (2.24-r1122) [101] with -cx asm20, and alignments were filtered with “length ≥1000 bp and identity ≥90%.” We used GOPHER (v3.5.4) [120] to determine orthologs between haplotypes with default settings and used BEDTools (RRID:SCR_006646) intersect (v2.27.1) [121] to identify NLRs located in unaligned regions. Dot plots were generated with ggplot2 (v3.4.2) [122]. Syntenic graphs were generated with karyoploteR (RRID:SCR_021824) (1.26.0) [123] with nucleotide aligned regions from minimap2 (2.24-r1122) [101]. Gaps in the assembly were rated as either syntenic (both sides mapped in the correct order and orientation to the alternative haplotype) or nonsyntenic (mismatched best-matching scaffolds from the alternative haplotype for each side of the gap) using SynBad ratings [108].

#### Phylogenetic analysis of Melaleuca quinquenervia NLRs

To investigate relatedness among NLR genes, we extracted all NBARC domains from the annotated amino acid files for both sets of scaffolds using the chromosome locations with bedtools (v2.29.2) [120]. We included an out-group of amino acid NBARC domains taken from a subset of functionally validated plant NLRs [34]. We reduced the out-group set to include NBARC domains from eudicotyledons only and incorporated 6 CNL, 2 RPW8, and 7 TNL-type NBARC domains (Supplementary Table S5). We removed 81 predicted transcripts annotated as t2, retaining only t1 predicted reads, from the phased *M. quinquenervia* data and combined the remaining NLR NBARC domains with the out-groups. We aligned the combined sequences with Clustal Omega (v1.2.4) [124] and inferred the phylogenetic tree with IQ-TREE [125] using the following parameters: -bb 1000 -st AA -m LG. We visualized the resulting newick file with iTOL [126] and color-coded according to NLR clade.

To investigate the homologues of the 15 NLRs containing mismatched N-terminal and NBARC domains, we ran Proteinortho (RRID:SCR_024177) (v6.0.15) [127] on the NLRs used for phylogenetic analysis with BLASTP run using DIAMOND (RRID:SCR_009457) (v2.1.6) [128].

#### Transcript evidence for annotated NLRs in Melaleuca quinquenervia

To test for expression evidence for our annotated NLR genes, we downloaded RNA-seq data (NCBI PRJNA357284) from a previous *M. quinquenervia* study that investigated responses to the plant pathogen causing myrtle rust [65]. We mapped all the available RNA-seq data to the NLR coding sequences for each haploid genome independently using Hisat2 (v2.1.0) [66] with the parameters “hisat2 -p 16 –summary-file MqA/MqB –trim5 15 –trim3 10 –no-unal -p 16 -S <file.sam>.” We processed the SAM file outputs with samtools (v1.9) [102] for sorted and indexed BAM files and obtained mapping statistics with samtools idxstats. Finally, we calculated the TPM for all predicted NLR genes.

## Availability of Source Code and Requirements

Project name: FindPlantNLRs

Project homepage :https://github.com/ZhenyanLuo/FindPlantNLRs [43]

Operating system(s): Platform independent

Programming language: Python

Other requirements: none.

License: GPL 3.0

Any restrictions to use by nonacademics: none

RRID:SCR_02475

## Supplementary Material

giad102_GIGA-D-23-00119_Original_Submission

giad102_GIGA-D-23-00119_Revision_1

giad102_GIGA-D-23-00119_Revision_2

giad102_Response_to_Reviewer_Comments_Original_Submission

giad102_Response_to_Reviewer_Comments_Revision_1

giad102_Reviewer_1_Report_Original_Submission Andrew Read -- 6/14/2023 Reviewed

giad102_Reviewer_1_Report_Revision_1 Andrew Read -- 9/19/2023 Reviewed

giad102_Reviewer_2_Report_Original_Submission Julia Voelker, Ph.D. -- 6/20/2023 Reviewed

giad102_Supplemental_File

## Data Availability

The resistance gene annotation tool is available at https://github.com/ZhenyanLuo/FindPlantNLRs [43] and is registered on bio.tools (https://bio.tools/findplantnlrs). The genome assemblies and raw sequencing data are available on NCBI under the Umbrella BioProject PRJNA756045, which is linked to the HapA assembly and the raw data used to generate both haplotypes; the HapB assembly was deposited to BioProject PRJNA911843. Other data further supporting this work are openly available in the *GigaScience* repository, GigaDB [75].

## Abbreviations

bp: base pair; BUSCO: Benchmarking Universal Single-Copy Orthologs; CC: coiled–coil; CN: coiled–coil nucleotide binding; CNL: coiled–coil nucleotide binding leucine-rich repeat; Gb: gigabase; HMM: hidden Markov model; HR: hypersensitive response; ID: integrated domain; LRR: leucine-rich repeat; Mb: megabase; NB: nucleotide binding; NBARC: nucleotide binding Apaf-1, R-protein, and CED-4; NL/NLR: nucleotide binding leucine-rich repeat; ONT: Oxford Nanopore Technologies; RBG: Royal Botanic Gardens; RNA-seq: RNA sequencing; RPW8/CC-R: RESISTANCE TO POWDERY MILDEW 8–like coiled–coil; rRNA: ribosomal RNA; RxNL: potato CC-NB-LRR protein Rx nucleotide binding leucine-rich repeat; TIR: Toll/interleukin 1 receptor/resistance protein; TN: Toll/interleukin 1 receptor/resistance nucleotide binding; TNL: Toll/interleukin 1 receptor/resistance nucleotide binding leucine-rich repeat; TPM: transcripts per million; tRNA: transfer RNA.

## Supplementary Data

**Supplementary Figure S1**. Hi-C contact maps from Juicebox for HiFi genomes Haplotype A (A) and (B) and ONT genomes Haplotype A (C) and B (D).

**Supplementary Figure S2**. Visualisation of genome assembly and curation steps for Melaleuca quinquenervia using ChromSyn for (A) Haplotype A or 1 and (B) Haplotype B or 2. Synteny blocks of collinear “Complete” BUSCO genes link scaffolds from adjacent assemblies: blue, same strand; red, inverse strand. Yellow triangles mark “Duplicated” BUSCOs. Filled circles mark telomere predictions from Diploidocus (black) and TIDK (blue). Assembly gaps are marked as dark red + signs. Assignment of ONT haplotypes was independent of assignment of HiFi haplotypes.

**Supplementary Figure S3**. Kmer analysis with GenomeScope (A) and ploidy analysis with Smudgeplot (B).

**Supplementary Figure S4**. Genome-wide regional copy number analysis for Melaleuca quinquenervia HiFi genomes using ONT read data (A-B). Analysis of ONT genomes with HiFi read data (C-D) and ONT read data (E-F). Haplotype A is presented in the left column and Haplotype B is presented in the right column. Copy number (CN) is relative to a single diploid (2n) copy in the genome. Violin plots and means generated with ggstatsplot. Each data point represents a different genomic region: BUSCO, BUSCO v5 (MetaEuk) single-copy “Complete” genes; Duplicated, BUSCO v5 “Duplicated” genes; NLR, resistance gene annotations; NBARC, NBARC domains; Sequences, assembly scaffolds; and Windows, 100 kb nonoverlapping windows across the genome. Plot truncated at CN = 4.

**Supplementary Figure S5**. Independent validation of NLR annotation visualised using ChromSyn using ONT genomes. Synteny blocks of collinear “Complete” BUSCO genes link scaffolds from adjacent assemblies: blue, same strand; red, inverse strand. Triangles mark “Duplicated” BUSCOs and diamonds mark NLRs, with colour denoting DepthKopy predicted copy number (CN: 0, grey; <0.33, yellow; <0.75, light blue; <1.5, blue; <2.5, pink: 2.5+, red). Filled circles mark telomere predictions from Diploidocus (black) and TIDK (blue). Assembly gaps are marked as dark red + signs.

**Supplementary Figure S6**. Synteny plots of NLR genes. Blue lines represent syntenic gaps identified by synbad, red lines represent non-syntenic gaps, grey shades represent homologous regions between sister chromosomes, brown lines link orthologs, genes are labelled with different colours based on class.

**Supplementary Figure S7**. Synteny plots of NLR gene clusters on the two haplotypes. Blue lines represent syntenic gaps identified by synbad, red lines represent non-syntenic gaps, grey shades represent homologous regions between sister chromosomes, blue lines link orthologs. NLR genes are labelled with different colours based on class.

**Supplementary Figure S8**. Whole genome dot plots depicting the alignment of homologous sister chromosomes. Red lines represent homologous regions between the compared chromosomes, grey lines represent the location of NLRs on both chromosome.

**Supplementary Figure S9**. Alignment of the NBARC region of 15 NBARC containing genes from Melaleuca quinquenervia aligned with Eucalyptus grandis NBARC regions used to resolve the class clasifcation genes in the phylogeny. The “W” tryptophan at the ‘LDD*W’ at ~110 aa is present only for the CNL class genes, while the 15 M. quinquenervia genes follow the pattern of TNL class NBARCs.

**Supplementary Table S1**. DepthSizer genome size estimation output.

**Supplementary Table S2**. Summary of numbers and classes of NBARC contianing genes within the genome of Melaleuca quinquenervia identified using the FindPlantNLRs pipeline.

**Supplementary Table S3**. Full list of NLR genes within the Melaleuca quinquenervia genome identified using the FindPlantNLRs pipeline.

**Supplementary Table S4**. Complete list of integrated domains identified in the Melaleuca quinquenervia genome along with their orthologs in the alernative haplotype determined using ProteinOrtho.

**Supplementary Table S5**. The outgroup of amino acid NBARC domains were selected from functionally validated plant NLRs (Kourelis et al. 2021). The set was reduced to include NBARC domains from eudicotyledons only and incorporated six CNL, two RPW8 and seven TNL-type NBARC domains.
