# Supplementary material for: A high-quality pseudo-phased genome for Melaleuca quinquenervia shows allelic diversity of NLR-type resistance genes
Source: Gigascience. 2023 Dec 14;12:giad102. doi: 10.1093/gigascience/giad102 (PMC10720953; doi:10.1093/gigascience/giad102)
Supplement: giad102_GIGA-D-23-00119_Revision_2 [file giad102_giga-d-23-00119_revision_2.pdf]

GigaScience

A high-quality pseudo-phased genome for Melaleuca quinquenervia shows allelic diversity of NLR-type resistance genes

--Manuscript Draft--

**Manuscript Number:**	GIGA-D-23-00119R2
**Full Title:**	A high-quality pseudo-phased genome for Melaleuca quinquenervia shows allelic diversity of NLR-type resistance genes
**Article Type:**	Research
**Funding Information:**	Australian Research Council (LP190100093) — Associate Professor Benjamin Schwessinger; Dr Peri A Tobias<br>Australian Research Council (LP18010072) — Dr Jason G Bragg; Dr Richard J Edwards
**Abstract:**	Background<br><br>The coastal wetland tree species Melaleuca quinquenervia (Cav.) S.T.Blake (Myrtaceae), commonly named the broad-leaved paperbark, is a foundation species in eastern Australia, Indonesia, Papua New Guinea, and New Caledonia. The species has been widely grown as an ornamental, becoming invasive in areas such as Florida in the United States. Long-lived trees must respond to a wide range pests and pathogens throughout their lifespan, and immune receptors encoded by the nucleotidebinding domain and leucine-rich repeat containing (NLR) gene family play a key role in plant stress responses. Expansion of this gene family is driven largely by tandem duplication, resulting in a clustering arrangement on chromosomes. Due to this clustering and their highly repetitive domain structure, comprehensive annotation of NLR encoding genes within genomes has been difficult. Additionally, as many genomes are still presented in their haploid, collapsed state, the full allelic diversity of the NLR gene family has not been widely published for outcrossing tree species.<br><br>Results<br><br>We assembled a chromosome-level pseudo-phased genome for M. quinquenervia and describe the full allelic diversity of plant NLRs using the novel FindPlantNLRs pipeline. Analysis reveals variation in the number of NLR genes on each haplotype, differences in clusters and in the types and numbers of novel integrated domains.<br><br>Conclusions<br><br>We anticipate that the high quality of the genome for M. quinquenervia will provide a new framework for functional and evolutionary studies into this important tree species. Our results indicate a likely role for maintenance of NLR allelic diversity to enable response to environmental stress, and we suggest that this allelic diversity may be even more important for long-lived plants.
**Corresponding Author:**	Richard J Edwards<br>The University of Western Australia<br>Perth, WA AUSTRALIA
**Corresponding Author Secondary Information:**	
**Corresponding Author's Institution:**	The University of Western Australia
**Corresponding Author's Secondary Institution:**	
**First Author:**	Stephanie H Chen
**First Author Secondary Information:**	

Powered by Editorial Manager® and ProduXion Manager® from Aries Systems Corporation

**Order of Authors:**	Stephanie H Chen
	Alyssa Marie Martino
	Zhenyan Luo
	Benjamin Schwessinger
	Ashley Jones
	Tamene Tolessa
	Jason G Bragg
	Peri A Tobias
	Richard J Edwards
**Order of Authors Secondary Information:**	
**Response to Reviewers:**	Please see attached covering letter for a formatted version.<br><br>Dear Dr Nogoy<br>RE: Response to Reviewer Comments - GIGA-D-23-00119<br><br>Thank you for the opportunity to submit an updated revision to our manuscript, "A high-quality pseudo-phased genome for Melaleuca quinquenervia shows allelic diversity of NLR-type resistance genes" (GIGA-D-23-00119). We appreciate the attention to detail of the reviews and have made the requested revisions. These are listed in detail, below. The updated manuscript has been uploaded in two versions: with and without changes highlighted in red.<br><br>Yours sincerely,<br><br>Dr. Richard Edwards<br>Laboratory Lead, Ocean Genomes Laboratory<br>Minderoo OceanOmics Centre at UWA<br>UWA Oceans Institute<br>Adjunct Associate Professor in Genomics and Bioinformatics<br>School of Biotechnology and Biomolecular Sciences<br>UNSW Sydney<br><br><br><br><br>Reviewer #1<br>Thank you for sharing a revised version of the manuscript - it's clear that a lot of work was put into making the changes. I believe that most of my concerns were addressed and am confident that my additional comments can be addressed without another round of review.<br>Main Text:<br>Specific<br>236 - "NLRs require NB and LRR to be functional" - this is not true, the variation observed in NLR modules is described in Monteiro and Nishimura Annual Reviews 2018.<br>A solution could be to slightly change to say "canonical NLRs require…"<br>Added “canonical” as suggested.<br><br>248 - please replace "genes" with "NLR genes" to avoid confusion<br>Added “NLR” as suggested.<br><br>295 - delete "novel" - did you check to see if any of your predicted domain coordinates overlap each other? I've seen this in the past and it can lead to artificially high counts. Consider removing the percentages to avoid misleading (unless you did check).<br>Novel has been removed and for clarity the percentages changed to now reflect those NLRs containing more than one unique integrated domain.<br><br>398 - I would consider replacing "no natural enemies" with "where it is exposed to a

	new suite of microbes" or similar.<br>Replaced no natural enemies with the suggested text.<br><br>417 - I think a more general statement here is appropriate - "this domain may play a role in NLR function"<br>Made statement more general as suggested.<br><br>431 - I appreciate the comment about full-length NLRs, but it seems out of place here. I still think it belongs in "Novel pipeline to identify and classify NLRs" section of the paper.<br>We have removed the comment of the full-length NLRs from line 412 and added the comment in lines 221 – 223.<br><br>Figures:<br><br>General<br>The figures that use a unified color palette for haplotype A and B are easier to interpret - consider extending this to all figures. For example, Figure 2 and Figure 6 use different colors than the oranges used in other figures.<br>The colours used for Hap A and B in Figure 2 have been changed to match the colours used in other figures. The palette used for the other species in the synteny plot have also been updated to complement and be easy to distinguish from the Hap A and B colours.<br>Figure 6 has been amended to reflect the colours used throughout the other figures for consistency.<br><br>Figures need more informative axes labels.<br>Axes labels have been updated for Figures 5 – 7 for clarity.<br><br>Specific<br>Figure 2 - adding the full species name would help readability, especially for those not intimately familiar with these species<br>The full scientific name for each species has been added to the figure.<br><br>Figure 4 - it would be helpful to add a label stating how much sequence is added to the NLR loci (20kb)<br>This has been added to the figure.<br><br>Figure 5 and 6 - Consider merging C and D so that all data hapA and B for each chromosome are grouped - the important comparison here seems to be 'how are the counts on chromosomes different across the two haplotypes'.<br>While I agree part of the focus is the differences between haplotypes, our preference would be to keep these figures as they stand. Merging the graph into differences between haplotypes may reduce the visual representation of the large number of the NLRs that exist within the M. quinquenervia genome.<br><br>Figure 6 legend - "D and E" referenced in legend, no D and E in current iteration of figure<br>This has been corrected.<br><br>Figure 7 legend - please replace "(Ref)" with appropriate reference. Add to legend that the count of each domain is included. *I really like this figure<br>Reference added and legend amended to include details of the count of each domain is included. Thank you, this is one of our favourite figures too!
**Additional Information:**	
**Question**	**Response**
Are you submitting this manuscript to a special series or article collection?	No
**Experimental design and statistics**	Yes

Powered by Editorial Manager® and ProduXion Manager® from Aries Systems Corporation

Full details of the experimental design and statistical methods used should be given in the Methods section, as detailed in our Minimum Standards Reporting Checklist. Information essential to interpreting the data presented should be made available in the figure legends.<br><br>Have you included all the information requested in your manuscript?	
**Resources**<br><br>A description of all resources used, including antibodies, cell lines, animals and software tools, with enough information to allow them to be uniquely identified, should be included in the Methods section. Authors are strongly encouraged to cite Research Resource Identifiers (RRIDs) for antibodies, model organisms and tools, where possible.<br><br>Have you included the information requested as detailed in our Minimum Standards Reporting Checklist?	Yes
**Availability of data and materials**<br><br>All datasets and code on which the conclusions of the paper rely must be either included in your submission or deposited in publicly available repositories (where available and ethically appropriate), referencing such data using a unique identifier in the references and in the "Availability of Data and Materials" section of your manuscript.<br><br>Have you have met the above requirement as detailed in our Minimum Standards Reporting Checklist?	Yes

Powered by Editorial Manager® and ProduXion Manager® from Aries Systems Corporation

Manuscript w. DOI

Click here to access/download;Manuscript;GIGA-D-23-00119-Revision2 w. doi.docx

A high-quality pseudo-phased genome for *Melaleuca quinquenervia* shows allelic diversity of NLR-type resistance genes

***Joint first authors**

Stephanie H Chen*, stephanie.h.chen@unsw.edu.au, School of Biotechnology and Biomolecular Sciences, UNSW Sydney, Kensington NSW 2052, Australia; Research Centre for Ecosystem Resilience, Botanic Gardens of Sydney, Sydney NSW 2000, Australia

Alyssa M Martino*, alyssa.martino@sydney.edu.au, School of Life and Environmental Sciences, The University of Sydney, Camperdown NSW 2006, Australia

Zhenyan Luo, zhenyan.luo@anu.edu.au, Research School of Biology, The Australian National University, Canberra ACT 2601, Australia

Benjamin Schwessinger, benjamin.schwessinger@anu.edu.au, Research School of Biology, The Australian National University, Canberra ACT 2601, Australia

Ashley Jones, ashley.jones@anu.edu.au, Research School of Biology, The Australian National University, Canberra ACT 2601, Australia

Tamene Tolessa, ttolessa@myune.edu.au, Research School of Biology, The Australian National University, Canberra ACT 2601, Australia; School of Environment and Rural Science, University of New England, Armidale NSW 2351, Australia

^ Joint corresponding authors

Jason G Bragg^, jason.bragg@botanicgardens.nsw.gov.au, Research Centre for Ecosystem Resilience, Botanic Gardens of Sydney, Sydney NSW 2000, Australia; School of Biological, Earth and Environmental Sciences, UNSW Sydney, Kensington NSW 2052, Australia

Peri A Tobias^, peri.tobias@sydney.edu.au, School of Life and Environmental Sciences, The University of Sydney, Camperdown NSW 2006, Australia

Richard J Edwards^, rich.edwards@uwa.edu.au, Minderoo OceanOmics Centre at UWA, UWA Oceans Institute, University of Western Australia, Crawley WA 6009, Australia; School of Biotechnology and Biomolecular Sciences, UNSW Sydney, Kensington NSW 2052, Australia

Abstract

Background

Melaleuca quinquenervia (broad-leaved paperbark) is a coastal wetland tree species that serves as a foundation species in eastern Australia, Indonesia, Papua New Guinea, and New Caledonia. While extensively cultivated for its ornamental value, it has also become invasive in regions like Florida, United States. Long-lived trees face diverse pest and pathogen pressures, and plant stress responses rely on immune receptors encoded by the nucleotide-binding leucine-rich repeat (NLR) gene family. However, the comprehensive annotation of NLR encoding genes has been challenging due to their clustering arrangement on chromosomes and highly repetitive domain structure; expansion of the NLR gene family is driven largely by tandem duplication. Additionally, the allelic diversity of the NLR gene family remains largely unexplored in outcrossing tree species, as many genomes are presented in their haploid, collapsed state.

Results

We assembled a chromosome-level pseudo-phased genome for *M. quinquenervia* and described the allelic diversity of plant NLRs using the novel FindPlantNLRs pipeline. Analysis reveals variation in the number of NLR genes on each haplotype, distinct clustering patterns, and differences in the types and numbers of novel integrated domains.

Conclusions

The high-quality *M. quinquenervia* genome assembly establishes a new framework for functional and evolutionary studies of this significant tree species. Our findings suggest that maintaining allelic diversity within the NLR gene family is crucial for enabling responses to environmental stress, particularly in long-lived plants.

Keywords

NLR, resistance genes, *Melaleuca quinquenervia* genome, FindPlantNLRs, broad-leaved paperbark

Background

Melaleuca quinquenervia (Cav.) S.T. Blake [1] is a broad-leaved paperbark tree endemic to the wetlands of eastern Australia, Papua New Guinea, New Caledonia and Indonesia (Figure 1) [2]. *Melaleuca quinquenervia* belongs to the family Myrtaceae, a large family of woody flowering plants consisting of over 144 genera and 5,500 species [3] with the genus *Melaleuca* comprising almost 300 species [2]. While *M. quinquenervia* is keystone species in its native range, it is planted extensively as an ornamental and is commercially important as a source of essential oils and nectar for honey [2]. The species has become highly invasive in the wetlands of Florida in the United States following its introduction as an ornamental in the early 1900s [4] and has increased fire risk and caused the significant loss of native vegetation and associated biodiversity in wetland areas [5]. The management of *M. quinquenervia* outside its native range has a serious economic impact due to labour intensive management practices including site monitoring, the physical removal of trees, and herbicide application [4]. High accuracy reference genomes are important for molecular and evolutionary studies, as well as providing a tool for strategic management of native and invasive species. With no current genome resource for *M. quinquenervia*, molecular research has been limited to homology-based studies using plants within the Myrtaceae family, including the closely-related species *Melaleuca alternifolia* [6–8].

Figure 1. Global distribution of *Melaleuca quinquenervia* in its native range (Australia, Papua New Guinea, New Caledonia and Indonesia; pink dots) and introduced range (blue dots). Data sourced from GBIF with darker shades indicative of higher record densities. Map generated using OpenStreetMap, licensed under the Open Data Commons Open Database License. Photos of the genome tree and detail of bark used in map background taken in the Royal Botanic Garden Sydney by SH Chen and PA Tobias.

Long living tree species, such as *M. quinquenervia*, are exposed to extensive biotic stresses over their lifetime [9], including a wide range of pests and pathogens. Plants employ various strategies to combat pests and pathogens. These include preformed physical barriers such as leaf cuticles [10,11] and changes in leaf anatomy [12], and chemical barriers such as secondary metabolites [13,14]. At a molecular level, plants rely on an innate immune system to recognise and respond to pathogens [15]. The plant immune system can be considered as two distinctly activated, but interplaying pathways involving cross talk between pathogen and host [16]. Research has therefore focussed on understanding the molecular basis of host tree responses to inform management, with a key emphasis on recognition and response to invasion patterns [17].

There has been substantial research focused on understanding the rapid, cascading response leading to programmed cell death, initiated by resistance receptors of the Nucleotide-binding Leucine-rich

Repeat (NLR) domain-type [18]. The genes encoding NLRs are a large group of plant resistance genes and are modular in their structure, generally containing three main domains: a nucleotide binding (NB) domain, an N-terminal domain, and a C-terminal domain. The NB site, or NBARC (Apaf-1, R-protein and CED-4) is highly conserved in plants, having an important role in activation of the hypersensitive response (HR) which blocks disease progression by stimulating programmed cell death within and around the infected region [19]. Of the 8 motifs constituting the NBARC, the P-loop motif is the most highly conserved, being essential for ATP hydrolysis and NLR function [20]. The NLR N-terminal domain is commonly a Toll/Interleukin-1 receptor/ Resistance protein (TIR) domain, a coiled-coil (CC) domain, or a RESISTANCE TO POWDERY MILDEW 8-like coiled-coil (RPW8/CC-R) domain [21]. Studies have demonstrated an important role for this domain for pathogen recognition and signalling [22,23]. Plant NLRs also contain leucine rich repeats (LRRs) which are subject to strong diversifying selection and show high sequence diversity even within closely related genes [24]. Studies suggest the high diversity of this region is the result of co-evolution between host and pathogen with several studies showing specific pathogen ligand interaction at this site.

While NLRs share common domains, they are highly diverse, even within the well-studied model species *Arabidopsis thaliana* [25]. Adding to this diversity is the addition of novel integrated domains (IDs) which can be numerous within a NLR protein and are located at various locations within the modular structure of these proteins [26]. Mimicking host proteins, evidence suggests that these domains function as decoy targets for pathogen secreted molecules, known as effectors, allowing for host recognition and triggering immune signalling [27]. A well-documented example is the RRS1 NLR in *A. thaliana* which carries a WRKY domain [28]. It interacts with RPS4 to recognise effectors from a range of pathogens, with the pair forming a complex that is activated upon targeting/modification of the WRKY domain [28]. Without this recognition, pathogen effectors were found to inhibit host WRKY DNA-binding that plays a role in defence signalling, indicating a role for the ID as a decoy [28]. Other notable examples include RGA5 and Pik-1 in rice which both contain a heavy metal associated domain that recognise effectors from the rice blast pathogen *Magnaporthe oryzae* [29,30].

NLR genes are also known to be numerous in many plant genomes [31], representing over 2% of all genes in apple (*Malus domestica*) [32]. While initial studies computationally identified 149 putative NLR-type genes in the genome of *A. thaliana* [33], more recently, a core set of 106 NLR orthogroups (6,080 genes) has been established across 52 plant accessions largely found in Europe [25] showing the incredible diversity of these genes within a single species. Despite the importance of this gene family in determining plant disease resistance, only 481 genes from 31 species have been fully or partially functionally characterised [34].

Overcoming the challenges associated with assembling these highly polymorphic and repetitive genes has been aided by sequencing technologies such as Oxford Nanopore Technologies (ONT) and PacBio HiFi [35,36]. By facilitating the generation of more contiguous genome assemblies, these technologies allow for greater characterisation of, and evolutionary analysis of NLR genes. This was highlighted in recent analysis of an updated reference genome of barley [37] which revealed over double the number of NLR genes compared to previous assemblies generated with short reads [38,39]. It has also aided in the generation of a near complete NLRome in *A. thaliana,* allowing for the mapping of NLR genes which were previously uncharacterised [25].

The genomes of many diploid organisms are represented as collapsed consensus sequences from homologous chromosomes [40]. Owing to the highly repetitive nature of plant NLRs*,* detailed genome wide analysis of NLR allelic variation is yet to be carried out. Studies have revealed extensive allelic variation in *NLR* genes such as eight brown planthopper resistance genes in *Oryza sativa* [40]. These results indicate the importance of detailed analysis of both chromosome sets to more accurately characterise NLRs, with the outcomes having implications for plant:pathogen coevolution and informing downstream molecular analyses. Recent developments in sequencing and scaffolding methods [41] provides the opportunity to generate phased genomes of highly heterozygous organisms such as *M. quinquenervia* [6,42].

Here we present a chromosome-level and pseudo-phased diploid genome assembly for *M. quinquenervia.* We make available FindPlantNLRs [43], a novel pipeline to fully annotate putative NLR genes, taking a genome file as the starting point (Figure 2). We compare NLR allelic variance within the phased, chromosome-level genome assembly of *M. quinquenervia* to provide the first example, to our knowledge, of NLR diversity in a diploid tree genome. Our data indicates that copy number, presence/absence and integrated domains are highly variable between haplotypes. These findings reveal the high level of diversity that exists for NLRs within a single plant genome. With much of this lost in a collapsed form, we demonstrate the importance of our approach to assist research into plant responses to environmental challenges.

Analyses

A high quality pseudo-phased genome assembly for Melaleuca quinquenervia

We sourced leaf material from a mature *M. quinquenervia* tree growing at the Royal Botanic Garden (RBG) Sydney, New South Wales, for use as the reference genome. The tree was planted in 1880, is 140 years old, of unknown provenance, and is a vouchered specimen of the RBG living collections. High molecular weight DNA was extracted for PacBio HiFi and ONT sequencing. Fresh leaf samples were sent for Hi-C library preparation and sequencing. We assembled the *M. quinquenervia* genome with HiFiasm [44] using HiFi sequencing data and integrating Hi-C data, with a total yield of 19.46 Gb and 116.4 Gb reads respectively (Table 1). We independently scaffolded the resulting pseudo-phased outputs using the Aidan Lab pipelines [45–47] and determined each haplotype comprised of 11 chromosomes with 94% of sequences assigned to chromosomes for both haplotypes (Figure S1A and B). To independently verify the HiFi assemblies, we assembled and scaffolded the ONT data (Figure S1C and D) which showed a high degree of synteny to the HiFi assemblies (Figure S2A and B). Our final assembly genomes were 269,244,392 bp and 271,680,404 bp for Haplotype A and B respectively (Table 2). We used Chromsyn [48] to investigate synteny of *M. quinquenervia* to five chromosome-level Myrtaceae genomes, all with $2n = 22$ chromosomes (Figure 2). The scaffolding of Haplotype A is supported by the scaffolding of Haplotype B for *M. quinquenervia*, despite the processes being run independently. We determined some inversions against the other Myrtaceae genome chromosomes that likely represent misassemblies in the less contiguous assemblies (Figure 2).

We checked the genome outputs using Depthsizer [49] using HiFi and ONT reads to show a genome size of approx. 274 Mb and 272 Mb for Haplotype A and B, respectively, with the ONT assembly giving similar figures (Table S1). We further validated the genome size using GenomeScope [50] which predicted a haploid genome size of 262 Mb (Figure S3A). We confirmed the diploid state of the genome using SmudgePlot [51] (Figure S3B).

To improve the overall quality of the *M. quinquenervia* genomes, we carried out several rounds of scaffolding, polishing and gap filling, with telomeres predicted by both Diploidocus [49] and tidk [52] at the end of chromosome scaffolds in most instances (Figure S2A and B). There are only a small number of gaps (fewer than 60) (Figure S2A and B).

Base pair level accuracy was tested against Merqury [53] with both haplotypes showing very high quality and accuracy scores. Additionally, we determined very high genome completeness of both haplotypes using Benchmarking Universal Single Copy Orthologs (BUSCO) [54] (Table 2, Figure 3A and B, Figure S4A-F). We ran GeMoMa [55] annotation on the two haplotypes and both proteomes were

99.7% complete according to BUSCO. We assessed the repetitive, as well and transfer (tRNA) and ribosomal RNA (rRNA) elements using RepeatModeler [56] (Table 2).

Table 1. Genomic sequence reads for the *Melaleuca quinquenervia* genome.

Sequencing platform	Library	Median insert size (bp)	Mean read length (bp)	No. of reads	Sequence bases (Gb)
PacBio Sequel II	HiFi SMRTbell	16,506	17,058	1,140,849	19.46
Illumina NextSeq 500[‡]	Phase Genomics Proximo Hi-C (Plant)	-	2 x 151	770,901,164	116.4
Oxford Nanopore Technologies	Ligation (SQK-LSK110)	-	26,803	2,400,431	64.68
**Total gDNA**	**-**	**-**	**-**	**774,442,444**	**200.5**

[‡] Includes a pilot iSeq run used to QC the library

Figure 2. Synteny between *Melaleuca quinquenervia* phased genome and selected chromosome-level Myrtaceae genomes (*Angophora floribunda*, *Eucalyptus grandis, Rhodamnia argentea*, *Psidium guajava* and *Syzygium aromaticum*). Synteny blocks of collinear "Complete" BUSCO genes link scaffolds from adjacent assemblies: blue, same strand; red, inverse strand. Yellow triangles mark "Duplicated" BUSCOs. Filled circles mark telomere predictions from Diploidocus (black) and tidk (blue). Assembly gaps are marked as dark red + signs.

Figure 3. Genome-wide regional copy number analysis for *Melaleuca quinquenervia* (A) Haplotype A and (B) Haplotype B using HiFi read data. Copy number (CN) is relative to a single diploid (2*n*) copy in the genome. Violin plots and means generated with ggstatsplot. Each data point represents a different genomic region: BUSCO, BUSCO v5 (MetaEuk) single-copy "Complete" genes; Duplicated, BUSCO v5 "Duplicated" genes; NLR, resistance gene annotations; NBARC, NBARC domains; Sequences, assembly scaffolds; and Windows, 100 kb non-overlapping windows across the genome. Plot truncated at CN = 4.

Table 2. Genome statistics for the *Melaleuca quinquenervia* phased reference genome.

Statistic	Haplotype A	Haplotype B
**Total length (bp)**	269,244,392	271,680,404
**No. of scaffolds**	196	183
N50 (bp)†	22,766,892	22,112,861
L50‡	6	6
**No. of contigs**	251	241
N50 (bp)†	7,525,323	5,650,000
L50‡	14	16
No. of gaps	55	58
GC (%)	40.38	40.51
**BUSCO complete (genome; *n* = 1,614)**	**99.1% (1,599)**	**98.8% (1,595)**
Single-copy (genome)	98.0% (1,581)	97.7% (1,577)
Duplicated (genome)	1.1% (18)	1.1 % (18)
BUSCO fragmented (genome)	0.6% (9)	0.7% (12)
BUSCO missing (genome)	0.3 % (6)	0.5 % (7)
**Protein-coding genes (GeMoMa)**	28,744	28,517
mRNAs	43,219	42,866
rRNAs	574	1,928
tRNAs	433	422
**NBARCs (FindPlantNLRs annotation)**	**762**	**733**
**NLRs**	**676**	**652**
**BUSCO complete (proteome; *n* = 1,614)**	**99.7% (1,610)**	**99.7% (1,610)**
Single-copy (proteome)	84.9% (1,371)	85.0% (1,372)
Duplicated (proteome)	14.8% (239)	14.7% (238)
BUSCO fragmented (proteome)	0.1% (2)	0.1% (2)
BUSCO missing (proteome)	0.2% (2)	0.2% (2)
**Merqury QV**	**62.3**	**62.3**
**Repeats**	**33.1%**	**33.9%**

† At least half of the bases occur in a contig/scaffold of N50 bp or greater.

‡ L50 is the number of contigs/scaffolds of length N50 bp or greater.

A novel pipeline to identify and classify NLRs

We developed a comprehensive pipeline to annotate predicted NLR genes from an unmasked genome fasta file input. The rationale for an unmasked sequence is that the repetitive nature of the NLRs, regions may be missed with standard annotations [57]. Our pipeline, named FindPlantNLRs [43] utilises three key approaches. We combined loci identified using (1) NLR-annotator software [58] with (2) a basic local alignment search tool (tblastn) [59] using recently compiled and functionally validated NLR amino acid sequences and (3) a nucleotide iterative Hidden Markov Model (HMM) [60] to locate NBARC domains in genomes [61,62]. While the pipeline was developed to seek NLR genes within Myrtaceae genomes, the supplied NBARC HMMs are suitable for any plant genome search due to the iterative step that builds a unique species-specific HMM combined with the use of two other steps that incorporate broader models. The loci identified through these methods, and including 20 kb flanking regions, are then annotated with Braker2 software [63] using protein hints from experimentally validated resistance genes [34]. Annotated amino acid fasta files are screened for domains using Interproscan [64] and the predicted coding and amino acid sequences containing both NBARC and LRR domains are located back to scaffolds and extracted using additional scripts available on GitHub. To identify all classes of annotated NLRs, we developed a script that sorted and classified the “gene” types. We ran the file outputs from FindPlantNLRs with the NLR classification script [43]. To further identify novel predicted integrated domains in the annotated NLRs, we developed a script to search the data based on PFAM domain identities not classically associated with NLRs [43]. While our analyses have focussed on full-length NLRs, output from the pipeline also includes truncated NB containing genes. These files have been made available on GigaDB for future analyses.

Figure 4. Workflow of the FindPlantNLRs pipeline: a tool for annotating nucleotide-binding and leucine-rich repeat (NLR) genes. The pipeline annotates predicted NLR genes from an unmasked genome fasta file input. We combine loci identified using NLR-annotator software with a basic local alignment search tool (tblastn) using recently compiled and functionally validated NLR amino acid sequences and a nucleotide iterative Hidden Markov Model (HMM) to locate NBARC domains in genomes. The loci identified (including 20 kb flanking regions) are then annotated with Braker2 software using protein hints from experimentally validated resistance genes. Annotated amino acid fasta files are screened for domains using Interproscan and the predicted coding and amino acid sequences containing both NB-ARC and LRR domains are located back to scaffolds and extracted in gff3 format.

NLR number is variable across chromosomes and haplotypes

Using the FindPlantNLRs pipeline, we identified 762 putative NBARC containing genes in Haplotype A and 733 in Haplotype B based on the presence of the NBARC domain (Table S2). As canonical NLRs require both NBARC and LRR regions to be functional, for downstream analyses we were interested in isolating full gene models (genes containing both domains). Termed NLRs from hereon, we have divided these into genes containing a TIR domain (TNL), a CC or Rx domain (CNL), and those lacking TIR or CC domains (NL). Of the 762 NBARC containing genes in Haplotype A, we predicted 676 NLRs of which 67 lacked an N-terminal CC or TIR domain (Table S3). We excluded 86 predicted genes as they did not fit the definition of full genes models, with 68 lacking a C-terminal LRR domain and 18 lacking both N and C terminal domains (Table S2). Of the 733 NBARC containing genes in Haplotype B, we predicted 652 full gene models of which 71 lacked an N-terminal CC or TIR domain (Table S3). We excluded 81 predicted genes as they did not fit the definition of full genes models, with 61 lacking a C-terminal LRR domain and 20 lacking both N and C terminal domains (Table S2).

As NLR numbers differed between haplotypes, we sought to further investigate this difference at the chromosome level. The number of NLR genes per chromosome varied by up to 31 genes between haplotypes, with only chromosomes 1 and 9 containing the same number of genes across Haplotypes (Figure 5A). In Haplotype A, chromosomes 2 contained the highest number of NLR genes followed by chromosomes 5 and 3, while chromosome 5 contained the highest number of genes followed by chromosomes 3 and 2 in Haplotype B (Figure 5A). Upon further investigation, we determined the classes of NLRs is also consistent across chromosomes 1 and 9, while on all other chromosomes the number of NLRs in each class is variable. (Figure 5B and C). Chromosome 1 was also the only chromosome to contain NLRs of one class (CNL) (Figure 5B and C).

Figure 5. Summary of the number of predicted NLR genes per chromosome in the phased *Melaleuca quinquenervia* genome. (A) Comparison of the number of putative NLR genes on each chromosome in Haplotypes A and B. Putative NLRs were classified into TIR-NLR (TNL), CC-NLR and Rx-NLR (CNL) and NL classes on individual chromosomes in (B) Haplotype A and (C) Haplotype B.

NLR genes are arranged in clusters with hotspots on chromosomes

To visualise the physical clustering of NLRs on chromosomes, we mapped gene locations to chromosomal locations in both Haplotypes (Figure 6A and B). Employing the definition of a cluster as being a genomic region with 3 NLRs less than 250 kb apart with fewer than 8 other genes between each NLR, we determined variation in the number of genes clustering per haplotype, and clusters per chromosome within and between haplotypes. At a gene level, we determined 89.8% of genes in Haplotype A and 90.5% of genes in Haplotype B occur in clusters. A total of 51 clusters were identified in Haplotype A with an average of 4.6 clusters per chromosome and an average of 11.7 genes per cluster. A total of 50 clusters were identified in Haplotype B, averaging 5 clusters per chromosome and an average of 11.4 genes per cluster. 5.1% of genes were determined to occur as singles in Haplotype A and 5.1% as pairs. 6.1% of genes in Haplotype B were determined to occur as singles and 3.4% as pairs. In both haplotypes, the most clusters were on chromosome 5 (11 and 15 on Haplotypes A and B respectively) and the least (one cluster) on chromosome 9 in both Haplotypes (Figure 8A and B). The independently assembled and annotated assemblies based on ONT data verified the location of the majority of NLRs (Figure S5).

To investigate the role of assembly quality and completeness on NLR identification and clustering, we identified the closest ortholog in the other haplotype for each NLR gene, and plotted these relationships along with the positions of assembly gaps (Figure S6, S7). Whilst a few NLR clusters had assembly gaps in one or other haplotype, there were no obvious cases where a haplotype-specific expansion could be explained by a gap corresponding to the homologous region (Figure S7, S8). We then determined if these clusters were comprised of genes of the same class. We defined classes of clusters by clusters containing only genes of one class along with *NL*-type genes, otherwise they are considered mixed. TNL-type clusters were the most abundant clusters in both haplotypes and most abundant on chromosomes 3 and 5 in Haplotype A and chromosome 5 in Haplotype B (Figure 6C and D). CNL-type clusters were more evenly distributed across chromosomes in both haplotypes, with chromosome 2 containing the most clusters (4 in Haplotype A and 5 in Haplotype B) (Figure 6C and D).

Figure 6. Physical clustering of predicted NLR genes in the phased *Melaleuca quinquenervia* genome. Physical locations of predicted NLR genes on the chromosomes of *Melaleuca quinquenervia* (A) Haplotype A and (B) Haplotype B generated using ChromoMap in RStudio. The number of clusters per chromosomes in (C) Haplotype A and (D) Haplotype B was analysed and categorised based on the classes of all NLR genes.

Integrated domains are unique between haplotypes

Based on PFAM domain identities of the predicted NLR genes, we discovered 4.8% of NLRs in Haplotype A contain integrated domains (IDs) (Figure 7A), of which 44% contain more than one unique domain. Similarly, we observed a comparable percentage of 4.5% in Haplotype B (Figure 7B), with 37% of the predicted genes containing multiple unique domains. We also examined the number of ID-containing NLRs per chromosome and noted that in Haplotype A, chromosome 3 had the highest count with seven while chromosome 11 had none. In Haplotype B, chromosome 3 had six ID-containing NLRs, and 11 also had none (Figure 7C). During our investigation, we identified 48 unique IDs across both haplotypes. Interestingly, we found 23 IDs were exclusive to Haplotype A but only eight were exclusive to Haplotype B (Table S4). The remaining IDs were identified in both haplotypes (Table S4).

Figure 7. The NLR gene complement in the phased *Melaleuca quinquenervia* genome. The two sets of chromosomes corresponding to (A) Haplotypes A and (B) B were independently classified and visualised to present the domain classes using Sankeymatic [65] including the types and counts of integrated domains (IDs) with abbreviations derived from Pfam database [43]. NB = Nucleotide Binding Domain, TIR = Toll/Interleukin-1 receptor, JAC = Jacalin Domain, Rx = Potato CC-NB-LRR protein Rx, Coil = Coil-Coil Domain, RPW8 = RESISTANCE TO POWDERY MILDEW 8-like coiled-coil (C) The number of ID-containing NLRs per haplotype and chromosome in both haplotypes.

NLRs cluster into two distinct clades

The evolutionary relatedness of the 1,328 NBARC domains (462 CNL, 726 TNL, and 140 NL) from complete NLR genes models separated into two major clades: CNL (CNL, RxNL and RNL genes combined) and TNL genes (Figure 8). Fifty-nine percent of all sequences aligned with the TNL (784) clade and forty-one percent of total sequences aligned with the *CNL* clade (544) with 98 of the 140 NL sequences aligned with CNL and 42 aligned with TNL clades (Figure 8). Fifteen *CNL* NBARC sequences clustered within the TNL clade, however no TNLs clustered within the CNL clade. On closer inspection of these fifteen NBARC amino acid sequences, we determined that the integrity of the tree is correct due to the lack of the 'W' (tryptophan) at the 'LDD*W' kinase 2 sub-domain (Figure S9). This is canonical for CNL clade NBARC domains but not present in TNL clade [62]. We inspected the annotation and classification from FindPlantNLRs and found coiled-coil and Rx domains at the amino-

terminus on these fifteen gene models, hence the classification. It should be noted that all other NLR analyses in our study are based on the full annotated gene classification.

Figure 8. Evolutionary relationship of NBARC domains from predicted NLR genes within the phased *Melaleuca quinquenervia* genome. The NBARC domain fasta file and additional NBARC sequences, as outgroups, from functionally validated plant NLRs [34], were aligned with clustal-omega (v1.2.4) [121]. The phylogenetic tree was inferred with the alignment file using IQ-TREE (v1.6.7) [122] and visualised in iTOL (v5) [123]. Each tip represents one putative *NLR* gene with branch lengths signifying rates of amino acid substitutions. Colours indicate the CNL (including RxNLs) (pink), TNL (blue) and NL (yellow) clades. Scale = 0.1 amino acid substitutions per site. The interactive tree can be viewed at https://itol.embl.de/shared/alyssamartino.

Transcript evidence found for predicted NLRs

To confirm that in-silico NLR predictions were actively expressed, we downloaded RNAseq data from a previous *M. quinquennia* study that investigated responses to the plant pathogen causing myrtle rust [66]. We mapped all the available RNA-Seq data to the NLR coding sequencing for each haploid genome independently using Hisat2 [67]. Taking the transcripts per million (TPM) cut-off of 50, we determined expression for 617 and 596 NLR coding sequences from Haplotype A and B respectively. The most abundantly expressed predicted NLR gene is an *RPW8* (PF05659) *NLR* homologue, TPM 50,744 and 47,856 for Haplotype A and B respectively. This gene is predicted on chromosome 6, NLR gene identifications, g7145.t1 and g1651.t1 respectively (Table S3).

Discussion

A high-quality diploid genome for the keystone wetland species, Melaleuca quinquenervia

To promote scientific investigation, we have assembled a telomere-to-telomere diploid genome for a keystone wetland species, the broadleaved paperbark tree, *Melaleuca quinquenervia*. Using ~70x HiFi coverage (35x per haplotype), combined with ~380x Illumina Hi-C coverage, our assembly scaffolded into the expected 11 Myrtaceae chromosomes ($2n$ = 22) and has a very high level of BUSCO completeness (Table 2). With careful curation to remove scaffolding errors and misassemblies, followed by polishing, we numbered two sets of parental chromosomes in accordance with the Myrtaceae reference genome, an inbred clone of *Eucalyptus grandis* [7]. We were able to show synteny between the *M. quinquenervia* chromosomes with five other publicly available chromosome-level Myrtaceae genomes (Figure 2). Additionally, the genome and subsequent analyses were independently validated with scaffolded assemblies using ~234x ONT data. Based on homology with three publicly available Myrtaceae proteomes and with *A. thaliana*, we predicted 28,744 and 28,517 protein coding genes within the two chromosome sets. These numbers are slightly less per haplotype, but comparable to the predicted 36,779 for the haploid genome of *E. grandis*. This is likely to be due to the earlier generation sequencing technology, assembly software and the result of collapsed assemblies for highly heterozygous plants. We annotated repetitive genomic regions at ~33% in both haplotypes, compared to 41 and 44% in *E. grandis* [7] and *E. pauciflora* [68] respectively, likely related to the smaller genome size for *M. quinquenervia*. There was a marked difference in rRNA content between the two haplotypes and these differences are being driven by rRNA on unanchored contigs. Our curated assembly meets the high standards and metrics of the vertebrate genome project objectives [69] providing an exceptional resource for functional molecular and evolutionary studies.

A smaller than predicted genome for Melaleuca quinquenervia

A 2C-value of 1.94 was previously reported in the literature using flow cytometry on samples from a tree in a university garden [69]. We therefore expected the genome size for each haploid assembly to be 949 Mb and planned our sequencing experiments accordingly. The *M. quinquenervia* genomes we assembled are much smaller, at ~270 Mb, and polyploidy has not been reported in this species. The authors on the flow cytometry study reported problems processing their Myrtaceae samples, perhaps explaining the large size discrepancy in these results. To test that our results were accurate, we checked the ploidy and ran *k*-mer and read depth-based analyses, as described in the methods. Results indicated the genome was 270-280 Mb, less than half the size of the *E. grandis* genome at 640 Mb [7]. While the genome size was surprising, we were able to use the high sequence coverage to ensure a highly accurate diploid genome.

The annotated NLR complement for both Melaleuca quinquenervia chromosome sets

With the high quality of our genome, we were able to comprehensively annotate the NLR-type resistance genes in both inherited chromosome sets, using our novel FindPlantNLRs pipeline. Of the 1,495 annotated NBARC containing genes identified in the *M*. *quinquenervia* genome (Figure 5), we determined that 1,328 were complete NLRs while a further 167 contained the NBARC domain but lacked either, or both, the C or N-terminal domains. The number of NBARC containing genes in the genome is consistent with analysis of *E. grandis* which was determined at 1487 NBARC containing genes [62] despite a much larger genome size. Although genome size is not directly correlated with NLR content [70], the presentation of *E. grandis* genome in its collapsed form may result in underrepresentation of the NLRs as allelic variants. We estimated 125 genes in Haplotype A had no ortholog in the alternate Haplotype, while 107 from Haplotype B had no ortholog in the alternate Haplotype (Figure S6, S7). To our knowledge, this is the first published research that has presented the allelic NLR complement in a phased, chromosome-level genome. As such, analysis of orthologs between haplotypes is limited to currently available software which is designed to compare species. The software limitation may therefore lead to some discrepancies in ortholog numbers within our analyses (Figure S6, S7). Nonetheless, our detailed analysis highlights unique allelic variation that will assist research into the reported different phenotypic responses to pest- and pathogen-challenged species with the family Myrtaceae [66]. Our data might also be useful for understanding the strong evolutionary selection pressures on these plant immune receptors that has resulted in the allelic variation we present for *M. quinquenervia*. Analysis of gene families such as NLRs may also assist in understanding how invasive species manage to escape native-range microbes, as is the case for *M. quinquenervia* in Florida where it is exposed to a new suite of microbes [71].

Melaleuca quinquenervia NLRs are dominated by TNL-type resistance genes

Consistent with the *E. grandis* NLR annotation, is the higher proportion of TNL to CNL type genes supporting an expansion of the TNL clade within the Myrtaceae [62]. This is further validated by recent phylogenetic analyses using transcripts from *M. quinquenervia* and *M. alternifolia* which revealed approximately two thirds of NLR transcripts clustering with TNLs from *E. grandis* [72]. We found TNL to CNL ratios of ~3:1 in Haplotype A and ~3:2 in Haplotype B of *M. quinquenervia*. The ID containing NLRs had a greater proportion of TNLs than CNLs with IDs (~2:1 and 3:1 in Haplotypes A and B respectively). The TIR domain has been demonstrated to play a key role in the self-association of the NLR proteins to form higher order resistosomes which are necessary for immune signalling [73]. Of particular interest of the TNL-type genes annotated, are those containing a C-terminal jacalin domain, and no LRR domain (Figure 7). NLRs containing an alternative C-terminal domain have been identified in a range of agriculturally important plant species such as wheat, rice, sorghum, and barley as well as tree species such as *Eucalyptus grandis*, *Syzygium luehmannii* and *M. quinquenervia* [62,72,74,75]. Unlike conventional NLRs which contain a C-terminal LRR domain, the LRR is replaced by a jacalin domain (PF01419), a mannose binding lectin. Although previously thought of as a decoy domain for pathogen effectors, the replacement of the LRR domain by a jacalin domain suggests that this domain may play a role in NLR function. The expansion of the TIR class combined with fused IDs within TNLs, discussed later, may provide novel defence capacity against pests and pathogens. Chromosomal locations for all truncated NLRs are available in GigaDB [xx].

Phylogenetic evolutionary analysis supports the NLR classification results

By combining all the NBARC amino acid domains from both haplotypes, we visualised the evolutionary relatedness of NLRs. While the phylogenetic tree was based on alignment of NBARC domains, and not full annotated genes, it demonstrated the clear divergence into CNL and TNL clades (Figure 8) as observed in other plant species [33,62]. Of the NLRs lacking CC or TIR domains (NLs), 42 are clustered in the TNL clade and the remaining 96 into the CNL clade. Of interest, the expansion of the TNL clade, also observed in *E. grandis* [62] with 53 percent TNL to 47 percent CNL, was comparable in *M. quinquenervia* with 59 percent TNL to 41 percent CNL (Figure 8). There were 15 predicted CNLs that clustered within the TNL clade. On inspection of these amino acid sequences, we found that they had coiled-coil or Rx-type domains fused to classic TNL-type NBARC domains. Two of these NLRs have homologues in the alternative haplotype lacking an N-terminal domain, and a one is homologous to a TNL gene. A further five have no homologous partner in the alternative haplotype, with the remaining seven homologous to the NLRs with swapped domains. These results suggest amino terminal domain

swapping as a possible evolutionary mechanism, however further functional and molecular validation is required.

NLR physical clusters on chromosomes in M. quinquenervia

Analysis of the putative TNLs, CNLs and NLs within the phased genome of *M. quinquenervia* revealed the majority of NLRs located within clusters, with 86% clustering in Haplotype A and 88% in Haplotype B. Only 14% and 12% from Haplotype A and B respectively did not fall into clusters, compared to approximately a quarter of *NLR*s in *Eucalyptus grandis* [62], cultivated rice (*Oryza sativa*) [77], and *A. thaliana* [33], employing the same method for determining clusters. For *M. quinquenervia*, there were approximately 5 NLR genes for every Mb of the total genome size while in *A. thaliana*, *E. grandis* and *O. sativa* the number of NLRs per Mb ranged from 1.2 to 2.3 [25,62,78]. The higher density of NLRs in the *M. quinquenervia* genome may explain the higher proportion of NLRs appearing in clusters. Closer inspection of NLR clusters revealed that some of the larger clusters overlapped with genome assembly gaps (Figure S6, S7). As NLRs are highly repetitive, this may be the result of challenged associated with assembling highly repetitive genomic regions. This has been observed for other multi-copy repetitive gene families such as the major histocompatibility complex family [79]. Nevertheless, the majority of NLRs are present at a read-depth consistent with correct copy numbers (Figure 3, S4 and S5), indicating that assembly difficulties in NLR repeats has not substantially affected results.

Most clusters were homogenous, containing NLRs of the same class, with only 4 heterogenous clusters in Haplotype A and 2 in Haplotype B (Figure 6C and D). The high proportion of homogenous clusters suggests the expansion of these genes into clusters is driven by tandem duplication [80], as a mechanism for maintaining NLR diversity [81]. Clustering may also play an important role in pathogen resistance. NLR pairs such as *RGA4* and *RGA5* [82] and *Pik-1* and *Pik-2* in cultivated rice [83] are oriented in a head-to-head manner, and function cooperatively in pathogen recognition and response, with one acting as sensor of the pathogen and the other as an executor of immune signalling. This was also observed for the NLR pair *RPS4* and *RRS1* in *A. thaliana*, suggesting a shared promoter for the co-regulation of the two genes [84,85]. Interestingly, for each of these pairs, one partner from each contained an ID. On chromosome 3 of Haplotype B of *M. quinquenervia*, one pair of NLRs was identified in this head-to-head manner, with one partner containing one RVT2 and one gag_pre-integrs ID. The identification of genes in the head-to-head manner in *M. quinquenervia* may indicate a functional role for these genes in disease resistance, with further studies needed to elucidate a potential function.

The NLR repertoire is unique between haplotypes

Overall, the patterns of individual NLR numbers, classes, clusters, and cluster types across chromosomes appear consistent between the two haplotypes of *M. quinquenervia* (Figure 5 and Figure 6). However, analysis at the individual chromosome and gene level revealed diversity in the number and classes of genes between haplotypes for all except chromosomes 1 and 9 (Figure 5). While consistent in gene number, and gene number per class, analysis of the IDs across chromosome 1 revealed one gene on Haplotype B to contain two DUF642 domains which was not present on the corresponding gene in Haplotype A. Similarly, one gene in Haplotype A of chromosome 9 contained one NAD_binding_11 and one NAD_binding_2 domains which were not present in the corresponding gene on Haplotype B (Table S3). The presence/absence NLR polymorphisms between the haplotypes of *M. quinquenervia* are likely explained by the outcrossing nature of the species. High levels of genetic diversity maintained in long-lived, outcrossing woody species [86], combined with exposure to a range of pests and pathogens over their lifetime, may lead to changes in NLRs arrangement over subsequent generations. Presence/absence polymorphisms of NLRs has been observed in several plant species such as between inbred accessions of *O. sativa* and *A. thaliana* [87,88]. This may be explained by the fitness cost associated with the maintenance of these genes [89], leading to loss of corresponding genes in the absence of the pathogen.

We identified a total of 53 unique IDs across both haplotypes, accounting for 4.4% of NLR genes in Haplotype A and 6.8 % in Haplotype B. These figures are consistent with a recent review of published NLR-ID analyses that revealed 3.5 – 14% of NLRs contained IDs [27]. These fused integrated domains appear to mimic host proteins that are targets for pathogen effectors, leading to the triggering of defence response [26]. Some of the most commonly occurring integrated domains belong to families of proteins with critical roles in plant defence [26,90] such as WRKY transcription factors and BED zinc fingers (BEAF and DREF from *Drosophila melanogaster* peptide; zf-BED). In the genome of *M. quinquenervia*, one of the most commonly occurring ID was the WRKY domain which was identified in five genes across the two haplotypes. A notable example of the role of an integrated WRKY domain present in an NLR, is the *Arabidopsis Ralstonia solanacearum gene 1* (*RSS1-R*) [28,91]. Bacterial effectors were found to bind to the WRKY domain of the NLR protein and other WRKY containing proteins [91], suggesting a role for this domain as a decoy. Another common domain was the zf-BED domain which was identified in seven genes across the two haplotypes. While the function of the ID is yet to be elucidated, zf-BED domains have been observed in NLR genes conferring resistance to rust pathogens in barley, wheat, and rice [92–96]. The identification of these fused domains suggests a role for these genes in pathogen recognition.

Potential implications

Long-lived tree species must respond to a wide range of biotic stresses. Our results provide insight into the diversity of the NLR gene family within a single host tree species, indicating a potential mechanism for responses to invasive pathogens over a lifespan. We provide a framework for studying highly repetitive resistance genes by generating a high-quality pseudo-phased reference genome. With advances in sequencing and software, we are beginning to investigate the full repertoire of all genes, including NLRs, here starting with a representative Myrtaceae tree, *Melaleuca quinquenervia*. Given the diversity of NLRs from just two haplotypes, our results indicate that association studies of outcrossing species will need to model presence/absence of NLRs, in addition to segregating sequence variants. Future studies may expand to comparing population level diversity of NLRs and the diversity of NLRomes across woody plants.

Methods

DNA extraction and sequencing

Sampling and DNA extraction

We obtained young fresh leaves (approximately 30 g) from a mature *Melaleuca quinquenervia* (Cav.) S.T. Blake tree growing at the Royal Botanic Gardens (RBG) Sydney, New South Wales (BioSample accession SAMN20854364) for use as the reference genome individual. We chose this specimen for the ease of ongoing access to leaf, cuttings, and seed material. The tree was planted in 1880 by HRH Prince George of Wales, later King George V. The tree is now 140 years old, of unknown provenance, and is showing signs of senescence.

For PacBio HiFi sequencing, we extracted high molecular weight (HMW) genomic DNA (gDNA) using two sorbitol washes [97] followed by a CTAB/NaCl/Proteinase K protocol [98]. We purified gDNA with two rounds of bead clean-up (AMPure Beads) and assessed resulting gDNA quality using Nanodrop2000 and Qubit 2.0 Fluorometer (dsDNA HS assay) to obtain a minimum ratio of 0.6.

For Oxford Nanopore Technologies (ONT) Nanopore sequencing, we extracted HMW gDNA using a magnetic bead-based protocol described in [96]. We subsequently size selected the gDNA for fragments ≥ 40 kb using a PippinHT (Sage Science).

PacBio HiFi sequencing

We sent the final HMW gDNA sample of ~100 µL, 451.7 ng/µL in 10 mM TrisHCl (~45 µg HMW) to the Australian Genome Research Facility Ltd (AGRF), St Lucia, Queensland for HiFi 10-15 kb fragment gDNA Pippin Prep size selection, library preparation and PacBio Sequel II sequencing (SMRT Cell 8M).

Hi-C proximity-ligation sequencing

Hi-C library preparation and sequencing was conducted at the Ramaciotti Centre for Genomics using the Phase Genomics Plant kit v3.0. A pilot run on an Illumina iSeq 100 with 2 x 150 bp paired end sequencing run was performed for QC using hic_qc v1.0 (Phase Genomics, 2019) with i1 300 cycle chemistry. This was followed by sequencing on the Illumina NextSeq 500 with 2 x 150 bp paired-end high output run and NextSeq High Output 300 cycle kit v2.5 chemistry.

ONT Sequencing

We prepared a long-read native DNA sequencing library according to ONT protocol Genomic DNA by Ligation (SQK-LSK110). We performed sequencing on an ONT PromethION using a FLO-PRO002 R9.4.1 flow cell, with three wash treatments and reloads to maximise output, according to the manufacturer's Flow Cell Wash Kit (EXP-WSH004). We basecalled the fast5 reads to fastq with Guppy basecaller (RRID:SCR_023196) v6.1.2 (model_version_id=2021-05-05_dna_r9.4.1_promethion_768_922a514b), inspecting the output and quality with NanoPlot [99].

Genome size prediction

We computed HiFi CCS read Kmer frequencies using Jellyfish v2.2.10 [100] and KMC v3.1.1 [101], with k=19 and a maximum kmer frequency of 10,000 (-k19 -ci1 -cs10000). We used the GenomeScope v2.0 webserver [50] to predict genome sizes.

We carried out additional genome size prediction using single-copy read depth analysis by DepthSizer v1.4.0 [49]. We mapped HiFi CCS and ONT reads to each genome assembly analysed using minimap2 v2.22 [102], and calculated BAM depth and coverage statistics with SAMTOOLS (RRID:SCR_002105) v1.13 [103]. We used single-copy genes identified as "Complete" by Benchmarking Universal Single Copy Orthologs (BUSCO) for each assembly. We generated genome size plots with the ggstatsplot package [104] in R v4.1.0.

Genome assembly and Hi-C scaffolding

We assembled the genome with the Hifiasm (RRID:SCR_021069) v0.15.5 [44] package using PacBio HiFi reads and integrating Hi-C reads. We independently scaffolded genome outputs using the Aiden Lab pipelines [45,46] (assembly v0.1; Figure S3A and B). The assignment of scaffolds to either Haplotype A or B was determined by hifiasm arbitrarily as the parent trees were not available to be sequenced. The ONT data were assembles with Flye (v2.9) [105], polished with Hypo (v1.0.3) [106] and scaffolded with Hi-C data (Figure S1C & D). To scaffold the genomes, we ran the Juicer pipeline (v1.6) [107] with default parameters. To ensure that all duplicate mapped reads were removed, we renamed the merged_sort.txt output from Juicer and reformatted and renamed the merged_nodups.txt to replicate the format of the original merged_sort.txt with the script "cat

merged_nodups.txt |sort --parallel=16 -k2,2d -k6,6d > merged_sort.txt". We reran Juicer using the newly created merged_sort.txt with additional parameter "-S dedup" and used the final output with the 3D-DNA pipeline (v180922) [47] with the following parameters "-m haploid --build-gapped-map --sort-output". After we manually curated the assemblies locally within the Juicebox visualisation software (v1.11.08 for Windows) [46], we resubmitted the revised assembly file to the 3D-DNA post review pipeline with the parameters "--build-gapped-map --sort-output" for final assembly and fasta files.

Assembly curation, filtering, and polishing

We tidied Hi-C scaffolds with Diploidocus (RRID:SCR_021231) (v0.18.0) [49] dipcycle mode, using the HiFi reads for both long reads and high accuracy (kmer) reads (assembly v0.2) with each haplotype filtered independently. We assigned chromosomes with PAFScaff (v0.4.1) [108], mapping on to the *Eucalyptus grandis* (GCF_000612305.1) chromosomes (assembly v0.3), and visually compared the two haplotypes, using SynBad (v0.8.4) [109] and DepthKopy (v1.1.0) [49] as guides. We identified some scaffolding errors, which we manually corrected (assembly v0.4) before a second round of Diploidocus tidy on each haplotype (assembly v0.5). We used DepthCharge (v0.2.0) [110] was used to assess for misassemblies, with none identified, however we failed to close any assembly gaps using LR Gapcloser (RRID:SCR_016194) (v20180904).

Next, we mapped the HiFi reads onto the diploid assembly with Minimap2 (RRID:SCR_018550) (v2.22) [102] and partitioned by haplotype. We separated non-chromosome scaffolds into contigs ran a third round of Diploidocus tidy on each haplotype using the appropriate subset of haplotype-mapped HiFi reads (assembly v0.6).

We then polished the tidied diploid genome with HyPo (v1.0.3) [106] using the HiFi reads mapped with Minimap2 (v2.22) [102] for both the long read and high accuracy data (assembly v0.7). Finally, we renamed the chromosomes according to synteny with the *Eucalyptus grandis* genome [7] to produce v1.0 of the *M. quinquenervia* genome.

Genome completeness, validation, and annotation

To determine genome completeness, we used BUSCO (v5.3.1) [54] using the lineage dataset embryophyta_odb10. Additionally, we estimated genome assembly quality (QV) using *k-mer* analysis of HiFi read data by Merqury (RRID:SCR_022964) v1.0 with k = 21 [53].

We used the homology-based gene prediction program GeMoMa (v1.7.1) [55] to annotate the genome, utilising four reference genomes downloaded from NCBI: *Arabidopsis thaliana* (TAIR10.1, GCA_000001735.2), *Eucalyptus grandis* [7] (GCF_000612305.1), *Syzygium oleosum*

(GCF_900635055.1) and *Rhodamnia argentea* (GCF_020921035.1). We predicted Ribosomal RNA (rRNA) genes with Barrnap (v0.9) [111] and transfer RNAs (tRNAs) with tRNAscan-SE (v2.05) [112], implementing Infernal (v1.1.2) [113] filtering for eukaryotes using the recommended protocol to form the high-confidence set. To generate a custom repeat library, we used RepeatModeler (v2.0.1) [56] following genome masking using RepeatMasker (RRID:SCR_012954) (v4.1.0) [114], both with default parameters. We generated the annotation table using the buildSummary.pl RepeatMasker script.

Synteny to other Myrtaceae

We used Chromsyn [48] to investigate synteny of *M. quinquenervia* to five chromosome-level Myrtaceae genomes available on NCBI: *Angophora floribunda* (GCA_014182895.1) [115], *Eucalyptus grandis* [7] (GCF_016545825.1), *Rhodamnia argentea* (GCF_020921035.1), *Psidium guajava* (GCA_016432845.1) [116] and *Syzygium aromaticum* (GCA_024500025.1) [117]. We ordered the species according to phylogenetic relationships [118].

NLR Analysis

NLR annotation with FindPlantNLRs

We developed a comprehensive pipeline to annotate predicted NLR genes from an unmasked genome fasta file input, named FindPlantNLRs [43]. The complete described protocol including software version, dependencies, HMMs and additional scripts are available on GitHub [43].

Classification of annotated NLRs and identification of integrated domains

To identify all classes of annotated NLRs, we developed a script that sorted and classified the "gene" types. We ran the file outputs from FindPlantNLRs with the NLR classification script [43]. To further identify novel predicted integrated domains in the annotated NLRs, we developed a script to search the data based on PFAM domain identities not classically associated with NLRs [43]. Resulting files were then sorted to identify the predicted NLR genes by classification and integrated domains per phased genome. The formatted lists were then input to the web-based site sankeymatic.com/build/ to create flow diagrams [65]. For all analyses downstream of the FindPlantNLRs pipeline, we included only full NLR gene models which was defined as those genes containing both an NBARC domain and an LRR domain.

NLR cluster, duplicated gene, and ortholog analysis

Clustering analysis was based on previous analyses in *E. grandis* and *A. thaliana* genomes [62,119]. We defined a cluster as a genomic region containing three or more predicted *NLR* genes, each of which less than 250 kb from a neighbouring *NLR* gene and with less than 8 non-*NLR* genes between each *NLR*.

We followed the *E. grandis* definition of class classification of *NLR* [62]. *CNL*-type clusters were defined by those containing at least one gene with a *CNL* domain, and no *TNL* type domains. *TNL*-type clusters were defined as those containing at least one gene with a *TNL* domain, and no *CNL* domains. *NL* clusters were defined by those containing only genes with no N-terminal domains. Mixed type clusters were defined as those containing at least two genes with differing N-terminal domains, or lack of N-terminal domain. We visualised the positions of individual *NLRs* and *NLR* clusters on *M. quinquenervia* chromosomes with ChromoMap [120] using base pair start and end positions.

We investigated genome-wide copy numbers using DepthKopy (v1.1.0) [49] for the HiFi and ONT assemblies, with analysis of the HiFi and ONT read data, examining the BUSCO genes, *NLR* annotations, *NBARC* regions, scaffolds and 100 kb windows across the genome.

To identify orthologs, we aligned sister chromosomes of *Melaleuca quinquenervia* with minimap2 (2.24-r1122) [102] with -cx asm20 and alignments were filtered with 'length ≥1000bp and identity ≥90%'. We used GOPHER (v3.5.4) [121] to determine orthologs between haplotypes with default settings and used BEDTools (RRID:SCR_006646) intersect (v2.27.1) [122] to identify NLRs which located in unaligned regions. Dot plots were generated with ggplot2 (v3.4.2) [123]. Syntenic graphs were generated with karyoploteR (RRID:SCR_021824) (1.26.0) [124] with nucleotide aligned regions from minimap2 (2.24-r1122) [102]. Gaps in the assembly were rated as either Syntenic (both sides map in the correct order and orientation to the alternative haplotype), or non-syntenic (mismatched best-matching scaffolds from the alternative haplotype for each side of the gap) using SynBad ratings [109].

Phylogenetic analysis of Melaleuca quinquenervia NLRs

To investigate relatedness among NLR genes, we extracted all NBARC domains from the annotated amino acid files for both sets of scaffolds using the chromosome locations with bedtools (v2.29.2) [121]. We included an outgroup of amino acid NBARC domains taken from a subset of functionally validated plant NLRs [34]. We reduced the outgroup set to include NBARC domains from eudicotyledons only and incorporated six CNL, two RPW8 and seven TNL-type NBARC domains. We removed 81 predicted transcripts annotated as t2, retaining only t1 predicted reads, from the phased *M. quinquenervia* data and combined the remaining NLR NBARC domains with the outgroups. We aligned the combined sequences with clustal-omega (v1.2.4) [125], and inferred the phylogenetic tree with IQ-TREE [126] using the following parameters, -bb 1000 -st AA -m LG. We visualised the resulting newick file with iTOL [127] and colour coded according to NLR clade.

To investigate the homologues of the 15 NLRs containing mismatched N-terminal and NBARC domains, we ran Proteinortho (RRID:SCR_024177) (v6.0.15) [128] on the NLRs used for phylogenetic analysis with BLASTP run using DIAMOND (RRID:SCR_009457) (v2.1.6) [129].

Transcript evidence for annotated NLRs in Melaleuca quinquenervia

To test for expression evidence for our annotated NLR genes, we downloaded RNASeq data (NCBI PRJNA357284) from a previous *M. quinquenervia* study that investigated responses to the plant pathogen causing myrtle rust [66]. We mapped all the available RNASeq data to the NLR coding sequences for each haploid genome independently using Hisat2 (v2.1.0) [67] with the parameters “hisat2 -p 16 --summary-file MqA/MqB --trim5 15 --trim3 10 --no-unal -p 16 -S <file.sam>”. We processed the sam file outputs with samtools (v1.9) [103] for sorted and indexed bam files and obtained mapping statistics with samtools idxstats. Finally, we calculated the transcripts per million (TPM) for all predicted NLR genes.

Availability of source code and requirements

Project name: FindPlantNLRs

Project home page: https://github.com/ZhenyanLuo/FindPlantNLRs [43]

Operating system(s): Platform independent

Programming language: Python

Other requirements: none.

License: GPL 3.0

Any restrictions to use by non-academics: none

RRID: REQUEST AUTHORS TO REGISTER ON SCICRUNCH.ORG AND ADD RRID NUMBER HERE

Data availability

The resistance gene annotation tool is available at https://github.com/ZhenyanLuo/FindPlantNLRs [43] and is registered on bio.tools (https://bio.tools/findplantnlrs). The genome assemblies and raw sequencing data are available on NCBI under the Umbrella BioProject PRJNA756045 which is linked to the HapA assembly and the raw data used to generate both haplotypes; the HapB assembly was deposited to BioProject PRJNA911843. Other data further supporting this work are openly available in the GigaScience repository, GigaDB [76].

List of Abbreviations

BUSCO (Benchmarking Universal Single-Copy Orthologs)

CC (coiled-coil)

CN (coiled-coil nucleotide binding)

CNL (coiled-coil nucleotide binding leucine rich repeat)

HR (hypersensitive response)

LRR (leucine rich repeat)

NBARC (nucleotide binding Apaf-1, R-protein and CED-4)

NB (nucleotide binding)

NL/NLR (nucleotide binding leucine rich repeat)

ONT (Oxford Nanopore Technologies)

RBG (Royal Botanic Gardens)

RPW8/CC-R (RESISTANCE TO POWDERY MILDEW 8-like coiled-coil)

RxNL (Potato CC-NB-LRR protein Rx nucleotide binding leucine rich repeat)

TIR (Toll/Interleukin-1 receptor/ Resistance protein)

TN (Toll/Interleukin-1 receptor/ Resistance nucleotide binding)

TNL (Toll/Interleukin-1 receptor/ Resistance nucleotide binding leucine rich repeat)

Consent for publication

Not applicable.

Competing interests

The authors declare that they have no competing interests.

Funding

SHC and AMM were supported through an Australian Government Research Training Program Scholarship. The Australian Research Council funded RJE and JBG (LP18010072) and PAT and BS (LP190100093).

Author contributions

SHC, AMM, JGB, PAT and RJE planned the project. AMM, JGB, PAT, RJE, SHC, BS and AJ wrote the paper. Plant sampling was carried out by AMM, JGB, PAT and SHC and DNA extraction by AMM, PAT, SHC and AJ. AJ carrier out ONT sequencing. SHC, JGB produced the primary genome assembly and annotation as well as additional assembly curation and QC. PAT, SHC carried out Hi-C scaffolding. RJE conducted synteny and copy number analysis. PAT, BS, ZL and TT conceptualised and developed the FindPlantNLRs pipeline. NLR analyses were conducted by AMM and PAT and orthology analysis conducted by AMM and ZL. All authors provided valuable comments on the manuscript.

Acknowledgements

We thank Matt Coyne, David Laughlin and Scott Jones at the Royal Botanic Garden Sydney who assisted with sampling.

References

1. GBIF Secretariat. GBIF Backbone Taxonomy. 2022; Checklist dataset https://doi.org/10.15468/39omei accessed via GBIF.org on 2023-03-14.

2. Brophy JJ, Craven LA, Doran JC. *Melaleucas*: their botany, essential oils and uses. ACIAR Monograph No. 156; Australian Centre for International Agricultural Research; 2013.

3. Kubitzki K, Kallunki JA, Duretto M, Wilson PG. The families and genera of vascular plants. Volume X Berlin: Springer; 2011.

4. Turner CE, Center TD, Burrows DW, Buckingham GR. Ecology and management of *Melaleuca quinquenervia*, an invader of wetlands in Florida, USA. Wetl Ecol Manag. 1997; doi: 10.1023/A:1008205122757/METRICS.

5. Watt MS, Kriticos DJ, Manning LK. The current and future potential distribution of *Melaleuca quinquenervia*. Weed Res. 2009; doi: 10.1111/j.1365-3180.2009.00704.x.

6. Voelker J, Shepherd M, Mauleon R. A high-quality draft genome for *Melaleuca alternifolia* (tea tree): a new platform for evolutionary genomics of myrtaceous terpene-rich species. GigaByte. 2021; doi: 10.46471/gigabyte.28.

7. Myburg AA, Grattapaglia D, Tuskan GA, Hellsten U, Hayes RD, Grimwood J, et al.. The genome of *Eucalyptus grandis*. Nature. 2014; doi: 10.1038/nature13308.

8. Healey AL, Shepherd M, King GJ, Butler JB, Freeman JS, Lee DJ, et al.. Pests, diseases, and aridity have shaped the genome of *Corymbia citriodora*. Comms Bio. 2021; doi: 10.1038/s42003-021-02009-0.

9. Tobias PA, Guest DI. Tree immunity: growing old without antibodies. Trends Plant Sci. 2014; doi: 10.1016/j.tplants.2014.01.011.

10. Ziv C, Zhao Z, Gao YG, Xia Y. Multifunctional roles of plant cuticle during plant-pathogen interactions. Front Plant Sci. 2018; doi: 10.3389/FPLS.2018.01088/BIBTEX.

11. Yu Z, Shen K, Newcombe G, Fan J, Chen Q. Leaf cuticle can contribute to non-host resistance to poplar leaf rust. Forests. 2019; doi: 10.3390/f10100870.

12. Smith AH, Potts BM, Ratkowsky DA, Pinkard EA, Mohammed CL. Association of *Eucalyptus globulus* leaf anatomy with susceptibility to *Teratosphaeria* leaf disease. For Pathol. 2018; doi: 10.1111/efp.12395.

13. Manea A, Tabassum S, Fernandez Winzer L, Leishman MR. Susceptibility to the fungal plant pathogen *Austropuccinia psidii* is related to monoterpene production in Australian *Myrtaceae species*. Biol Invasions. 2022; doi: 10.1007/S10530-021-02721-2/FIGURES/3.

14. Trujillo-Moya C, Ganthaler A, Stöggl W, Kranner I, Schüler S, Ertl R, et al.. RNA-Seq and secondary metabolite analyses reveal a putative defence-transcriptome in Norway spruce (*Picea abies*) against needle bladder rust (*Chrysomyxa rhododendri*) infection. BMC Genomics. 2020; doi: 10.1186/s12864-020-6587-z.

15. Jones JDG, Dangl JL. The plant immune system. Nature. 2006; doi: 10.1038/nature05286.

16. Yuan M, Jiang Z, Bi G, Nomura K, Liu M, Wang Y, et al.. Pattern-recognition receptors are required for NLR-mediated plant immunity. Nature. 2021; doi: 10.1038/s41586-021-03316-6.

17. Cook DE, Mesarich CH, Thomma BPHJ. Understanding Plant Immunity as a Surveillance System to Detect Invasion. Annu Rev Phyto. 2015; doi: 10.1146/ANNUREV-PHYTO-080614-120114.

18. Ting JPY, Lovering RC, Alnemri ES, Bertin J, Boss JM, Davis BK, et al.. The NLR Gene Family: A Standard Nomenclature. Immunity. 2008; doi: 10.1016/j.immuni.2008.02.005.

19. Mur LAJ, Kenton P, Lloyd AJ, Ougham H, Prats E. The hypersensitive response; The centenary is upon us but how much do we know? J Exp Bot. 2008; doi: 10.1093/jxb/erm239.

20. Tameling WIL, Vossen JH, Albrecht M, Lengauer T, Berden JA, Haring MA, et al.. Mutations in the NB-ARC Domain of I-2 That Impair ATP Hydrolysis Cause Autoactivation. Plant Physiol. 2006; doi: 10.1104/PP.105.073510.

21. Shao ZQ, Xue JY, Wu P, Zhang YM, Wu Y, Hang YY, et al.. Large-scale analyses of angiosperm nucleotide-binding site-leucine-rich repeat genes reveal three anciently diverged classes with distinct evolutionary patterns. Plant Physiol. 2016; doi: 10.1104/pp.15.01487.

22. Chang C, Yu D, Jiao J, Jing S, Schulze-Lefert P, Shen QH. Barley MLA immune receptors directly interfere with antagonistically acting transcription factors to initiate disease resistance signaling. Plant Cell. 2013; doi: 10.1105/tpc.113.109942.

23. Williams SJ, Sohn KH, Wan L, Bernoux M, Sarris PF, Segonzac C, et al.. Structural basis for assembly and function of a heterodimeric plant immune receptor. Science. 2014; doi: 10.1126/science.1247357.

24. Bai J, Pennill LA, Ning J, Lee SW, Ramalingam J, Webb CA, et al.. Diversity in Nucleotide Binding Site–Leucine-Rich Repeat Genes in Cereals. Genome Res. 2002; doi: 10.1101/GR.454902.

25. Van de Weyer AL, Monteiro F, Furzer OJ, Nishimura MT, Cevik V, Witek K, et al.. A Species-Wide Inventory of NLR Genes and Alleles in *Arabidopsis thaliana*. Cell. 2019; doi: 10.1016/j.cell.2019.07.038.

26. Césari S, Bernoux M, Moncuquet P, Kroj T, Dodds PN. A novel conserved mechanism for plant NLR protein pairs: The “integrated decoy” hypothesis. Front Plant Sci. 2014; doi: 10.3389/fpls.2014.00606.

27. Grund E, Tremousaygue D, Deslandes L. Plant NLRs with integrated domains: Unity makes strength. Plant Physiol. 2019; doi: 10.1104/pp.18.01134.

28. Le Roux C, Huet G, Jauneau A, Camborde L, Trémousaygue D, Kraut A, et al.. A receptor pair with an integrated decoy converts pathogen disabling of transcription factors to immunity. Cell. 2015; doi: 10.1016/j.cell.2015.04.025.

29. Maqbool A, Saitoh H, Franceschetti M, Stevenson CEM, Uemura A, Kanzaki H, et al.. Structural basis of pathogen recognition by an integrated HMA domain in a plant NLR immune receptor. Elife. 2015; doi: 10.7554/eLife.08709.

30. Ortiz D, de Guillen K, Césari S, Chalvon V, Gracy J, Padilla A, et al.. Recognition of the *Magnaporthe oryzae* effector AVR-pia by the decoy domain of the rice NLR immune receptor RGA5. Plant Cell. 2017; doi: 10.1105/tpc.16.00435.

31. Barragan AC, Weigel D. Plant NLR diversity: the known unknowns of pan-NLRomes. Plant Cell. 2021; doi: 10.1093/PLCELL/KOAA002.

32. Jia YX, Yuan Y, Zhang Y, Yang S, Zhang X. Extreme expansion of NBS-encoding genes in *Rosaceae*. BMC Genet. 2015; doi: 10.1186/s12863-015-0208-x.

33. Meyers BC, Kozik A, Griego A, Kuang H, Michelmore RW. Genome-wide analysis of NBS-LRR-encoding genes in *Arabidopsis*. Plant Cell. 2003; doi: 10.1105/tpc.009308.

34. Kourelis J, Sakai T, Adachi H, Kamoun S. RefPlantNLR is a comprehensive collection of experimentally validated plant disease resistance proteins from the NLR family. PLoS Biol. 2021; doi: 10.1371/journal.pbio.3001124.

35. Wenger AM, Peluso P, Rowell WJ, Chang PC, Hall RJ, Concepcion GT, et al.. Accurate circular consensus long-read sequencing improves variant detection and assembly of a human genome. Nature Biotechnology. 2019; doi: 10.1038/s41587-019-0217-9.

36. Dumschott K, Schmidt MHW, Chawla HS, Snowdon R, Usadel B. Oxford Nanopore sequencing: new opportunities for plant genomics? J Exp Bot. Oxford Academic; 2020; doi: 10.1093/JXB/ERAA263.

37. Li Q, Jiang XM, Shao ZQ. Genome-Wide Analysis of NLR Disease Resistance Genes in an Updated Reference Genome of Barley. Front Genet. 2021; doi: 10.3389/fgene.2021.694682.

38. Andersen EJ, Ali S, Neil Reese R, Yen Y, Neupane S, Nepal MP. Diversity and evolution of disease resistance genes in barley (*Hordeum vulgare* L.). Evol Bioinform. 2016; doi: 10.4137/EBO.S38085.

39. Habachi-Houimli Y, Khalfallah Y, Mezghani-Khemakhem M, Makni H, Makni M, Bouktila D. Genome-wide identification, characterization, and evolutionary analysis of NBS-encoding resistance genes in barley. 3 Biotech. 2018; doi: 10.1007/S13205-018-1478-6/FIGURES/4.

40. Zhao Y, Huang J, Wang Z, Jing S, Wang Y, Ouyang Y, et al.. Allelic diversity in an NLR gene *BPH9* enables rice to combat planthopper variation. Proc Natl Acad Sci. 2016; doi: 10.1073/PNAS.1614862113/-/DCSUPPLEMENTAL.

41. Lieberman-Aiden E, van Berkum NL, Williams L, Imakaev M, Ragoczy T, Telling A, et al.. Comprehensive mapping of long-range interactions reveals folding principles of the human genome. Science. 2009; doi: 10.1126/science.1178746.

42. Butcher PA, Bell JC, Moran GF. Patterns of genetic diversity and nature of the breeding system in *Melaleuca alternifolia* (Myrtaceae). Aust J Bot. 1992; doi: 10.1071/BT9920365.

43. FindPlantNLRs (2022). https://github.com/ZhenyanLuo/FindPlantNLRs

44. Cheng H, Concepcion GT, Feng X, Zhang H, Li H. Haplotype-resolved de novo assembly using phased assembly graphs with hifiasm. Nat Methods. 2021; doi: 10.1038/s41592-020-01056-5.

45. Durand NC, Shamim MS, Machol I, Rao SSP, Huntley MH, Lander ES, et al.. Juicer Provides a One-Click System for Analyzing Loop-Resolution Hi-C Experiments. Cell Syst. Cell Press; 2016; doi: 10.1016/j.cels.2016.07.002.

46. Durand NC, Shamim MS, Machol I, Rao SSP, Huntley MH, Lander ES, et al.. Juicer Provides a One-Click System for Analyzing Loop-Resolution Hi-C Experiments. Cell Syst. 2016; doi: 10.1016/j.cels.2016.07.002.

47. Dudchenko O, Batra SS, Omer AD, Nyquist SK, Hoeger M, Durand NC, et al.. De novo assembly of the *Aedes aegypti* genome using Hi-C yields chromosome-length scaffolds. Science. 2017; doi: 10.1126/SCIENCE.AAL3327/SUPPL_FILE/DUDCHENKO_SM.PDF.

48. Edwards RJ, Dong C, Park RF, Tobias PA. A phased chromosome-level genome and full mitochondrial sequence for the dikaryotic myrtle rust pathogen, *Austropuccinia psidii*. bioRxiv. 2022; doi: 10.1101/2022.04.22.489119.

49. Chen SH, Rossetto M, Merwe M van der, Lu-Irving P, Yap J-YS, Sauquet H, et al.. Chromosome-level de novo genome assembly of *Telopea speciosissima* (New South Wales waratah) using long-reads, linked-reads and Hi-C. Mol Ecol Resour. 2022; doi: 10.1111/1755-0998.13574.

50. Vurture GW, Sedlazeck FJ, Nattestad M, Underwood CJ, Fang H, Gurtowski J, et al.. GenomeScope: fast reference-free genome profiling from short reads. Bioinformatics. 2017; doi: 10.1093/BIOINFORMATICS/BTX153.

51. Ranallo-Benavidez TR, Jaron KS, Schatz MC. GenomeScope 2.0 and Smudgeplot for reference-free profiling of polyploid genomes. Nat Commun. 2020; doi: 10.1038/s41467-020-14998-3.

52. Tidk (2023). Tidk (Version 0.2.31) https://github.com/tolkit/telomeric-identifier

53. Rhie A, Walenz BP, Koren S, Phillippy AM. Merqury: Reference-free quality, completeness, and phasing assessment for genome assemblies. Genome Biol. 2020; doi: 10.1186/S13059-020-02134-9/FIGURES/6.

54. Simão FA, Waterhouse RM, Ioannidis P, Kriventseva E V., Zdobnov EM. BUSCO: assessing genome assembly and annotation completeness with single-copy orthologs. Bioinformatics. 2015; doi: 10.1093/BIOINFORMATICS/BTV351.

55. Keilwagen J, Hartung F, Grau J. GeMoMa: Homology-Based Gene Prediction Utilizing Intron Position Conservation and RNA-seq Data. Methods Mol Biol. 2019; doi: 10.1007/978-1-4939-9173-0_9.

56. RepeatModeler (2020) RepeatModeler (Version 2.0.1) https://github.com/Dfam-consortium/RepeatModeler

57. Bayer PE, Edwards D, Batley J. Bias in resistance gene prediction due to repeat masking. Nature Plants. 2018; doi: 10.1038/s41477-018-0264-0.

58. Steuernagel B, Witek K, Krattinger SG, Ramirez-Gonzalez RH, Schoonbeek HJ, Yu G, et al.. The NLR-Annotator Tool Enables Annotation of the Intracellular Immune Receptor Repertoire. Plant Physiol. 2020; doi: 10.1104/PP.19.01273.

59. Altschul SF, Gish W, Miller W, Myers EW, Lipman DJ. Basic local alignment search tool. J Mol Biol. 1990; doi: 10.1016/S0022-2836(05)80360-2.

60. Eddy SR. Accelerated Profile HMM Searches. PLoS Comput Biol. 2011; doi: 10.1371/JOURNAL.PCBI.1002195.

61. Thrimawithana AH, Jones D, Hilario E, Grierson E, Ngo HM, Liachko I, et al.. A whole genome assembly of *Leptospermum scoparium* (Myrtaceae) for mānuka research. N Z J Crop Hortic Sci. 2019; doi: 10.1080/01140671.2019.1657911.

62. Christie N, Tobias PA, Naidoo S, Külheim C. The *Eucalyptus grandis* NBS-LRR gene family: Physical clustering and expression hotspots. Front Plant Sci. 2016; doi: 10.3389/fpls.2015.01238.

63. Hoff KJ, Lomsadze A, Borodovsky M, Stanke M. Whole-Genome Annotation with BRAKER. Methods Mol Biol. 2019; doi: 10.1007/978-1-4939-9173-0_5.

64. Jones P, Binns D, Chang HY, Fraser M, Li W, McAnulla C, et al.. InterProScan 5: genome-scale protein function classification. Bioinformatics. 2014; doi: 10.1093/BIOINFORMATICS/BTU031.

65. Sankeymatic (2023) https://github.com/nowthis/sankeymatic

66. Hsieh JF, Chuah A, Patel HR, Sandhu KS, Foley WJ, Kiilheim C. Transcriptome profiling of *Melaleuca quinquenervia* challenged by myrtle rust reveals differences in defence responses among resistant individuals. Phytopathology. 2018; doi: 10.1094/PHYTO-09-17-0307-R.

67. Kim D, Paggi JM, Park C, Bennett C, Salzberg SL. Graph-based genome alignment and genotyping with HISAT2 and HISAT-genotype. Nat Biotechnol. 2019; doi: 10.1038/s41587-019-0201-4.

68. Wang W, Das A, Kainer D, Schalamun M, Morales-Suarez A, Schwessinger B, et al.. The draft nuclear genome assembly of *Eucalyptus pauciflora*: a pipeline for comparing de novo assemblies. Gigascience. 2020; doi: 10.1093/GIGASCIENCE/GIZ160.

69. Morgan HD, Westoby M. The Relationship Between Nuclear DNA Content and Leaf Strategy in Seed Plants. Ann Bot. 2005; doi: 10.1093/AOB/MCI284.

70. Borrelli GM, Mazzucotelli E, Marone D, Crosatti C, Michelotti V, Valè G, et al.. Regulation and Evolution of NLR Genes: A Close Interconnection for Plant Immunity. Int J Mol Sci. 2018; doi: 10.3390/IJMS19061662.

71. Rayamajhi MB, Van TK, Pratt PD, Center TD. Interactive association between *Puccinia psidii* and *Oxyops vitiosa*, two introduced natural enemies of *Melaleuca quinquenervia* in Florida. *Biological Control*. 2006; doi: 10.1016/j.biocontrol.2005.10.013.

72. Chakrabarty S, Hsieh J-F, Chakraborty P, Foley WJ, Külheim C. Evolutionary relationship of the NBS-LRR gene family in *Melaleuca* and *Eucalyptus* (Myrtaceae). Tree Genet Genomes. 2023; doi: 10.1007/S11295-023-01602-0.

73. Chen J, Zhang X, Rathjen JP, Dodds PN. Direct recognition of pathogen effectors by plant NLR immune receptors and downstream signalling. Essays Biochem. 2022; doi: 10.1042/EBC20210072.

74. Krattinger SG, Keller B. Molecular genetics and evolution of disease resistance in cereals. New Phytol. 2016; doi: 10.1111/NPH.14097.

75. Tobias PA, Guest DI, Külheim C, Park RF. De novo transcriptome study identifies candidate genes involved in resistance to *Austropuccinia psidii* (myrtle rust) in *Syzygium luehmannii* (riberry). Phytopathology. 2018; doi: 10.1094/PHYTO-09-17-0298-R.

76. Chen SH; Martino AM; Luo Z; Schwessinger B; Jones A; Tolessa T; Bragg JG; Tobias PA; Edwards RJ: Supporting data for "A high-quality pseudo-phased genome for Melaleuca quinquenervia shows

allelic diversity of NLR-type resistance genes" GigaScience Database. 2023. http://doi.org/10.5524/102460

77. Zhou T, Wang Y, Chen JQ, Araki H, Jing Z, Jiang K, et al.. Genome-wide identification of NBS genes in japonica rice reveals significant expansion of divergent non-TIR NBS-LRR genes. Mol Genet Genomics. 2004; doi: 10.1007/S00438-004-0990-Z/FIGURES/5.

78. Wang L, Zhao L, Zhang X, Zhang Q, Jia Y, Wang G, et al.. Large-scale identification and functional analysis of NLR genes in blast resistance in the Tetep rice genome sequence. Proc Natl Acad Sci. 2019; doi: 10.1073/pnas.1910229116.

79. Peona V, Blom MPK, Xu L, Burri R, Sullivan S, Bunikis I, et al.. Identifying the causes and consequences of assembly gaps using a multiplatform genome assembly of a bird-of-paradise. *Mol Ecol Resour*. 2021; doi: 10.1111/1755-0998.13252.

80. Leister D. Tandem and segmental gene duplication and recombination in the evolution of plant disease resistance genes. Trends Genet. 2004; doi: 10.1016/J.TIG.2004.01.007.

81. McHale LK, Haun WJ, Xu WW, Bhaskar PB, Anderson JE, Hyten DL, et al.. Structural Variants in the Soybean Genome Localize to Clusters of Biotic Stress-Response Genes. Plant Physiol. 2012; doi: 10.1104/PP.112.194605.

82. Césari S, Kanzaki H, Fujiwara T, Bernoux M, Chalvon V, Kawano Y, et al.. The NB-LRR proteins RGA4 and RGA5 interact functionally and physically to confer disease resistance. Embo J. 2014; doi: 10.15252/embj.201487923.

83. Zhai C, Zhang Y, Yao N, Lin F, Liu Z, Dong Z, et al.. Function and Interaction of the Coupled Genes Responsible for *Pik-h* Encoded Rice Blast Resistance. PLoS One. 2014; doi: 10.1371/JOURNAL.PONE.0098067.

84. Narusaka M, Shirasu K, Noutoshi Y, Kubo Y, Shiraishi T, Iwabuchi M, et al.. *RRS1* and *RPS4* provide a dual Resistance-gene system against fungal and bacterial pathogens. Plant J. 2009; doi: 10.1111/J.1365-313X.2009.03949.X.

85. Narusaka M, Kubo Y, Hatakeyama K, Imamura J, Ezura H, Nanasato Y, et al.. Interfamily Transfer of Dual NB-LRR Genes Confers Resistance to Multiple Pathogens. PLoS One. 2013; doi: 10.1371/JOURNAL.PONE.0055954.

86. Hamrigk JL, Godt MJW. Effects of life history traits on genetic diversity in plant species. Philos Trans R Soc Lond B Biol Sci. 1996; doi: 10.1098/RSTB.1996.0112.

87. Xu X, Liu X, Ge S, Jensen JD, Hu F, Li X, et al.. Resequencing 50 accessions of cultivated and wild rice yields markers for identifying agronomically important genes. Nat Biotechnol. 2011; doi: 10.1038/nbt.2050.

88. Shen J, Araki H, Chen L, Chen JQ, Tian D. Unique Evolutionary Mechanism in R-Genes Under the Presence/Absence Polymorphism in *Arabidopsis thaliana*. Genetics. 2006; doi: 10.1534/GENETICS.105.047290.

89. Carpenter SJ, Erickson JM, Lohmann KC, Owen MR, Mcarthur JM, Kennedy WJ, et al.. Fitness costs of R-gene-mediated resistance in *Arabidopsis thaliana*. Nature. 2003; doi: 10.1038/nature01588.

90. Kroj T, Chanclud E, Michel-Romiti C, Grand X, Morel JB. Integration of decoy domains derived from protein targets of pathogen effectors into plant immune receptors is widespread. New Phytol. 2016; doi: 10.1111/NPH.13869.

91. Sarris PF, Duxbury Z, Huh SU, Ma Y, Segonzac C, Sklenar J, et al.. A plant immune receptor detects pathogen effectors that target WRKY transcription factors. Cell. 2015; doi: 10.1016/j.cell.2015.04.024.

92. Marchal C, Zhang J, Zhang P, Fenwick P, Steuernagel B, Adamski NM, et al.. BED-domain-containing immune receptors confer diverse resistance spectra to yellow rust. Nat Plants. 2018; doi: 10.1038/s41477-018-0236-4.

93. Chen C, Jost M, Clark B, Martin M, Matny O, Steffenson BJ, et al.. BED domain-containing NLR from wild barley confers resistance to leaf rust. Plant Biotechnol J. 2021; doi: 10.1111/PBI.13542.

94. Yoshimura S, Yamanouchi U, Katayose Y, Toki S, Wang Z-X, Kono I, et al.. Expression of Xa1, a bacterial blight-resistance gene in rice, is induced by bacterial inoculation. Proc Natl Acad Sci. 1998; doi: 10.1073/pnas.95.4.1663.

95. Das B, Sengupta S, Prasad M, Ghose TK. Genetic diversity of the conserved motifs of six bacterial leaf blight resistance genes in a set of rice landraces. BMC Genetics. 2014; doi: 10.1186/1471-2156-15-82.

96. Read ND, Kellock LJ, Collins TJ, Gundlach AM. Role of topography sensing for infection-structure differentiation in cereal rust fungi. Planta. 1997; doi: 10.1007/s004250050115.

97. Jones A, Torkel C, Stanley D, Nasim J, Borevitz J, Schwessinger B. High-molecular weight DNA extraction, clean-up and size selection for long-read sequencing. PLoS One. 2021; doi: 10.1371/JOURNAL.PONE.0253830.

98. Naim F, Nakasugi K, Crowhurst RN, Hilario E, Zwart AB, Hellens RP, et al.. Advanced engineering of lipid metabolism in *Nicotiana benthamiana* using a draft genome and the V2 viral silencing-suppressor protein. PLoS One. 2012; doi: 10.1371/JOURNAL.PONE.0052717.

99. De Coster W, D'Hert S, Schultz DT, Cruts M, van Broeckhoven C. NanoPack: visualizing and processing long-read sequencing data. Bioinformatics. 2018; doi: 10.1093/BIOINFORMATICS/BTY149.

100. Marçais G, Kingsford C. A fast, lock-free approach for efficient parallel counting of occurrences of k-mers. Bioinformatics. 2011; doi: 10.1093/BIOINFORMATICS/BTR011.

101. Kokot M, Dlugosz M, Deorowicz S. KMC 3: counting and manipulating k-mer statistics. Bioinformatics. 2017; doi: 10.1093/BIOINFORMATICS/BTX304.

102. Li H. Minimap2: pairwise alignment for nucleotide sequences. Bioinformatics. 2018; doi: 10.1093/BIOINFORMATICS/BTY191.

103. Danecek P, Bonfield JK, Liddle J, Marshall J, Ohan V, Pollard MO, et al.. Twelve years of SAMtools and BCFtools. Gigascience. 2021; doi: 10.1093/GIGASCIENCE/GIAB008.

104. Patil I. Visualizations with statistical details: The "ggstatsplot" approach. J Open Source Softw. 2021; doi: 10.21105/joss.03167.

105. Kolmogorov M, Yuan J, Lin Y, Pevzner PA. Assembly of long, error-prone reads using repeat graphs. Nat Biotechnol. 2019; doi: 10.1038/s41587-019-0072-8.

106. HyPo (2020). HyPo (Version 1.0.3) https://github.com/kensung-lab/hypo

107. Snyder MW, Adey A, Kitzman JO, Shendure J. Haplotype-resolved genome sequencing: experimental methods and applications. Nat Rev Genet. 2015; doi: 10.1038/nrg3903.

108. PAFScaff (2021). PAFScaff (Version 0.4.1) https://github.com/slimsuite/pafscaff

109. SynBad (2021). SynBad (Version 0.8.4) https://github.com/slimsuite/synbad

110. DepthCharge (2021). DepthCharge (Version 0.2.0) https://github.com/slimsuite/depthcharge

111. Barrnap (2018). Barrnap (Version 0.9) https://github.com/tseemann/barrnap

112. Lowe TM, Chan PP. tRNAscan-SE On-line: integrating search and context for analysis of transfer RNA genes. Nucleic Acids Res. 2016; doi: 10.1093/NAR/GKW413.

113. Nawrocki EP, Eddy SR. Infernal 1.1: 100-fold faster RNA homology searches. Bioinformatics. 2013; doi: 10.1093/BIOINFORMATICS/BTT509.

114. Tarailo-Graovac M, Chen N. Using RepeatMasker to identify repetitive elements in genomic sequences. Curr Protoc Bioinformatics. 2009; doi: 10.1002/0471250953.BI0410S25.

115. Ferguson S, Jones A, Murray K, Andrew R, Schwessinger B, Borevitz J. Plant genome evolution in the genus *Eucalyptus* driven by structural rearrangements that promote sequence divergence. BioRxiv doi: 10.1101/2023.04.19.537464

116. Feng C, Feng C, Lin X, Liu S, Li Y, Kang M. A chromosome-level genome assembly provides insights into ascorbic acid accumulation and fruit softening in guava (*Psidium guajava*). Plant Biotechnol J. 2021; doi: 10.1111/pbi.13498.

117. Ouadi S, Sierro N, Goepfert S, Bovet L, Glauser G, Vallat A, et al.. The clove (S*yzygium aromaticum)* genome provides insights into the eugenol biosynthesis pathway. Commun Biol. Nature Publishing Group; 2022; doi: 10.1038/s42003-022-03618-z.

118. Thornhill AH, Ho SYW, Külheim C, Crisp MD. Interpreting the modern distribution of Myrtaceae using a dated molecular phylogeny. Mol Phylogenet Evol. 2015; doi: 10.1016/J.YMPEV.2015.07.007.

119. Holub EB. The arms race is ancient history in *Arabidopsis*, the wildflower. Nat Rev Genet. 2001; doi: 10.1038/35080508.

120. Anand L, Rodriguez Lopez CM. ChromoMap: an R package for interactive visualization of multi-omics data and annotation of chromosomes. BMC Bioinformatics. 2022; doi: 10.1186/S12859-021-04556-Z/FIGURES/5.

121. Davey NE, Edwards RJ, Shields DC. The SLiMDisc server: short, linear motif discovery in proteins. *Nucleic Acids Res*. 2007; doi: 10.1093/nar/gkm400.

122. Quinlan AR, Hall IM. BEDTools: a flexible suite of utilities for comparing genomic features. Bioinformatics. 2010; doi: 10.1093/BIOINFORMATICS/BTQ033.

123. Wickham, H. ggplot2: Elegant Graphics for Data Analysis. 2nd Edition. Springer Cham; 2016.

124. Gel B, Serra E. karyoploteR: an R/Bioconductor package to plot customizable genomes displaying arbitrary data. Bioinformatics. 2017; https://doi.org/10.1093/bioinformatics/btx346

125. Sievers F, Higgins DG. Clustal Omega. Curr Protoc Bioinformatics. 2014; doi: 10.1002/0471250953.BI0313S48.

126. Nguyen LT, Schmidt HA, von Haeseler A, Minh BQ. IQ-TREE: A fast and effective stochastic algorithm for estimating maximum-likelihood phylogenies. Mol Biol Evol. 2015; doi: 10.1093/MOLBEV/MSU300.

127. Letunic I, Bork P. Interactive Tree Of Life (iTOL) v5: an online tool for phylogenetic tree display and annotation. Nucleic Acids Res. 2021; doi: 10.1093/NAR/GKAB301.

128. Lechner M, Findeiß S, Steiner L, Marz M, Stadler PF, Prohaska SJ. Proteinortho: Detection of (Co-)orthologs in large-scale analysis. *BMC Bioinformatics*. 2011; doi: 10.1186/1471-2105-12-124.

129. Buchfink B, Reuter K, Drost H-G. Sensitive protein alignments at tree-of-life scale using DIAMOND. *Nat Methods*. Nature Publishing Group; 2021; doi: 10.1038/s41592-021-01101-x.

Figure 1

Click here to access/download;Figure;Figure 1. Distribution.png

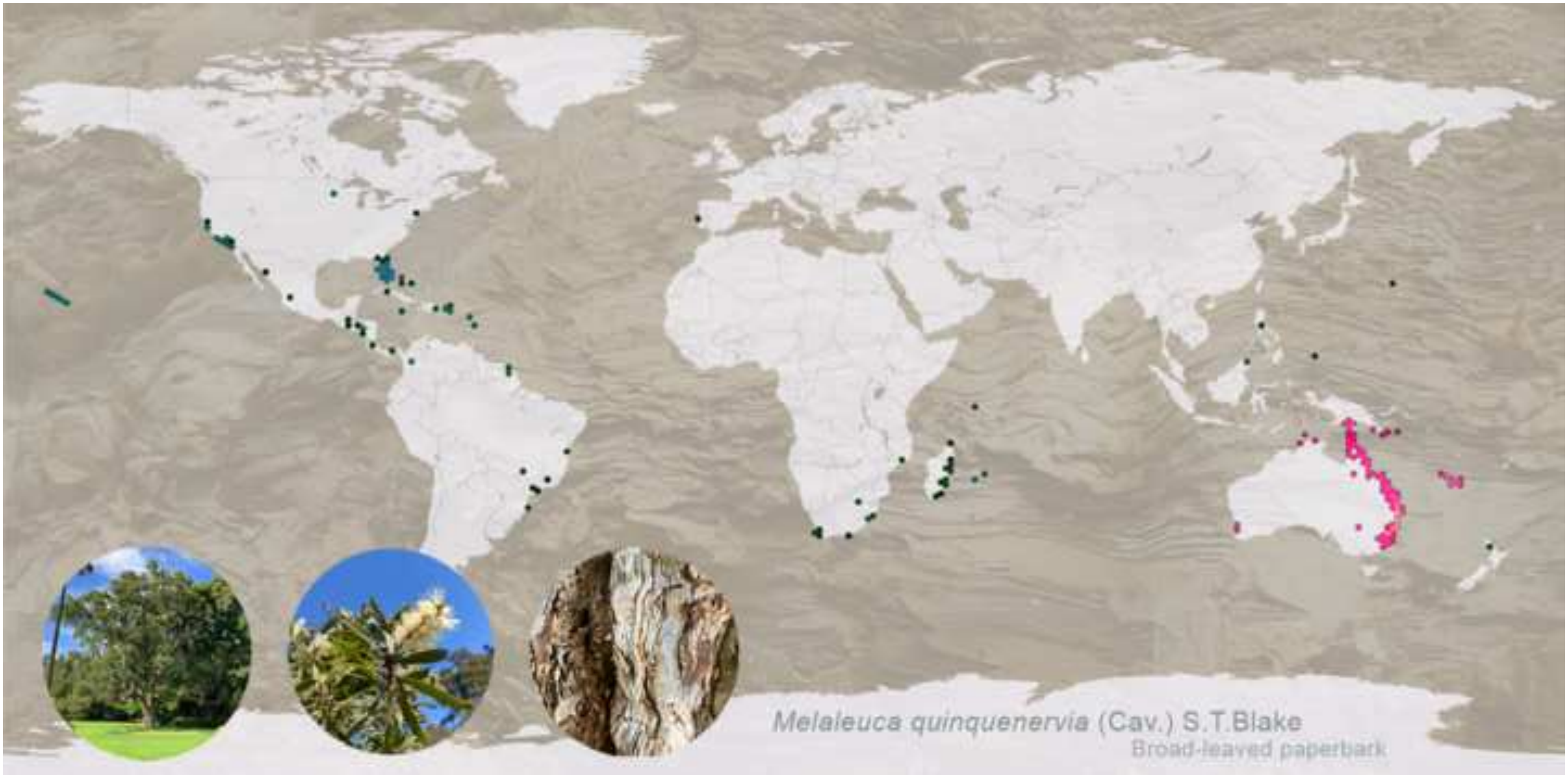

Figure 2

Click here to access/download;Figure;GIGA-D-23-00119-Revision2-Fig2.pdf

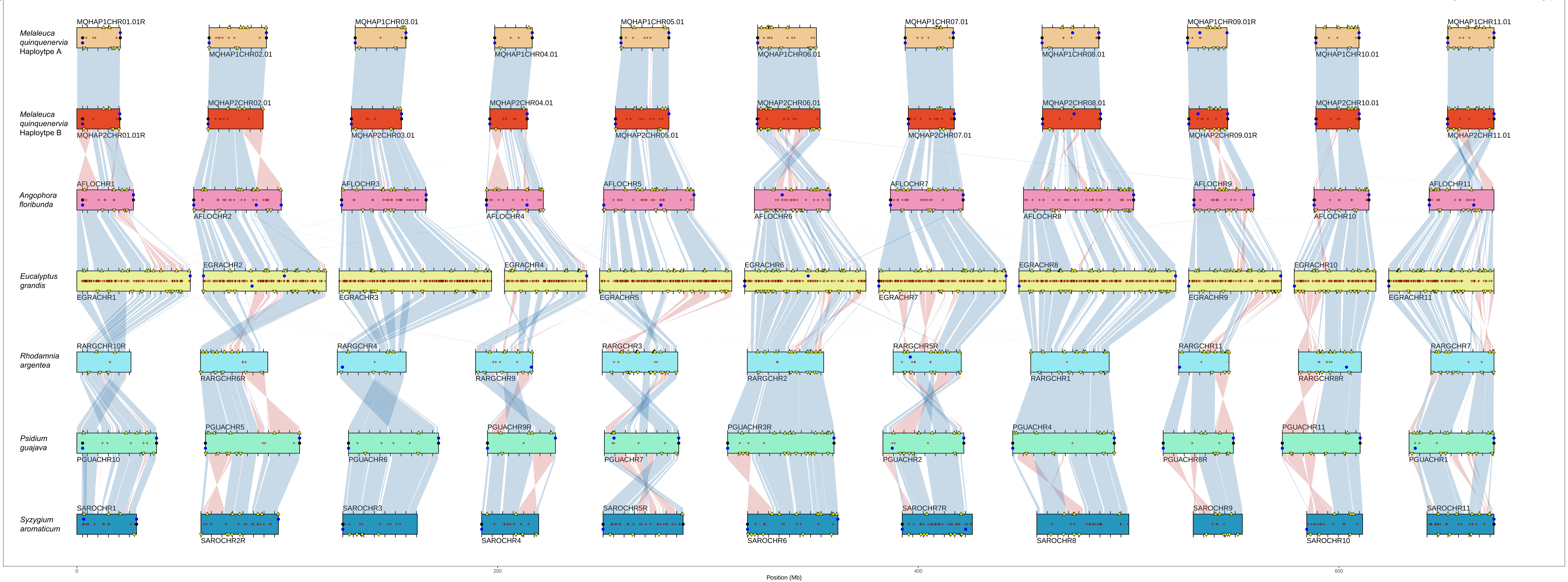

Figure 3

Click here to access/download;Figure;Figure 3.
CN_HiFi_A_B.pdf

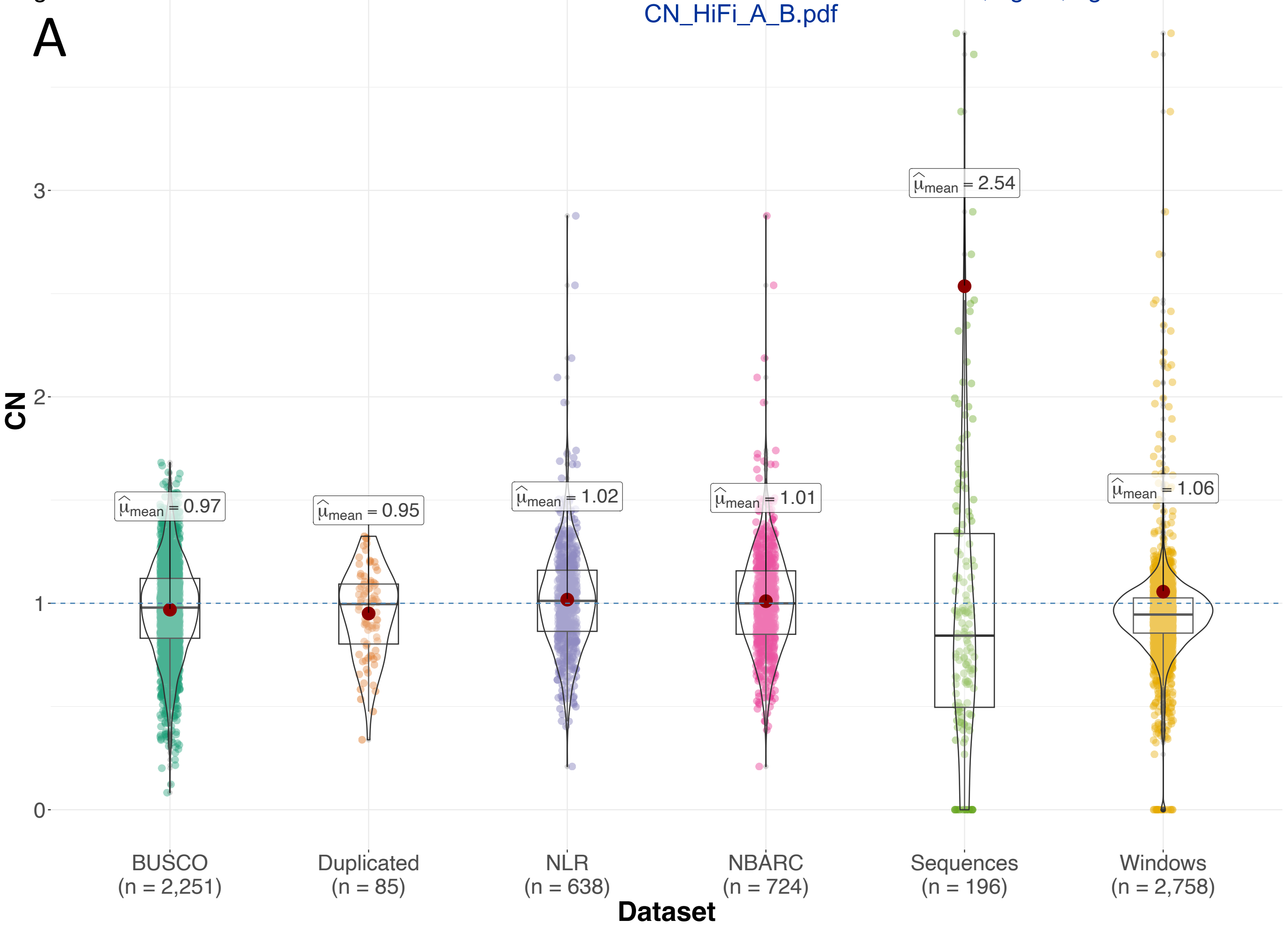

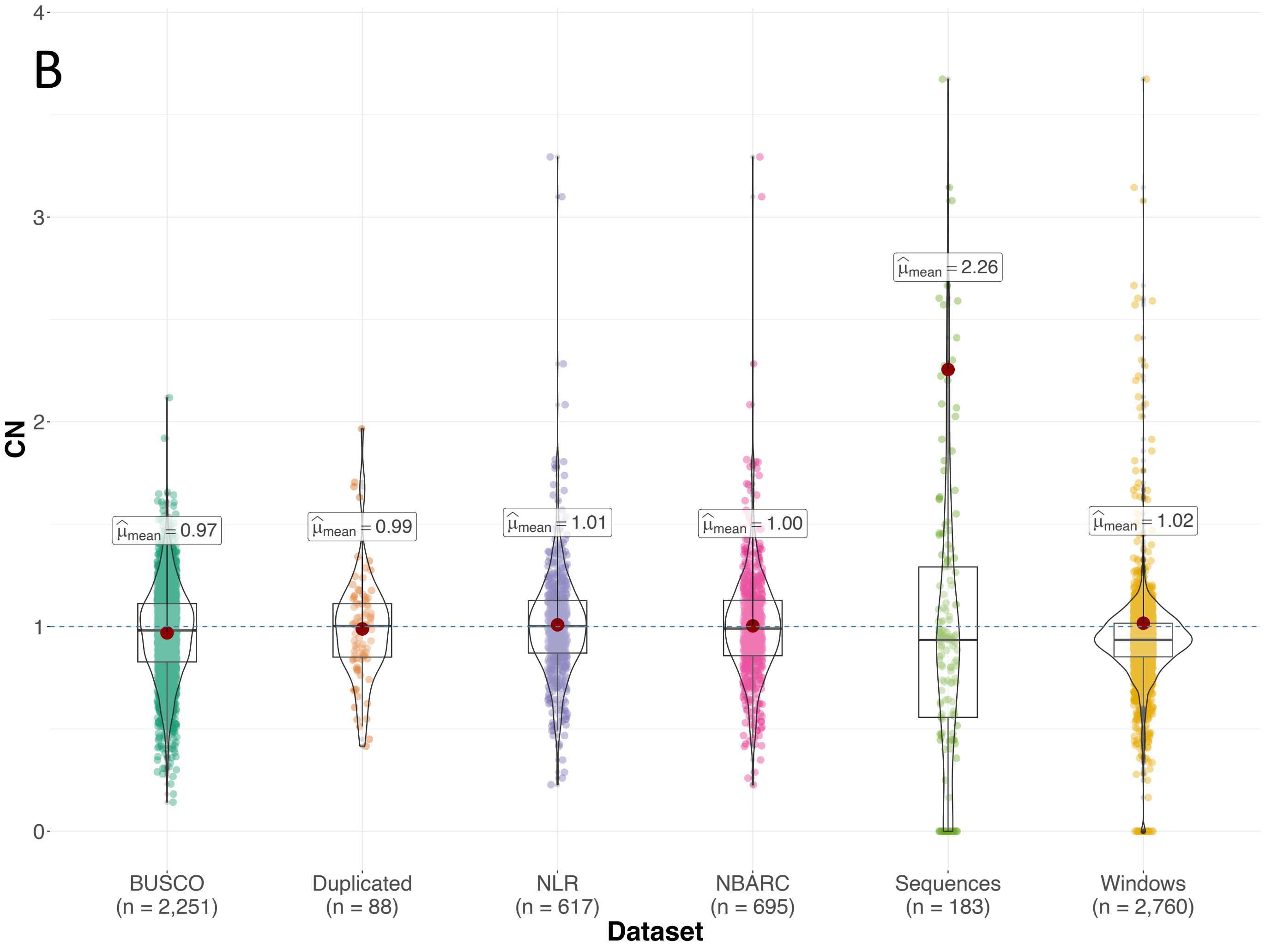

Figure 4

Click here to access/download;Figure;GIGA-D-23-00119-Revision2-Fig4.png

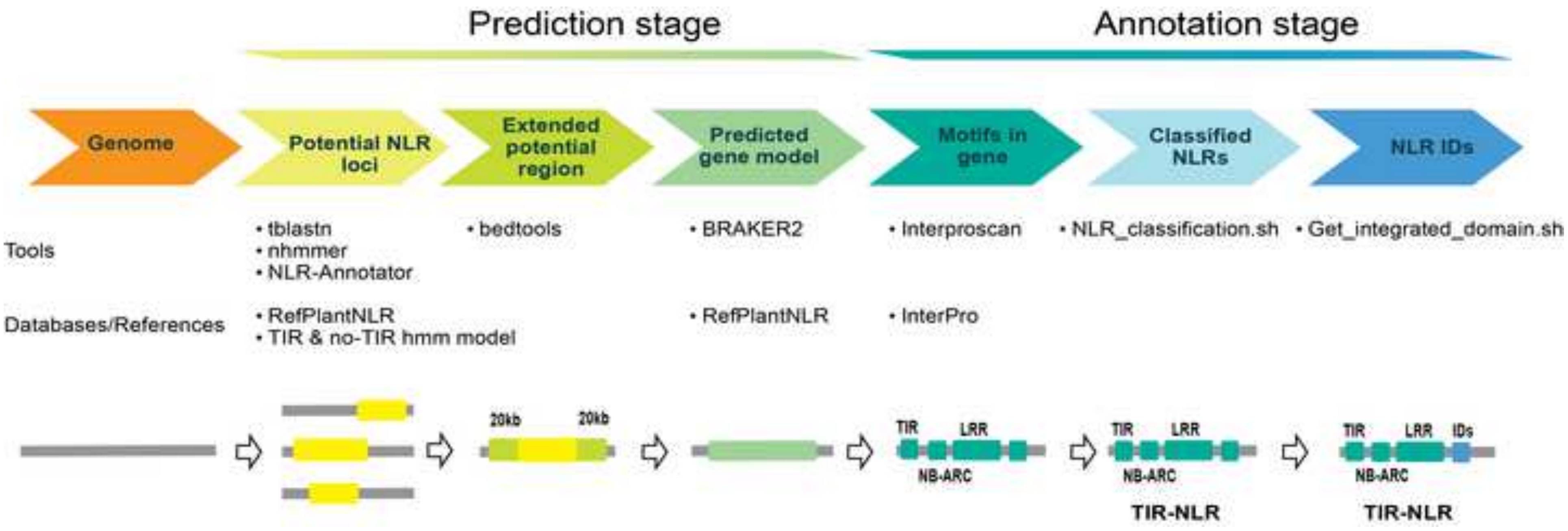

Figure 5

Click here to access/download;Figure;GIGA-D-23-00119-Revision2-Fig5.png

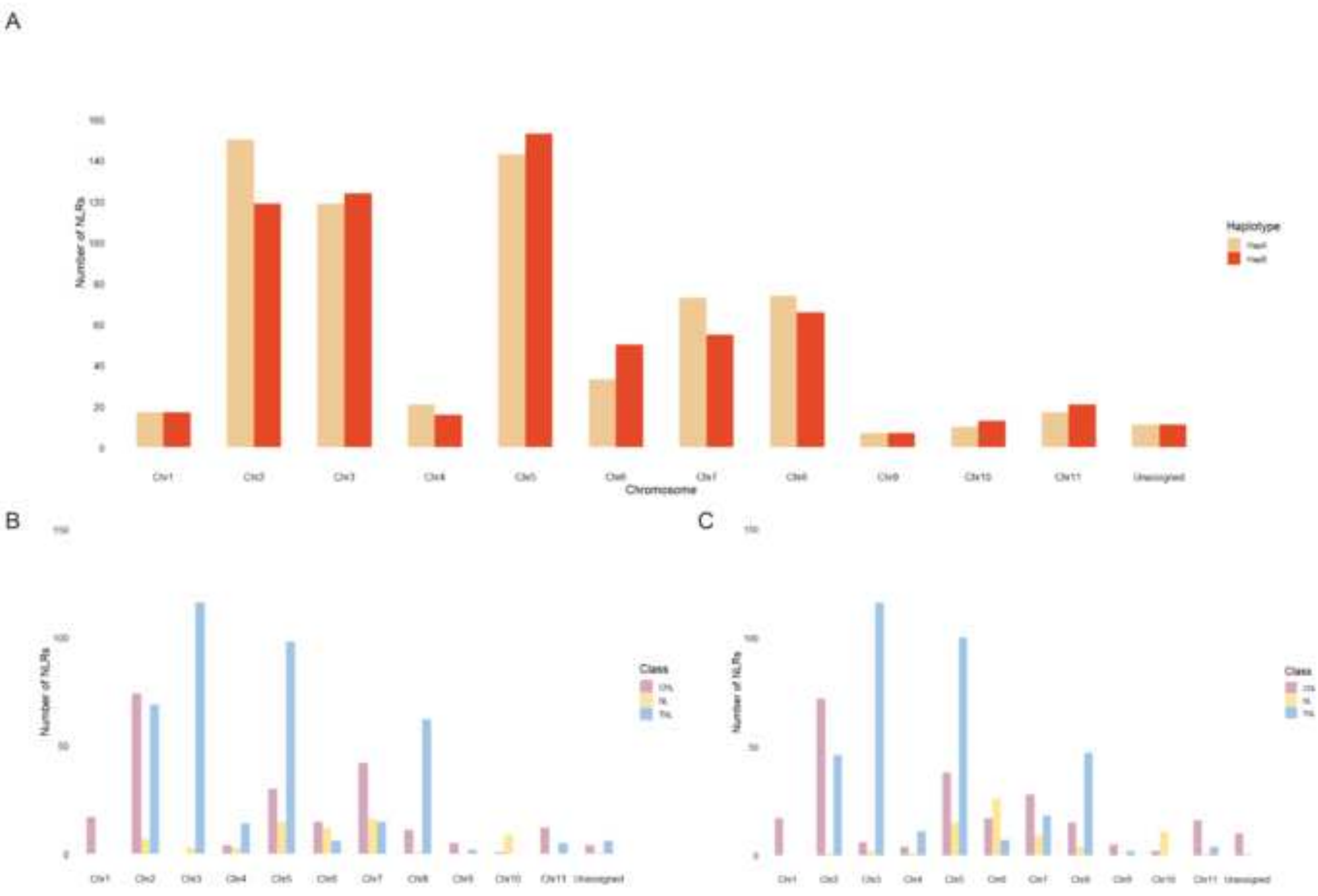

Figure 6

Click here to access/download;Figure;GIGA-D-23-00119-Revision2-Fig6.png

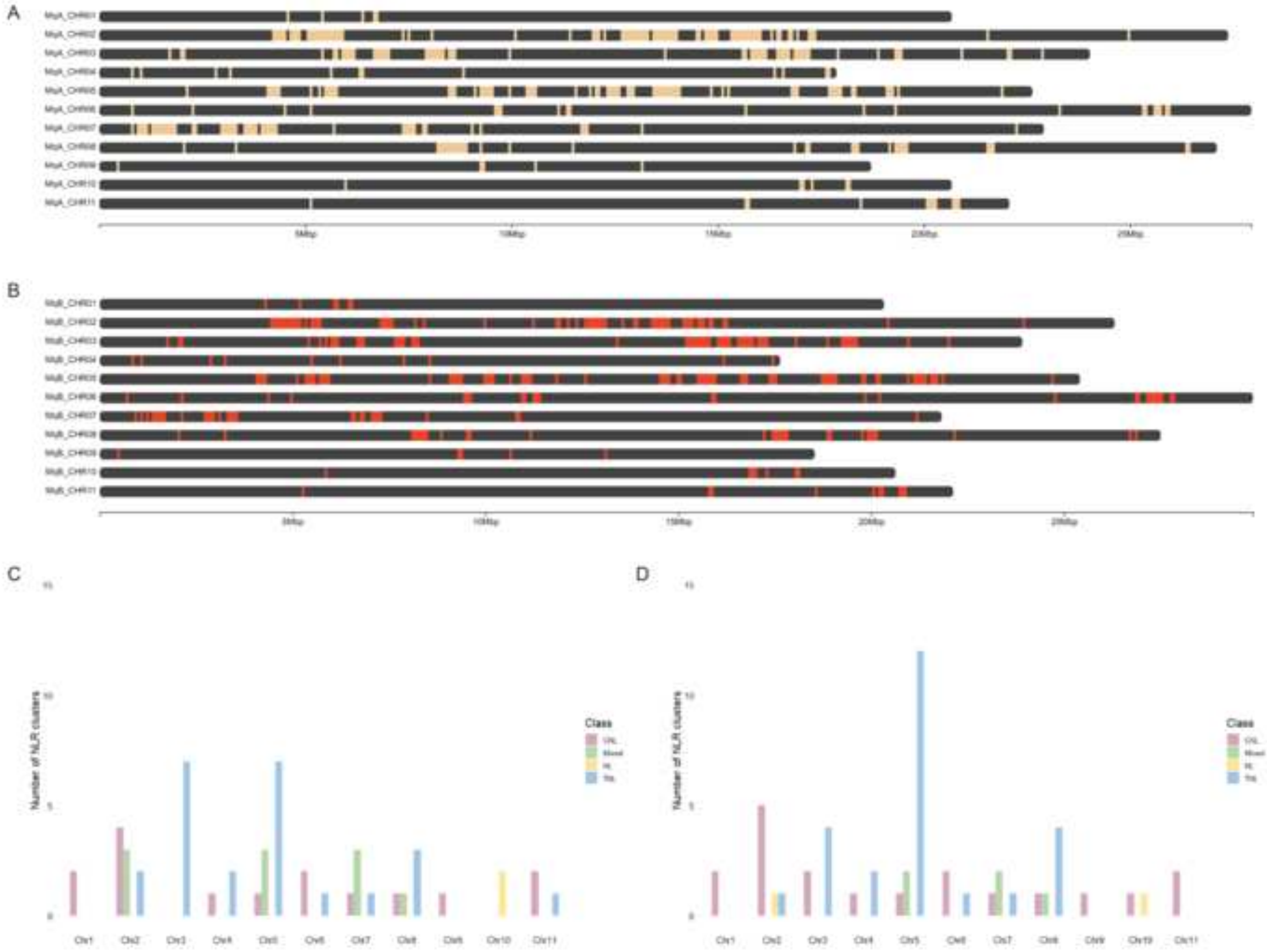

Figure 7

Click here to access/download;Figure;GIGA-D-23-00119-Revision2-Fig7.png

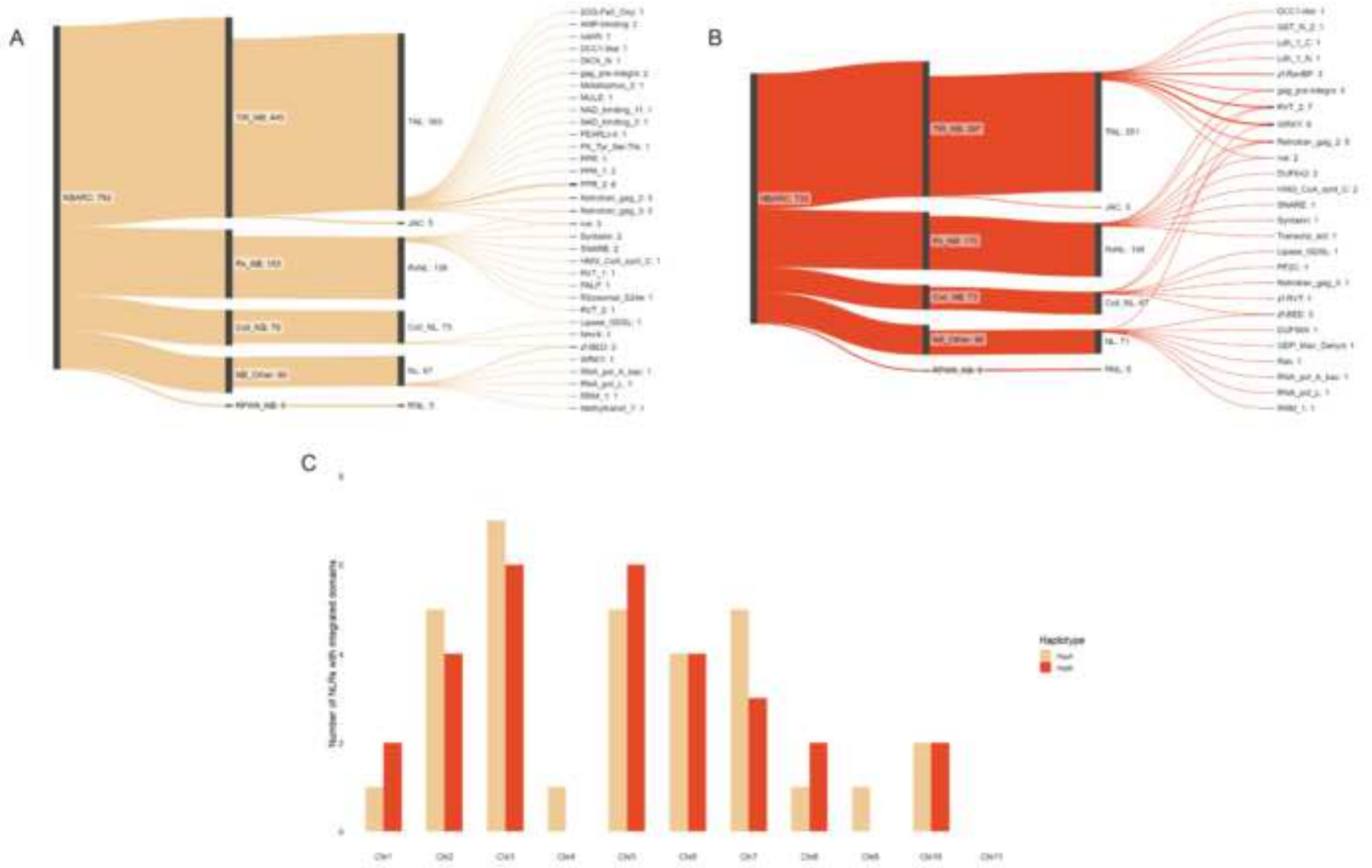

Figure 8

Click here to access/download;Figure;Figure 8. Phylogenetic tree.png

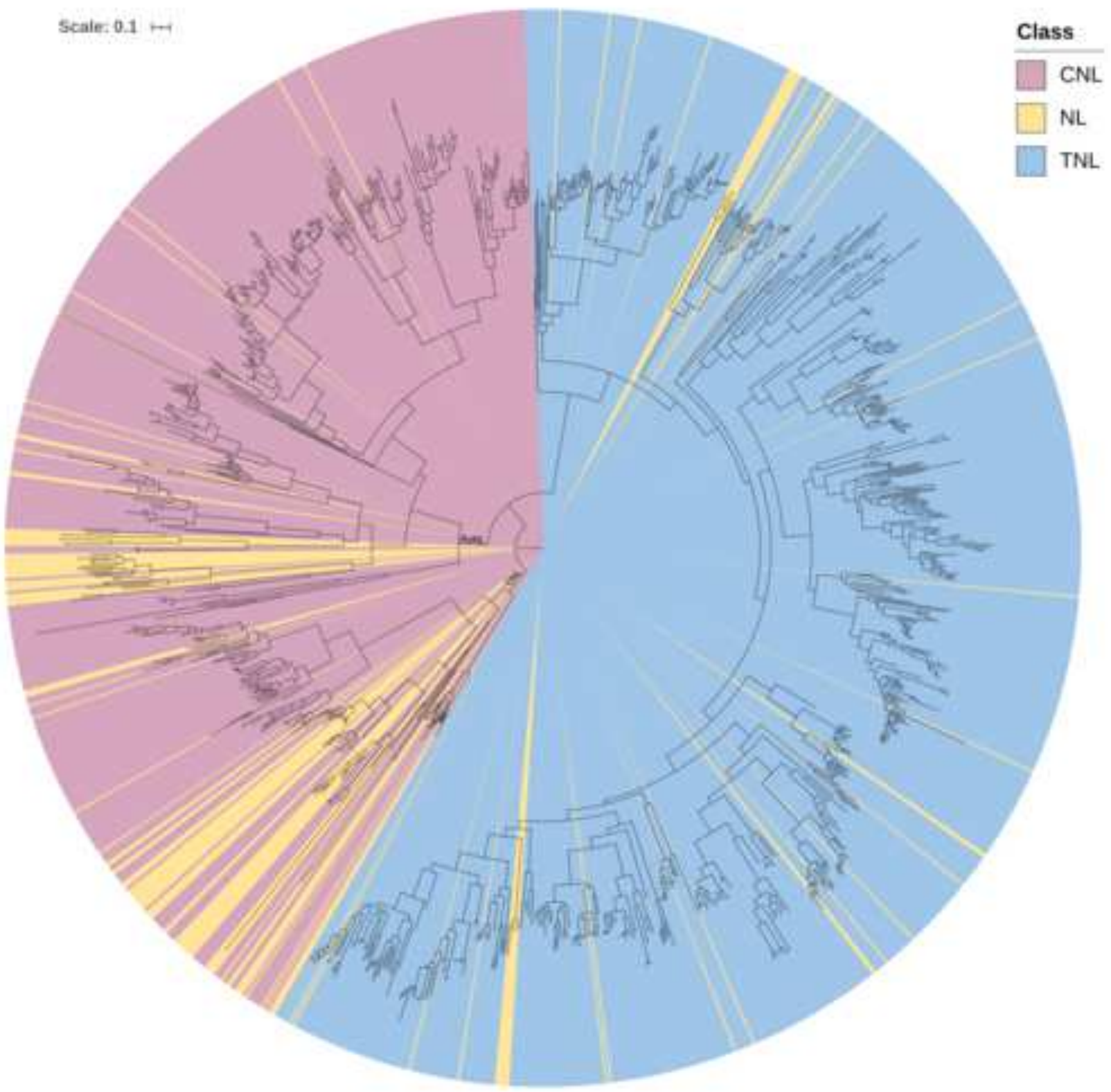

Click here to access/download
Supplementary Material
GIGA-D-23-00119-Revision-Supplementary.xlsx

Personal Cover

Click here to access/download;Personal Cover;GIGA-D-23-00119-Response2.docx

THE UNIVERSITY OF WESTERN AUSTRALIA

UWA Oceans Institute

28 Sep 2023

Nicole Nogoy
Editor
GigaScience

Dear Dr Nogoy

RE: Response to Reviewer Comments - GIGA-D-23-00119

Thank you for the opportunity to submit an updated revision to our manuscript, "A high-quality pseudo-phased genome for Melaleuca quinquenervia shows allelic diversity of NLR-type resistance genes" (GIGA-D-23-00119). We appreciate the attention to detail of the reviews and have made the requested revisions. These are listed in detail, below, with reviewer's comments in black and responses in blue. New text, where appropriate, has been provided in red. The updated manuscript has been uploaded in two versions: with and without changes highlighted in red.

Yours sincerely,

Dr. Richard Edwards

Laboratory Lead, Ocean Genomes Laboratory
Minderoo OceanOmics Centre at UWA
UWA Oceans Institute

Adjunct Associate Professor in Genomics and Bioinformatics
School of Biotechnology and Biomolecular Sciences
UNSW Sydney

The University of Western Australia
M470, 64 Fairway, Crawley WA 6009 Australia

T +61 8644 3142 M +61 405 753 695 E rich.edwards@uwa.edu.au

CRICOS Provider Code 00126G

Reviewer #1

Thank you for sharing a revised version of the manuscript - it's clear that a lot of work was put into making the changes. I believe that most of my concerns were addressed and am confident that my additional comments can be addressed without another round of review.

Main Text:

Specific

236 - "NLRs require NB and LRR to be functional" - this is not true, the variation observed in NLR modules is described in Monteiro and Nishimura Annual Reviews 2018.
A solution could be to slightly change to say "canonical NLRs require…"

Added "canonical" as suggested.

248 - please replace "genes" with "NLR genes" to avoid confusion

Added "NLR" as suggested.

295 - delete "novel" - did you check to see if any of your predicted domain coordinates overlap each other? I've seen this in the past and it can lead to artificially high counts. Consider removing the percentages to avoid misleading (unless you did check).

Novel has been removed and for clarity the percentages changed to now reflect those NLRs containing more than one unique integrated domain.

398 - I would consider replacing "no natural enemies" with "where it is exposed to a new suite of microbes" or similar.

Replaced no natural enemies with the suggested text.

417 - I think a more general statement here is appropriate - "this domain may play a role in NLR function"

Made statement more general as suggested.

431 - I appreciate the comment about full-length NLRs, but it seems out of place here. I still think it belongs in "Novel pipeline to identify and classify NLRs" section of the paper.

We have removed the comment of the full-length NLRs from line 412 and added the comment in lines 221 – 223.

Figures:

General
The figures that use a unified color palette for haplotype A and B are easier to interpret - consider extending this to all figures. For example, Figure 2 and Figure 6 use different colors than the oranges used in other figures.

The colours used for Hap A and B in Figure 2 have been changed to match the colours used in other figures. The palette used for the other species in the synteny plot have also been updated to complement and be easy to distinguish from the Hap A and B colours.

Figure 6 has been amended to reflect the colours used throughout the other figures for consistency.

Figures need more informative axes labels.

Axes labels have been updated for Figures 5 – 7 for clarity.

Specific
Figure 2 - adding the full species name would help readability, especially for those not intimately familiar with these species

The full scientific name for each species has been added to the figure.

Figure 4 - it would be helpful to add a label stating how much sequence is added to the NLR loci (20kb)

This has been added to the figure.

Figure 5 and 6 - Consider merging C and D so that all data hapA and B for each chromosome are grouped - the important comparison here seems to be 'how are the counts on chromosomes different across the two haplotypes'.

While I agree part of the focus is the differences between haplotypes, our preference would be to keep these figures as they stand. Merging the graph into differences between haplotypes may reduce the visual representation of the large number of the NLRs that exist within the *M. quinquenervia* genome.

Figure 6 legend - "D and E" referenced in legend, no D and E in current iteration of figure

This has been corrected.

Figure 7 legend - please replace "(Ref)" with appropriate reference. Add to legend that the count of each domain is included. *I really like this figure

Reference added and legend amended to include details of the count of each domain is included. Thank you, this is one of our favourite figures too!
