# Supplementary material for: A high-quality pseudo-phased genome for Melaleuca quinquenervia shows allelic diversity of NLR-type resistance genes
Source: Gigascience. 2023 Dec 14;12:giad102. doi: 10.1093/gigascience/giad102 (PMC10720953; doi:10.1093/gigascience/giad102)
Supplement: giad102_Response_to_Reviewer_Comments_Original_Submission [file giad102_response_to_reviewer_comments_original_submission.pdf]

# Author's Response To Reviewer Comments

Please see the attached Covering letter for a formatted version of responses.

RE: Response to Reviewer Comments - GIGA-D-23-00119
Thank you for the opportunity to submit a revised version of our manuscript, "A high-quality pseudo-phased genome for Melaleuca quinquenervia shows allelic diversity of NLR-type resistance genes" (GIGA-D-23-00119). We appreciate the thoughtful reviews and have made a number of revisions that we think improve the manuscript. These are listed in detail, below. The updated manuscript has been uploaded in two versions: with and without changes highlighted in red.

We trust that these revisions are sufficient to satisfy the essential revisions requested for publication. Please let me know if any additional revisions are required.

Reviewer #1 – Andrew Read – University of Minnesota
In the manuscript, A high-quality pseudo-phased genome for Melaleuca quinquenervia shows allelic diversity of NLR-type resistance genes, the authors assemble and analyze a phased genome of a long-lived tree species. In addition to providing a phased genomic resource for an important species, the authors analyze and compare the NLR gene complement in each of the two diploid genomes. I was surprised by the level of diversity of NLR genes in the two copies of the genome (this may be due to my biases based on working in highly homozygous species). This level of within-individual diversity has been largely overlooked by researchers owing to the difficulties of sequencing, assembly, and NLR identification. To address NLR identification, the authors publish a very nice pipeline that combines available tools into a framework that makes a lot of sense to me and will be valuable to anyone doing NLR gene work on new or existing genome assemblies. My main concern comes from not knowing how sequencing gaps and NLRs correlate across the two diploid genomes. Other than this, I think it's a very nice paper that adds to the growing catalog of NLR gene diversity by tackling the challenge of NLRs in a heterozygous genome.
- We thank the reviewer for the encouragement to look a bit closer at the possible impact of assembly gaps. Whilst we tried to address this to some extent with the DepthKopy analysis, which shows that the number of NLR genes in each haplotype is likely to reflect the true number, there remains the possibility of errors within individual clusters that balance out. To address this, we have added an additional analysis where we have predicted the closest orthologue in the other haplotype for each gene, and plotted these along with the position of assembly gaps (Figures S6 and S7), Methods, lines 689-697:
"To identify orthologs, we aligned sister chromosomes of Melaleuca quinquenervia with minimap2 (2.24-r1122) [101] with -cx asm20 and alignments were filtered with 'length ≥1000bp and identity ≥90%'. We used GOPHER (v3.5.4) [117] to determine orthologs between haplotypes with default settings and used Bedtools intersect (2.27.1) [118] to identify NLRs which located in unaligned regions. Dot plots were generated with ggplot2 (3.4.2) [119]. Syntenic graphs were generated with KaryoploteR (1.26.0) [120] with nucleotide aligned regions from minimap2 (2.24-r1122) [101]. Gaps in the assembly were rated as either Syntenic (both sides map in the correct order and orientation to the alternative haplotype), or non-syntenic (mismatched best-matching scaffolds from the alternative haplotype for each side of the gap) using SynBad ratings [108]."
Details are included in the response to specific questions, below.

Many of the authors' interesting observations are based on comparisons of NLRs on the two haploid genomes, however some things are not clear to me:
1. Do any predicted NLR-genes overlap gaps in the alternative haploid genome?
- Some of the larger clusters of NLRs overlap gaps in the other haplotype. This is not atypical of challenging regions to assemble due to repetitive regions. However, there are no obvious indications that an apparent expansion in one haplotype is due to missing genes in the other. We have added some additional text in the Results (L296-300):
"To investigate the role of assembly quality and completeness on NLR identification and clustering, we identified the closest ortholog in the other haplotype for each NLR gene, and plotted these relationships along with the positions of assembly gaps (Figure S6, S7). Whilst a few NLR clusters had assembly gaps in one or other haplotype, there were no obvious cases where a haplotype-specific expansion could be

explained by a gap corresponding to the homologous region (Figure S7, S8)."
And in the Discussion (L491-497):
"Closer inspection of NLR clusters revealed that some of the larger clusters overlapped with genome assembly gaps (Figure S6, S7). As NLRs are highly repetitive, this may be the result of challenged associated with assembling highly repetitive genomic regions. This has been observed for other multi-copy repetitive gene families such as the major histocompatibility complex family [78]. Nevertheless, the majority of NLRs are present at a read-depth consistent with correct copy numbers (Figure 3, S4 and S5), indicating that assembly difficulties in NLR repeats has not substantially affected results."

2. If there is a predicted NLR-gene in one haploid genome and not the alternative genome, what is at the locus? Is it a structural variant indicating insertion/deletion of the NLR or is there 'NLR-like' sequence there that just didn't pass the pipeline filters indicating an NLR fossil (or similar) – to me this is an important distinction.
- Whilst we think this is a fascinating question, it is beyond the scope of this paper to do a detailed alignment and deep dive into the evolutionary dynamics of the NLR genes themselves. However, we agree that this might be important in some cases and have thus added some additional resources to help the reader and provide additional context (see above). L406-408:
"We estimated 125 genes in Haplotype A had no ortholog in the alternate Haplotype, while 107 from Haplotype B had no ortholog in the alternate Haplotype (Figure S6, S7)."
Most of these cases occur in larger NLR clusters and the alternative haplotype appears to contain a non-orthologous NLR. This could be a feature of the diversity of these genes (see also response to Q3, below), but we also acknowledge that it could arise from insufficient power in orthology detection, L409-412:
"As such, analysis of orthologs between haplotypes is limited to currently available software which is designed to compare species. The software limitation may therefore lead to some discrepancies in ortholog numbers within our analyses (Figure S6, S7)."

3. How many of the NLR-genes on the two haploid genomes cluster 1:1 with their homolog on the alternative haploid genome – I'm particularly interested in the 15 'mismatched' N-term-NBARC examples. It would be nice to know if these have partners in the alternative haploid genome, and if the partner has the same mismatch (if not, it would support the proposed domain swapping story)
I believe each of these concerns will require whole genome alignment of the two haploid genomes.
- We thank the reviewer for this suggestion and agree that a whole genome alignment of the two haploid genomes would be interesting. However, given the repetitive nature of the NLR clusters and the observations of differences in gene content, we were concerned that whole genome alignment would be more prone to bias and errors than the all-by-all pairwise alignment approach of NLR genes that we have taken. The majority of NLR genes cluster with their closest orthologue on the alternative haplotype (see above). As suggested, we have taken a closer look at the 15 mismatched/N-terminal domain swapped NLRs.
Methods, L683-685: "To investigate the homologues of the 15 NLRs containing mismatched N-terminal and NBARC domains, we ran ProteinOrtho (v6.0.15) [124] on the NLRs used for phylogenetic analysis with BLASTP run using DIAMOND (v2.1.6) [125]."
Discussion, L452-L463: "Two of these NLRs have homologues in the alternative haplotype lacking an N-terminal domain, and a one is homologous to a TNL gene. A further five have no homologous partner in the alternative haplotype, with the remaining seven homologous to the NLRs with swapped domains. These results suggest amino terminal domain swapping as a possible evolutionary mechanism, however further functional and molecular validation is required."

Additional comments (by line where indicated)
The authors introduce the idea that M. quinquenervia is invasive in Florida, but this thread is never followed up on in the discussion and makes it feel a bit awkward. It would help if the authors clarified how the genome could help with management in native and invasive ranges.
- Added to Discussion 'Analysis of gene families such as NLRs may also assist in understanding how invasive species manage to escape native-range microbes, as is the case for M. quinquenervia in Florida where it has no natural enemies [70]' at lines 394 – 396 in the discussion:
"Analysis of gene families such as NLRs may also assist in understanding how invasive species manage to escape native-range microbes, as is the case for M. quinquenervia in Florida where it has no natural enemies [71]."

Could the authors add some context for why ONT data was included and how it was used?
- This was explained in the Analyses section L169-170:
"To independently verify the HiFi assemblies, we assembled and scaffolded the ONT data (Figure S1C and D) which showed a high degree of synteny to the HiFi assemblies (Figure S2A and B)."
And Discussion, L374-375:
"Additionally, the genome and subsequent analyses were independently validated with scaffolded assemblies using ~234x ONT data."

It would be helpful if the authors provided a weblink to the iTOL tree
- A link was added and the Newick tree file is available on GigaDB.

164-166 – The observation of inversions potentially caused by assembly errors is nice!
- No change needed.

206 – add reference: Bayer PE, Edwards D, Batley J (2018) Bias in resistance gene prediction due to repeat masking. Nat Plants 4: 762–765. pmid:30287950
- Reference has been added.

240-246 – I'm not sure about excluding these incomplete NLRs – it would be interesting and potentially informative to see where they cluster (do they cluster with an NLR from the alternative haplotype? If so it may indicate truncation of one copy, etc) – however, if the author's wish to remove these at this step I think they can add a statement like "we were interested in full-length NLRs, the filtered incomplete NLRs may represent…."
- We have added a clarifying statement (Discussion L431): "While we were interested in analysing full-length NLRs, …"
And provided the additional data (L439-440): "Chromosomal locations for all truncated NLRs are available in GigaDB."

429-430 – The criteria used to define clusters is described in the methods, can you confirm (and mention) that this is the same as used in the analyses you're comparing to for E. grandis, rice, and Arabidopsis.
- Clarified (L465): "…, employing the same method for determining clusters."

435-437 – I'm interested to know if the four heterogenous clusters contain any of the N-term domain-swapped NLRs
- Yes. In Haplotype A, all of the N-terminal domain swapped NLRs are from within a single heterogenous cluster on Chromosome 5. Of the 9 genes within this cluster, 5 are the domain swapped NLRs with the remaining NLRs are two TNLs, one CNL, and one NLR. This is also observed in Haplotype B where most of the domain swapped NLRs are from a single Heterogenous cluster on Chromosome 5. Although not clustering, all but one of the remaining domain swapped NLRs from Haplotype B are in heterogenous clusters. We hope that this will be clearer with the additional plots and tables, along with any other similar questions that the reader might have.

479-480 – The zf-BED domain is also present in rice NLRs – include citation for Xa1/Xo1
- Added in-text citation for the reference:
Yoshimura S, Yamanouchi U, Katayose Y, Toki S, Wang Z-X, Kono I, et al.. Expression of Xa1, a bacterial blight-resistance gene in rice, is induced by bacterial inoculation. Proc Natl Acad Sci. 1998; doi: 10.1073/pnas.95.4.1663.

523-524 – can you specify which base-call model was used on the ONT data?
- Added (L568): "(model_version_id=2021-05-05_dna_r9.4.1_promethion_768_922a514b)"

I'm curious about the presence/absence of IDs in the analyzed NLRs and would be very curious to know if the authors observe syntenic homologs across the two haploid genomes with ID presence/absence or presence of different IDs polymorphisms.
- We have incorporated additional analyses into Supplementary Table 4 which has a full list of integrated domain containing NLRs. In short, we do observe syntenic homologs across the haploid genomes with

presence/absence. We also observe different ID polymorphisms where for example there are multiple copies of the integrated domain in one NLR and only one copy in its homolog in the alternative haplotype.

Reviewer #2

The manuscript about NLR-type resistance genes in two haplotypes of Melaleuca quinquenervia is a relevant contribution to the research of Myrtaceae genomes and other long-lived trees.
The methods are well described and should be reproducible with the available information and raw data, provided the authors mentioned all non-default settings in the method section. The FindPlantNLRs pipeline seems to be well documented on github.

I believe that this manuscript is ready for publication after some small changes. Page and line numbers in the comments below refer to the PDF document:

1. The quality of some figures is not good (even upon download and zoom into the plot) and should be improved to higher resolution for publication. Especially in figure 3, all labels are too pixelated and hard to read. I would also recommend an increase in text size for this figure. In Figure 6 D & E, the authors should consider using consistent text sizes on the axes, and even though the quality is acceptable, a higher resolution of the labels would still be better.
- Figure 3 has been regenerated with larger labels and at a higher resolution. White backgrounds have been added to figures 5-8 to improve readability.

2. p. 10, Table 2: Although it is a standard statistic for genome assemblies, it would be helpful for some readers to specify what N50 and L50 are.
- We have added footnotes to Table 2 to explain these fields:
† At least half of the bases occur in a contig/scaffold of N50 bp or greater.
‡ L50 is the number of contigs/scaffolds of length N50 bp or greater.

3. p. 19, line 436: I believe the authors are referring to the wrong figure number.
- This has been fixed to reflect the correct figure number.

Below are some additional comments regarding typos or other language issues. While the text is generally well written, I would appreciate commas in certain sentences to improve readability, and think that some nouns are missing articles. I hope the authors will read through their text again and add articles where required, I won't point them out individually.
p.4, line 33: wide range of
p.7, line 130: 'a' instead of 8?
p. 8, line 177: genome
p.12, line 250: chromosome 2, add comma before 'while' in next line
p.12, line 253: on all other chromosomes?
p. 13, line 271: to occur?
p.16, line 347: remove 'and'
p.17, line 382, 384: orthologs?
p.20, line 469: 'lead to the triggering of defence response' rephrase to make sense with the previous half of the sentence, also, defence response should have an article
p.20, line 489/490: missing word?
- Typos pointed out above have been fixed. Additionally, multiple authors have edited the manuscript to improve clarity and cohesion.

Comments from GigaDB editor:

Furthermore, your manuscript states (line 568) that you used BUSCO V5.1.2 whereas the version uploaded is 5.3.0. I presume V5.3.0 is the correct value so this will also need to be updated in your manuscript.
- Amended BUSCO version in methods to v5.3.0.
