# Supplementary material for: A high-quality pseudo-phased genome for Melaleuca quinquenervia shows allelic diversity of NLR-type resistance genes
Source: Gigascience. 2023 Dec 14;12:giad102. doi: 10.1093/gigascience/giad102 (PMC10720953; doi:10.1093/gigascience/giad102)
Supplement: giad102_Response_to_Reviewer_Comments_Revision_1 [file giad102_response_to_reviewer_comments_revision_1.pdf]

Author's Response To Reviewer Comments

Please see attached covering letter for a formatted version.

Dear Dr Nogoy
RE: Response to Reviewer Comments - GIGA-D-23-00119

Thank you for the opportunity to submit an updated revision to our manuscript, "A high-quality pseudo-phased genome for Melaleuca quinquenervia shows allelic diversity of NLR-type resistance genes" (GIGA-D-23-00119). We appreciate the attention to detail of the reviews and have made the requested revisions. These are listed in detail, below. The updated manuscript has been uploaded in two versions: with and without changes highlighted in red.

Yours sincerely,

Dr. Richard Edwards
Laboratory Lead, Ocean Genomes Laboratory
Minderoo OceanOmics Centre at UWA
UWA Oceans Institute
Adjunct Associate Professor in Genomics and Bioinformatics
School of Biotechnology and Biomolecular Sciences
UNSW Sydney

Reviewer #1
Thank you for sharing a revised version of the manuscript - it's clear that a lot of work was put into making the changes. I believe that most of my concerns were addressed and am confident that my additional comments can be addressed without another round of review.
Main Text:
Specific
236 - "NLRs require NB and LRR to be functional" - this is not true, the variation observed in NLR modules is described in Monteiro and Nishimura Annual Reviews 2018.
A solution could be to slightly change to say "canonical NLRs require…"
Added "canonical" as suggested.

248 - please replace "genes" with "NLR genes" to avoid confusion
Added "NLR" as suggested.

295 - delete "novel" - did you check to see if any of your predicted domain coordinates overlap each other? I've seen this in the past and it can lead to artificially high counts. Consider removing the percentages to avoid misleading (unless you did check).
Novel has been removed and for clarity the percentages changed to now reflect those NLRs containing more than one unique integrated domain.

398 - I would consider replacing "no natural enemies" with "where it is exposed to a new suite of microbes" or similar.
Replaced no natural enemies with the suggested text.

417 - I think a more general statement here is appropriate - "this domain may play a role in NLR function"
Made statement more general as suggested.

431 - I appreciate the comment about full-length NLRs, but it seems out of place here. I still think it belongs in "Novel pipeline to identify and classify NLRs" section of the paper.

We have removed the comment of the full-length NLRs from line 412 and added the comment in lines 221 – 223.

Figures:

General
The figures that use a unified color palette for haplotype A and B are easier to interpret - consider extending this to all figures. For example, Figure 2 and Figure 6 use different colors than the oranges used in other figures.
The colours used for Hap A and B in Figure 2 have been changed to match the colours used in other figures. The palette used for the other species in the synteny plot have also been updated to complement and be easy to distinguish from the Hap A and B colours.
Figure 6 has been amended to reflect the colours used throughout the other figures for consistency.

Figures need more informative axes labels.
Axes labels have been updated for Figures 5 – 7 for clarity.

Specific
Figure 2 - adding the full species name would help readability, especially for those not intimately familiar with these species
The full scientific name for each species has been added to the figure.

Figure 4 - it would be helpful to add a label stating how much sequence is added to the NLR loci (20kb)
This has been added to the figure.

Figure 5 and 6 - Consider merging C and D so that all data hapA and B for each chromosome are grouped - the important comparison here seems to be 'how are the counts on chromosomes different across the two haplotypes'.
While I agree part of the focus is the differences between haplotypes, our preference would be to keep these figures as they stand. Merging the graph into differences between haplotypes may reduce the visual representation of the large number of the NLRs that exist within the M. quinquenervia genome.

Figure 6 legend - "D and E" referenced in legend, no D and E in current iteration of figure
This has been corrected.

Figure 7 legend - please replace "(Ref)" with appropriate reference. Add to legend that the count of each domain is included. *I really like this figure
Reference added and legend amended to include details of the count of each domain is included. Thank you, this is one of our favourite figures too!
