# Supplementary material for: A high-quality pseudo-phased genome for Melaleuca quinquenervia shows allelic diversity of NLR-type resistance genes
Source: Gigascience. 2023 Dec 14;12:giad102. doi: 10.1093/gigascience/giad102 (PMC10720953; doi:10.1093/gigascience/giad102)
Supplement: giad102_Reviewer_1_Report_Revision_1 — Andrew Read -- 9/19/2023 Reviewed [file giad102_reviewer_1_report_revision_1.pdf]

Reviewer Report

Title: A high-quality pseudo-phased genome for Melaleuca quinquenervia shows allelic diversity of NLR-type resistance genes

Version: Revision 1 **Date:** 9/19/2023

Reviewer name: Andrew Read

Reviewer Comments to Author:

Thank you for sharing a revised version of the manuscript - it's clear that a lot of work was put into making the changes. I believe that most of my concerns were addressed and am confident that my additional comments can be addressed without another round of review.
Main Text:
Specific
236 - "NLRs require NB and LRR to be functional" - this is not true, the variation observed in NLR modules is described in Monteiro and Nishimura Annual Reviews 2018.
A solution could be to slightly change to say "canonical NLRs require…"
248 - please replace "genes" with "NLR genes" to avoid confusion
295 - delete "novel" - did you check to see if any of your predicted domain coordinates overlap each other? I've seen this in the past and it can lead to artificially high counts. Consider removing the percentages to avoid misleading (unless you did check).
398 - I would consider replacing "no natural enemies" with "where it is exposed to a new suite of microbes" or similar.
417 - I think a more general statement here is appropriate - "this domain may play a role in NLR function"
431 - I appreciate the comment about full-length NLRs, but it seems out of place here. I still think it belongs in "Novel pipeline to identify and classify NLRs" section of the paper.
Figures:
General
The figures that use a unified color palette for haplotype A and B are easier to interpret - consider extending this to all figures. For example, Figure 2 and Figure 6 use different colors than the oranges used in other figures.
Figures need more informative axes labels.
Specific
Figure 2 - adding the full species name would help readability, especially for those not intimately familiar with these species
Figure 4 - it would be helpful to add a label stating how much sequence is added to the NLR loci (20kb)
Figure 5 and 6 - Consider merging C and D so that all data hapA and B for each chromosome are grouped - the important comparison here seems to be 'how are the counts on chromosomes different across the two haplotypes'.
Figure 6 legend - "D and E" referenced in legend, no D and E in current iteration of figure

Figure 7 legend - please replace "(Ref)" with appropriate reference. Add to legend that the count of each domain is included. *I really like this figure

Methods

Are the methods appropriate to the aims of the study, are they well described, and are necessary controls included? Choose an item.

Conclusions

Are the conclusions adequately supported by the data shown? Choose an item.

Reporting Standards

Does the manuscript adhere to the journal's guidelines on minimum standards of reporting? Choose an item.

Choose an item.

Statistics

Are you able to assess all statistics in the manuscript, including the appropriateness of statistical tests used? Choose an item.

Quality of Written English

Please indicate the quality of language in the manuscript: Choose an item.

Declaration of Competing Interests

Please complete a declaration of competing interests, considering the following questions:

- Have you in the past five years received reimbursements, fees, funding, or salary from an organisation that may in any way gain or lose financially from the publication of this manuscript, either now or in the future?
- Do you hold any stocks or shares in an organisation that may in any way gain or lose financially from the publication of this manuscript, either now or in the future?
- Do you hold or are you currently applying for any patents relating to the content of the manuscript?
- Have you received reimbursements, fees, funding, or salary from an organization that holds or has applied for patents relating to the content of the manuscript?
- Do you have any other financial competing interests?
- Do you have any non-financial competing interests in relation to this paper?

If you can answer no to all of the above, write 'I declare that I have no competing interests' below. If your reply is yes to any, please give details below.

I declare I have no competing interests

I agree to the open peer review policy of the journal. I understand that my name will be included on my report to the authors and, if the manuscript is accepted for publication, my named report including any attachments I upload will be posted on the website along with the authors' responses. I agree for my report to be made available under an Open Access Creative Commons CC-BY license (http://creativecommons.org/licenses/by/4.0/). I understand that any comments which I do not wish to be included in my named report can be included as confidential comments to the editors, which will not be published.

Choose an item.

To further support our reviewers, we have joined with Publons, where you can gain additional credit to further highlight your hard work (see: https://publons.com/journal/530/gigascience). On publication of this paper, your review will be automatically added to Publons, you can then choose whether or not to claim your Publons credit. I understand this statement.

Yes Choose an item.
