# Supplementary material for: A high-quality pseudo-phased genome for Melaleuca quinquenervia shows allelic diversity of NLR-type resistance genes
Source: Gigascience. 2023 Dec 14;12:giad102. doi: 10.1093/gigascience/giad102 (PMC10720953; doi:10.1093/gigascience/giad102)
Supplement: giad102_Reviewer_2_Report_Original_Submission — Julia Voelker, Ph.D. -- 6/20/2023 Reviewed [file giad102_reviewer_2_report_original_submission.pdf]

Reviewer Report

Title: A high-quality pseudo-phased genome for Melaleuca quinquenervia shows allelic diversity of NLR-type resistance genes

Version: Original Submission **Date:** 6/20/2023

Reviewer name: Julia Voelker, Ph.D.

Reviewer Comments to Author:

The manuscript about NLR-type resistance genes in two haplotypes of Melaleuca quinquenervia is a relevant contribution to the research of Myrtaceae genomes and other long-lived trees.
The methods are well described and should be reproducible with the available information and raw data, provided the authors mentioned all non-default settings in the method section. The FindPlantNLRs pipeline seems to be well documented on github.
I believe that this manuscript is ready for publication after some small changes. Page and line numbers in the comments below refer to the PDF document:
1. The quality of some figures is not good (even upon download and zoom into the plot) and should be improved to higher resolution for publication. Especially in figure 3, all labels are too pixelated and hard to read. I would also recommend an increase in text size for this figure. In Figure 6 D & E, the authors should consider using consistent text sizes on the axes, and even though the quality is acceptable, a higher resolution of the labels would still be better.
2. p. 10, Table 2: Although it is a standard statistic for genome assemblies, it would be helpful for some readers to specify what N50 and L50 are.
3. p. 19, line 436: I believe the authors are referring to the wrong figure number.
Below are some additional comments regarding typos or other language issues. While the text is generally well written, I would appreciate commas in certain sentences to improve readability, and think that some nouns are missing articles. I hope the authors will read through their text again and add articles where required, I won't point them out individually.
p.4, line 33: wide range of
p.7, line 130: 'a' instead of 8?
p. 8, line 177: genome
p.12, line 250: chromosome 2, add comma before 'while' in next line
p.12, line 253: on all other chromosomes?
p. 13, line 271: to occur?
p.16, line 347: remove 'and'
p.17, line 382, 384: orthologs?
p.20, line 469: 'lead to the triggering of defence response' rephrase to make sense with the previous half of the sentence, also, defence response should have an article
p.20, line 489/490: missing word?

Methods

Are the methods appropriate to the aims of the study, are they well described, and are necessary controls included? Choose an item.

Conclusions

Are the conclusions adequately supported by the data shown? Choose an item.

Reporting Standards

Does the manuscript adhere to the journal's guidelines on minimum standards of reporting? Choose an item.

Choose an item.

Statistics

Are you able to assess all statistics in the manuscript, including the appropriateness of statistical tests used? Choose an item.

Quality of Written English

Please indicate the quality of language in the manuscript: Choose an item.

Declaration of Competing Interests

Please complete a declaration of competing interests, considering the following questions:

- Have you in the past five years received reimbursements, fees, funding, or salary from an organisation that may in any way gain or lose financially from the publication of this manuscript, either now or in the future?
- Do you hold any stocks or shares in an organisation that may in any way gain or lose financially from the publication of this manuscript, either now or in the future?
- Do you hold or are you currently applying for any patents relating to the content of the manuscript?
- Have you received reimbursements, fees, funding, or salary from an organization that holds or has applied for patents relating to the content of the manuscript?
- Do you have any other financial competing interests?
- Do you have any non-financial competing interests in relation to this paper?

If you can answer no to all of the above, write 'I declare that I have no competing interests' below. If your reply is yes to any, please give details below.

I declare that I have no competing interests

I agree to the open peer review policy of the journal. I understand that my name will be included on my report to the authors and, if the manuscript is accepted for publication, my named report including any attachments I upload will be posted on the website along with the authors' responses. I agree for my report to be made available under an Open Access Creative Commons CC-BY license (http://creativecommons.org/licenses/by/4.0/). I understand that any comments which I do not wish to be included in my named report can be included as confidential comments to the editors, which will not be published.

Choose an item.

To further support our reviewers, we have joined with Publons, where you can gain additional credit to further highlight your hard work (see: https://publons.com/journal/530/gigascience). On publication of this paper, your review will be automatically added to Publons, you can then choose whether or not to claim your Publons credit. I understand this statement.

Yes Choose an item.
